# G-quadruplexes as potential traps for superenhancer marker BRD4: ligand-sensitive binding and co-separation *in vitro*

**DOI:** 10.1093/nar/gkaf726

**Published:** 2025-07-31

**Authors:** Iuliia I Pavlova, Olga M Ivanova, Mikhail S Iudin, Anastasiya V Surdina, Nikolay A Barinov, Margarita E Bogomiakova, Sergey D Oreshkov, Zakhar O Shenkarev, Vjacheslav V Severov, Dmitriy V Klinov, Victoria O Shender, Alexandra N Bogomazova, Maria A Lagarkova, Anna M Varizhuk, Vladimir B Tsvetkov

**Affiliations:** Lopukhin Federal Research and Clinical Center of Physical-Chemical Medicine, Malaya Pirogovskaya, 1a, 119435 Moscow, Russia; Lopukhin Federal Research and Clinical Center of Physical-Chemical Medicine, Malaya Pirogovskaya, 1a, 119435 Moscow, Russia; Center for Precision Genome Editing and Genetic Technologies for Biomedicine, Lopukhin Federal Research and Clinical Center of Physical-Chemical Medicine of Federal Medical Biological Agency, Malaya Pirogovskaya, 1a, 119435 Moscow, Russia; Lopukhin Federal Research and Clinical Center of Physical-Chemical Medicine, Malaya Pirogovskaya, 1a, 119435 Moscow, Russia; Lopukhin Federal Research and Clinical Center of Physical-Chemical Medicine, Malaya Pirogovskaya, 1a, 119435 Moscow, Russia; Lopukhin Federal Research and Clinical Center of Physical-Chemical Medicine, Malaya Pirogovskaya, 1a, 119435 Moscow, Russia; Lopukhin Federal Research and Clinical Center of Physical-Chemical Medicine, Malaya Pirogovskaya, 1a, 119435 Moscow, Russia; Shemyakin and Ovchinnikov Institute of Bioorganic Chemistry, Russian Academy of Science, 16/10 Miklukho-Maklaya St., 117997 Moscow, Russia; Shemyakin and Ovchinnikov Institute of Bioorganic Chemistry, Russian Academy of Science, 16/10 Miklukho-Maklaya St., 117997 Moscow, Russia; Lopukhin Federal Research and Clinical Center of Physical-Chemical Medicine, Malaya Pirogovskaya, 1a, 119435 Moscow, Russia; Lopukhin Federal Research and Clinical Center of Physical-Chemical Medicine, Malaya Pirogovskaya, 1a, 119435 Moscow, Russia; Lopukhin Federal Research and Clinical Center of Physical-Chemical Medicine, Malaya Pirogovskaya, 1a, 119435 Moscow, Russia; Center for Precision Genome Editing and Genetic Technologies for Biomedicine, Lopukhin Federal Research and Clinical Center of Physical-Chemical Medicine of Federal Medical Biological Agency, Malaya Pirogovskaya, 1a, 119435 Moscow, Russia; Lopukhin Federal Research and Clinical Center of Physical-Chemical Medicine, Malaya Pirogovskaya, 1a, 119435 Moscow, Russia; Center for Precision Genome Editing and Genetic Technologies for Biomedicine, Lopukhin Federal Research and Clinical Center of Physical-Chemical Medicine of Federal Medical Biological Agency, Malaya Pirogovskaya, 1a, 119435 Moscow, Russia; Lopukhin Federal Research and Clinical Center of Physical-Chemical Medicine, Malaya Pirogovskaya, 1a, 119435 Moscow, Russia; Center for Precision Genome Editing and Genetic Technologies for Biomedicine, Lopukhin Federal Research and Clinical Center of Physical-Chemical Medicine of Federal Medical Biological Agency, Malaya Pirogovskaya, 1a, 119435 Moscow, Russia; Lopukhin Federal Research and Clinical Center of Physical-Chemical Medicine, Malaya Pirogovskaya, 1a, 119435 Moscow, Russia; Center for Precision Genome Editing and Genetic Technologies for Biomedicine, Lopukhin Federal Research and Clinical Center of Physical-Chemical Medicine of Federal Medical Biological Agency, Malaya Pirogovskaya, 1a, 119435 Moscow, Russia; Lopukhin Federal Research and Clinical Center of Physical-Chemical Medicine, Malaya Pirogovskaya, 1a, 119435 Moscow, Russia; Center for Mathematical Modeling in Drug Development, Sechenov First Moscow State Medical University, 8-2 Trubetskaya, 119991 Moscow, Russia

## Abstract

G-quadruplexes (G4s) are prevalent at promoters and superenhancers (SEs), exclude nucleosomes, and recruit transcription factors. This study sought to determine whether the nucleosome exclusion affects the recruitment of the SE marker BRD4, which typically binds to acetylated histones and facilitates SE–promoter contacts via the phase separation-dependent mechanism. Analyses of the available whole-genome data revealed that SEs with the highest G4 density were depleted of nucleosomes but not of BRD4. This led us to test the possibility of histone-independent BRD4 maintenance at G4-rich SEs. A typical SE G4 destabilized a nearby nucleosome *in vitro* and, unlike B-DNA, bound weakly to BRD4 bromodomains. Similar to an acetylated nucleosome, the G4 promoted phase separation in BRD4 solutions. This effect was not altered by the histone competitor JQ1. However, it was attenuated by two known G4 ligands, suggesting that they could disrupt SE–promoter communication in cells. Consistently, these ligands downregulated several genes regulated by G4-rich SE-contacting promoters more efficiently than they did SE-independent genes. Our findings underscore the significance of G4-rich SEs as transcriptional regulators and provide new insights into their organization.

## Graphical Abstract

**Graphical Abstract:**
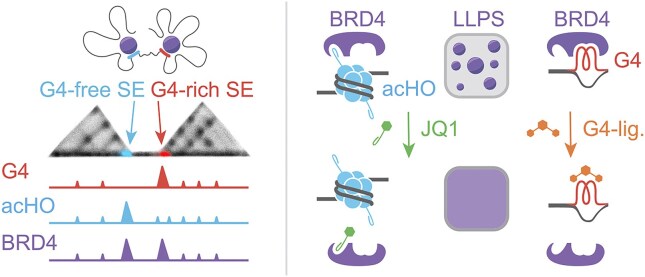

## Introduction

G-quadruplex structures (G4s), whose core is a planar arrangement of Hoogsteen-bonded guanine tetrads, can be formed by the association of G-tract-containing DNA/RNA strands or by the intramolecular folding of a single strand with four G-tracts [1]. Unimolecular G4s have been predicted [2–6] and mapped [7–11] in the genomes of various species, including *Homo sapiens*, and visualized in the nuclei of human cells [12, 13]. They appear to regulate multiple processes related to genome organization, maintenance, and replication [14–16]. Transcription and replication facilitate the formation of G4s, but also require their timely unwinding [17, 18], as persistent G4s on template strands cause polymerase stalling [19]. At the same time, pre-formed G4s at replication origins may assist in the origin licensing and/or replication initiation [20, 21]. Similarly, pre-formed G4s at promoters and enhancers may assist in the transcription initiation [22], presumably by recruiting chromatin remodelers or transcription factors (TFs) [23, 24]. Upon elongation, non-template-strand G4s formed within R-loops may stabilize successive R-loops to enhance transcription [25, 26], allowing for a positive feedback loop. The exact mechanisms behind the impact of G4s on gene expression are likely context-dependent and await further clarification. Promoter G4s have received much attention in this regard [27, 28], whereas enhancer G4s have remained in the background.

The enrichment of G4s at enhancers revealed by chromatin immunoprecipitation and sequencing (ChIP-seq) [29] or cleavage under targets and tagmentation (CUT&Tag) [10] assays with the G4-specific antibody BG4 [12], the correlation between G4s and long-range DNA contacts within topologically associating domains (TADs) or at their boundaries revealed by chromosome conformation capture [30], and the correlation between G4s and Pol II-mediated DNA contacts revealed by chromatin interaction analysis with paired-end tag [31] support the involvement of G4s in the enhancer–promoter communication. This could be achieved through the recruitment of chromatin looping regulators, such as the CCCT-binding factor CTCF [32, 33], which limits DNA extrusion through the cohesin complex [34], or Ying-Yang 1 [35], which stabilizes chromatin loops via dimerization [36]. However, recent studies of the 3D genome architecture at ultra-fine resolution suggest that these proteins are non-essential for maintaining enhancer–promoter contacts [37], so the role of G4s remains an open question.

The enhancer–promoter communication is orchestrated by the Mediator complex [38], which interacts with Pol II and other preinitiation complex (PIC) factors and coactivators. These interactions result in the formation of multicomponent structurally dynamic hubs or condensates [39], supposedly via liquid–liquid phase separation (LLPS) [40]. They are held together by interactions between both structured protein domains and intrinsically disordered regions (IDRs). The Mediator complex has multiple IDRs, but its TF-binding surface is structured. Importantly, the Mediator complex does not contact chromatin directly. Instead, it is recruited to enhancers/promoters by TFs that recognize DNA sequence motifs or histone modifications, typically through structured domains, and form multiple transient contacts with the Mediator complex, typically through IDRs [38]. Thus, the positioning of the Mediator complex at enhancers/promoters is both TF-dependent and drives TF accumulation, allowing programmed but context-dependent assembly of PIC and TF hubs. Whether these hubs are always phase-separated remains a matter of debate [38]. So far, LLPS has only been confirmed at superenhancers (SEs), i.e. long enhancers containing multiple TF-binding sites and communicating with multiple promoters [41].

Of all the TFs involved in the recruitment of the Mediator complex subunits, BRD4, a member of the bromodomain (BD) and extra-terminal (ET) domain protein family (BET), stands out due to its prevalence at SEs [42] and the remarkable propensity for LLPS [41]. BRD4 co-separates with the Mediator subunits due to its long C-terminal IDR [41] and recognizes acetylated histone tails through one or both of its tandem BDs [43]. The bivalent binding mode can be inter- or intra-nucleosomal [44]. An alternative bivalent binding mode, in which the first BD interacts with the acetylated histone, and the second BD binds weakly to DNA, has been demonstrated for a BRD4 homolog BRDT and cannot be excluded for all BET proteins [45]. Anyway, the accommodation of the histone tail in the acetyl-lysine (Kac) binding pocket of at least one BD is essential for BRD4 loading onto chromatin, and the resulting complexes have low to moderate stability at biologically relevant concentrations, depending on the position of the Kac residue within the histone tail [43]. Because promoters and enhancers show different correlations between histone modifications and BRD4 occupancy (e.g. the BRD4–H3K27ac correlation is strong at promoters and weak at enhancers) [44], factors other than chromatin acetylation could be at play. Furthermore, the link between BRD4 and chromatin acetylation must be reciprocal, considering that BRD4 BDs mediate the recruitment of histone acetyltransferases (HATs) [46]. Finally, BRD4 exhibits the intrinsic HAT activity [47]. Its primary targets are histones H3 and H4, and acetylation of the former at K122 results in nucleosome eviction [47].

Should BRD4 remodel chromatin to decrease the nucleosome density, the molecular basis for its maintenance in the chromatin-bound state becomes unclear. Moreover, many promoters, enhancers, and SEs are intrinsically nucleosome-depleted due to the abundance of nucleosome-excluding G4s [11, 22, 48, 49]. Even if the remaining nucleosomes are acetylated, their low density is inconsistent with the maintenance of BRD4 clusters at G4-rich SEs, which is required for LLPS. Therefore, the mechanism of BRD4 accumulation at enhancers/promoters in general and G4-rich SEs in particular requires further investigation. Our aim was to gain some insight into this intriguing matter and we focused on G4-rich SEs. First, we verified G4 enrichment at SEs and G4 colocalization with DNase hypersensitive sites (DHS) and BRD4 peaks within SEs using previously published ChIP-seq/CUT&Tag and DNase-seq data. Next, we identified a consensus motif of the SE G4s and verified the exclusion of acetylated nucleosomes by this motif in a minimal chromatin model. Next, we analyzed the affinity of the consensus SE G4 for BRD4 and its impact on phase separation *in vitro*. Finally, we tested the sensitivity of the resulting biocondensates to G4/BRD4-targeting small molecules, and considered the implications of the observed effects for gene expression regulation.

## Materials and methods

### Bioinformatics

#### Verification of G4 prevalence in SEs

The genome-wide distribution of potential and confirmed G4s relative to SEs was investigated in K-562, HEK-293, and HEP-G2 human cell lines. Potential G4s are those mapped by G4-seq [8] and confirmed G4s are those mapped by ChIP-seq with antibody BG4 [12] in K-562 and HEP-G2 [23] or by CUT&Tag with antibody BG4 in HEK-293 [50]. Processed G4-seq data (4 342 72 peaks) and BG4 ChIP-seq data (8955 high-confidence peaks in K-562 and 8805 peaks in HEP-G2), as well as unprocessed CUT&Tag data were downloaded from Gene Expression Omnibus (accession numbers: GSM3003539, G4-seq; GSE107690, BG4 in K-562; GSE145090, BG4 in HEP-G2; GSE220882, BG4 in HEK-293).

To obtain BG4 peaks in HEK-293, raw CUT&Tag data [50] (accession numbers: SRX20765805, SRX20765806, and SRX20765807) were processed as follows using the Galaxy platform [51]. Sequencing reads were aligned to human genome assembly hg19 (NCBI version 37) using minimap2 [52], and duplicates were removed using Picard tools. Peak calling was performed using the Model-based Analysis of ChIP-Seq (MACS2) algorithm [53] with a *Q*-value cutoff of 0.05, yielding 11 260 high-confidence BG4 peaks, most of which (over 60%) colocalized (±1000 bp) with G4 sites mapped by an alternative technique, namely ChIP-seq with the artificial protein G4P [9].

The SE intervals for all cell lines were downloaded from SEdb 2.0 (http://www.licpathway.net/sedb, accession numbers: Sample_01_0039, K-562; Sample_01_0038, HEP-G2; Sample_02_436, HEK-293). To determine active/poised/repressed SEs, these intervals were intersected with each other and enhancer RNA peaks mapped by cap-analysis gene expression (CAGE) [54] using BEDTools suite [55]. Intervals present in the SEdb dataset for one or two cell lines but not the cell line of interest and containing no CAGE peaks were designated as repressed. CAGE-negative and CAGE-positive intervals present in SEdb for the cell line of interest were designated as poised and active, respectively.

Relative distances between SEs or transcription start sites (TSS) and G4s or randomly selected (control) sites were calculated and plotted using the GenomicDistributions tools from the Bioconductor R package (version 3.18). For each set of G4 sites (potential/confirmed in K-562/HEK-293/HEP-G2), a separate control set with the corresponding site length and number was used. These control sets were generated using the RandomBed tool from the BEDTools suite [55].

To verify G4 enrichment in active/poised/repressed SEs, permutation tests were performed. For each G4 set, the fraction of SE-overlapping intervals was calculated using the Intersect tool from the BEDTools suite and compared to that of the corresponding control set. The procedure was repeated *n* times (*n* ≥ 20), and the statistical significance of G4 enrichment over control was evaluated using Davison and Hinkley’s empirical *P*-value: *P* = (*r*+ 1)/(*n*+ 1), where *r* is the number of replicates in which the control-SE overlap was greater than the G4-SE overlap [56].

#### Identification of enriched G4 motifs

Representative motifs of G4s enriched in K-562/HEK-293 SEs were identified by a three-step procedure. First, the active and poised SEs were sorted based on the frequency or the total number of G4-seq/BG4/G4P peaks. The top 5 SEs in each category were selected and combined. The duplications were removed, and the resulting set included 18 SEs. Second, these 18 SEs were analyzed using G4Hunter [57] to select specific sequences with a G4 folding propensity (score) above the threshold. The default score threshold of 1.2 was used. The resulting sets of high-scoring sequences were concatenated to produce a set of predicted G4s from SEs. Finally, motifs enriched among the predicted G4s from SEs were identified using the MEME algorithm [58]. For two MEME results, namely the leading motif, and the second most abundant motif element, the presence of matching/homologous sequences was verified using the FIMO algorithm [59].

#### Comparison of G4-rich and G4-free SEs

A comparative analysis of the key epigenetic features of G4-rich versus G4-free SEs was performed for the K-562 and HEP-G2 cell lines. While sensitivity to G4-stabilizing ligands and some other features of the SEs may depend on both confirmed (ChIP-seq peaks) and potential (G4-seq peaks) G4 sites, the epigenetic profile must be associated with confirmed G4s in the first place. Therefore, we considered only G4–ChIP-seq data and designated active and poised SEs with the top quartile BG4 peak frequency as G4-rich, and those lacking BG4 peaks as G4-free. The G4-rich and G4-free SEs were compared in terms of their ranks in SEdb 2.0 (http://www.licpathway.net/sedb) and the occupancy of DNAase hypersensitive sites (DHS), BRD4 peaks, or histone marks. Coordinates of DHS previously mapped by DNase-seq were downloaded from Gene Expression Omnibus (accession numbers: GSM816655 in K-562 and GSM816662 in HEP-G2). Coordinates of BRD4 and H3K27ac peaks in HEP-G2 were also downloaded from Gene Expression Omnibus (accession numbers: GSM2635249, BRD4; GSM5866899, H3K27ac). In the case of K-562, only broad BRD4 peaks and (GSM2635249) and raw H3K27ac data (GSM5866899) were available, so the data were (re)processed with MACS2 peak calling as described above. The resulting peaks were intersected with G4-rich or G4-free SEs using the BEDTool suite [55], and the results were summarized as intersection percentage (coverage) plots. Because DHS/BRD4/H3K27ac coverage showed a skewed distribution in one or both SE sets, the significance of the difference between the SE sets was tested by Mann–Whitney *U*-test.

The distribution of BRD4/G4/DHS/H3K18ac peaks in the representative G4-rich and G4-free SEs from the β-globin locus were visualized in the IGV browser, and the Hi-C data [60] were visualized in Encode 3D-genome browser (http://3dgenome.fsm.northwestern.edu/). DNA affinity for histone octamers (HOs) in the G4-free/rich globin SEs was calculated using the nucleosome positioning prediction package of Bioconductor (https://bioconductor.org/packages/release/bioc/html/NuPoP.html).

### Nucleosome assembly and PAGE analysis

Acetylated nucleosomes were assembled on 226-bp double-stranded DNA (dsDNA) constructs which contained the Widom 601 sequence and the 59-bp tail. Two constructs were used: the known G4-free one (Cntr-Widom), previously referred to as construct [1, 48], and the new one with the SE-specific G4 motif A(G_3_A)_4_ in its 59-bp tail (G4-Widom). Both constructs were obtained through ligation of synthetic oligonucleotide precursors as described previously [48]. All oligodeoxyribonucleotides (ODNs) were purchased from Litekh, Russia (purity ≥ 95%, HPLC). Recombinant histones were obtained from Cusabio, China. Nucleosome assembly on Cntr-Widom and G4-Widom was performed in parallel using the sequential dilution method following the previously reported protocol [48] with a minor modification: the unmodified histone H3 was replaced for H3K18ac. Briefly, the 226-bp dsDNA and histones were mixed 1:2 in 20 mM Tris–HCl buffer (pH 8.0) containing 2 M NaCl, and the salt concentration was decreased to 250 mM in four steps with a 30 min incubation at each dilution step. On days 1, 3, and 7 after the sequential dilution, the assembled nucleosomes were visualized in 6% non-denaturing polyacrylamide gel (PAAG) using 100–10 000 bp DNA ladder (Thermo Fisher Scientific, USA) as MW control. The gel was run in a standard Tris-borate-EDTA (TBE) buffer at 4°C and stained with SYBR Green I (Thermo Fisher Scientific, USA). The nucleosome assembly experiments were performed in duplicate.

To verify G4 formation in the G4-Widom tail, a 41-mer tail fragment (dsG4) and its analog from Cntr-Widom (dsCntr), both obtained from Litekh (Russia), were compared by optical methods (described below) and PAGE. For PAGE analysis, the synthetic duplexes were dissolved in the diluted the nucleosome assembly buffer (NAB), optimized for subsequent phase separation assays (10 mM Tris–HCl, pH 8.0, and 125 mM KCl) to a final concentration of 1 μM. To facilitate G4 folding in duplex media [61], the samples were annealed slowly (heated to 90°C and then cooled gradually to room temperature) in the presence or in the absence of the G4-stabilizing ligand pyridostatin (PDS) prior to loading onto 6% non-denaturing PAAG. PDS (Sigma–Aldrich, USA) was used at a final concentration of 20 μM. The gel was run in a standard TBE buffer supplemented with 10 mM KCl and 10% PEG-200 at room temperature and stained with SYBR Gold stain. In all PAGE assays, relative intensities of G4/duplex/nucleosome bands were analyzed in ImageJ.

### Spectroscopy and binding assays

#### Circular dichroism spectroscopy and thioflavin assays

The A(G_3_A)_4_ sequence enriched in SEs (hereafter referred to as SE G4 or simply G4) and the 41-mer duplexes (dsG4 and dsCntr) were dissolved in NAB (20 mM Tris–HCl, pH 8.0, and 250 mM NaCl) or PBS (20 mM sodium-phosphate, pH 7.4, and 140 mM KCl) to final concentrations 1 μM and annealed slowly (dsG4/dsCntr) or rapidly (G4) in the presence or in the absence of the G4-stabilizing ligand PDS (final concentration: 20 μM). Rapid annealing (incubation for 5 min at 90°C and snap-cooling on ice) was used to facilitate unimolecular folding. CD spectra were obtained using a Chrisacan spectrofluorometer (Applied Photophysics, UK) at 20°C. The CD measurements were performed in triplicate, and the spectra were averaged after background subtraction. For ThT assays, thioflavin T (ThT; Sigma–Aldrich, USA) was added to preannealed ODN solutions to a final concentration 5 μM. Fluorescence emission of ThT was measured at 490 nm upon excitation at 450 nm using plate reader Infinite 200 PRO (Tecan, Switzerland) in triplicate.

#### Microscale thermophoresis assays

Recombinant human BRD4 fragment (Glu49–Glu460) with N-terminal His8-tag (Bio-Techne, USA) was labeled with the RED fluorescent dye using RED-tris-NTA kit (NanoTemper Technologies, Germany) according to the manufacturer’s protocol and mixed with 2-fold serial dilutions of the unlabeled acetylated peptide (ac-peptide) or preannealed ODNs in PBS buffer, supplemented with 0.05% Tween-20, to a final protein concentration of 50 nM. The ODNs were purchased from Lytekh (Russia). The ac-peptide, H3K18ac histone fragment (>90% purity, LC-MS), was purchased from Center for collective use of Orekhovich Institute of Biomedical Chemistry, RAMS (Russia). The BRD4–ODN/ac-peptide mixtures were stored at RT for 15 min. Then, MST curves were registered using Monolith NT.115 (NanoTemper, Munich, Germany) at 22°C with RED-mode fluorescence monitoring. The dependence of the normalized fluorescence on the concentration of the ODNs was analyzed using MO.Affinity Analysis software (NanoTemper, Munich, Germany) and fitted to the Hill model. The experiments were performed in triplicate.

#### Surface plasmon resonance assays

Real-time measurement of the BRD4–ODN binding kinetics was analyzed using an iMSPR mini-instrument (Icluebio, South Korea). For BRD4 immobilization, an NTA-Au NiHC1000 sensor chip (Icluebio, South Korea) was activated by injecting 350 μl of 0.5 mM NiCl_2_. Then, 300 μl of 100 nM BRD4 fragment Glu49–Glu460 with N-terminal His8-tag (Bio-Techne, USA) solution in the running buffer (10 mM HEPES, pH 7.4, 150 mM NaCl, 50 μM EDTA, and 0.005% Surfactant P20) was run over the chip at a flow rate of 30 μl/min. After that, 1–200 μM ODN or ac-peptide solution in the running buffer was injected at a flow rate of 30 μl/min at 24°C. The experiments were performed in duplicate for each cycle (i.e. each ODN/ac-peptide concentration). For dsG4, additional experiments were performed in the presence of 4 eq. of the G4 ligand PDS (Sigma–Aldrich, USA) or BRD4 ligand JQ1 (Abcam, UK). The sensor chips were regenerated between the cycles by injecting 50 mM imidazole to remove surface-bound BRD4 and its complexes with the ODNs/ac-peptide. The association/dissociation constants (*k*_a_/*k*_d_) and the equilibrium constant (*K*_d_ = *k*_d_/*k*_a_) were calculated using the iMSPR analysis software (Icluebio, South Korea).

#### Nuclear magnetic resonance spectroscopy

Nuclear magnetic resonance (NMR) experiments were performed using the 24 μM solution of the non-labeled G4 ODN in 20 mM Na-phosphate buffer, pH 7.5, supplemented with 80 mM KCl and 5% (v/v) D_2_O. The 1D ^1^H spectra were acquired on the Bruker Avance 700 spectrometer equipped with a cryoprobe at 25°C. The spectra were recorded using excitation sculpting water suppression scheme (pulse program zgesgp in Bruker library) with following parameters: spectral width of 17.5 kHz; repetition delay of 1.5 s; 32 768 complex points; 3200 scans per FID; measurement time per one spectrum of 132 min. Despite rapid annealing of the sample (incubation for 5 min at 90°C and snap-cooling on ice) prior to the experiment, performed to facilitate unimolecular folding, partial aggregation of the ODN was evident from the presence of a broad signal (hump) at 11.0–11.5 ppm. The fraction of the unimolecular G4 (∼50%) was estimated by calibrating intensities of individual G4 imino-proton signals (I–V) using the standard NMR sample with known concentration as a reference. For the BRD4-binding assay, a stock solution of recombinant BRD4 fragment Glu49–Glu460 (Thermo Fisher Scientific, USA) in the storage buffer (50 mM HEPES, pH 7.5, 200 mM NaCl, 10% glycerol, and 1 mM dithiothreitol, DTT) was added to the preannealed ODN sample to a final protein concentration of 8.4 μM and an 8% admixture of the storage buffer. Addition of the protein resulted in the decrease of the intensities of individual G4 imino-proton signals. The linewidth and position of the G4 signals were not affected. We assume that exchange of G4 between free in solution and BRD4-bound states was slow (on the NMR timescale) and the signals of BRD4-bound G4 were unobservable in the NMR spectra due to the relatively large mass of the protein (MW = 52 kDa). In this approximation the intensities of the observed G4 signals (I–V) are proportional to the concentration of free G4 in solution ([G4]_free_). Control experiments included titration of the ODN sample with equal volumes of blank solution (protein-free storage buffer); its effect on the G4 spectrum was minor. Protein-induced changes in the intensities of the individual G4 imino-proton signals were normalized by those induced by the storage buffer and fitted into the model with single parameter *K*_d_ = [G4]_free_ × [BD]_free_ / [BD · G4], where [BD]_free_ and [BD · G4] are the concentrations of free and G4-bound bromodomains in the sample. A two-site binding model (each BRD4 molecule contains two BD and can bind two G4s) provided better agreement with experimental data than simple 1-to-1 binding. The Mathematica 12.0 software was used (Wolfram Research, Champaign, IL, USA).

### Molecular modeling

The 3D models of ac-peptide (H3K18ac fragment S10-S28), SE G4, dsG4, and dsCntr were constructed using the molecular graphics software package SYBYL-X (Certara, USA). The dsG4 model was obtained using an approach developed by Tsvetkov V.B. [62]. Briefly, it was created by modifying the previously reported NMR-based model of a parallel-stranded G4 (PDB: 2LEE) [63]. The G4-flanking residues were removed, and C in the first loop was replaced with an A. Then, prearranged models of 12 bp duplex flanks and the G4-opposing C-rich loop were added. The resulting structure was optimized using SYBYL-X (Certara, USA) and Powell’s method. Further details are provided in Supplementary Text Box S1.

The BRD4 BD models were taken from the reported structures of their complexes with peptides: PDB: 2UVW [43] (BD1) and PDB: 6U6L [64] (BD2). The peptides were removed, and ac-peptide/G4/dsG4/dsCntr were docked to the free BD surface in ICM-Pro 3.9.2 software [65]. For ac-pepide, flexible ligand docking was employed. The resulting complexes were then ranked based on the docking scoring function and additionally screened for K18 positioning within its cognate binding site, namely the BD hydrophobic pocket.

In the cases of G4, dsG4, and dsCntr, a two-step procedure, proposed by Tsvetkov V.B. [66], was selected to ensure the maintenance of the DNA secondary structures. The procedure included rigid ligand docking followed by molecular mechanics (MM) optimization using the Amber 7ff02 force field and SYBYL-X software (Certara, USA). The stability of the resulting complexes was verified by explicit solvent molecular dynamics (MD) simulation using Amber 22 software and energies of the complexes were assessed using the molecular mechanics with Generalized Born surface area (MMGBSA) approach.

The potential competition between JQ1 and dsG4 for BRD4 BDs was verified by additional docking experiments: JQ1 was docked to the BRD4–dsG4 complex (the most likely conformation, in which G4 occupies BD2) and dsG4 was docked to the BRD4–JQ1 complex (the previously reported one, in which JQ1 occupies BD1, PDB: 4QZS). Details on MD, MMGBSA energy calculations, and additional docking are provided in Supplementary Text Box S1.

### Biocondensate assembly and microscopy imaging

#### Sample preparation

His-tagged full-length BDR4 (BRD4-5928h, Creative BioMart, USA) was labeled with the RED fluorescent dye using RED-tris-NTA kit (NanoTemper Technologies, Munich, Germany) and mixed 0.15:1 with unlabeled protein in PBS (pH 7.4), supplemented with 20% PEG-400, to a final protein concentration of 4 μM. Then, ODN, DNA-free H3K18ac, or histone-free DNA were added to final concentrations of 4 μM, 150 nM, or 300 nM, respectively. Nucleosomes were added to a final DNA concentration of 150 nM and acetylated histone octamer (ac-HO) concentration of 300 nM. The BRD4 inhibitor JQ1, G4 ligand PDS, G4 ligand SOP1812, or 5-fluorouracyl (5FU), selected as a negative control, were added to final concentrations of 5 μM, 20 μM, 5 μM, or 1 mM, respectively, with dimethyl sulfoxide (DMSO) admixtures, and compared to a blank DMSO sample. The final DMSO concentration in all samples was 0.5%. 1,6-Hexandiol (HD), the known inhibitor of hydrophobicity-dependent phase separation selected as a positive control, was added to a final concentration of 10%. HD, 5FU, and PDS were obtained from Sigma–Aldrich (USA), JQ1 was obtained from Abcam (UK), and SOP1812 was obtained from MedChemExpress (USA).

#### Fluorescence microscopy of BRD4 mixtures with DNA/histones/ligands

BRD4 mixtures with DNA/histones/ligands or the control samples were incubated for 15 min at room temperature, then placed into the working chamber of 0.5 mm height between the Pyrex glass slides (Corning, USA), and visualized using Eclipse Ti2 microscope (Nikon, Japan) upon RED excitation in the 590–650 nm band with a cut-off wavelength of 660 nm. All experiments were performed at least in three biological repeats. In each experiment, the volume fraction of the stained droplets was calculated based on the total droplet projection area and assuming spherical droplet shape. The total projection area and the average droplet circularity per image were determined using Droplet_Calc software [https://github.com/biopolymers-lab-FRCC-PCM/droplets_area_calculation/tree/main] with default settings.

#### Confocal microscopy and fluorescence recovery after photobleaching

For verification of dsG4 colocalization with BRD4 upon phase separation, the G4-containing strand of dsG4 was labeled at 5′-termini with a 6-carboxyflorescein (FAM) residue, mixed with unlabeled strand, and annealed slowly in diluted NAB (10 mM Tris–HCl, pH 8.0, and 125 mM NaCl) supplemented with 20% PEG-400. Then, (FAM)–dsG4 was mixed with (RED)-BRD4 (labeled: unlabeled = 0.15:1) in the same buffer to final DNA and protein concentrations of 4 μM. The samples were prepared in the presence or in the absence of 20 μM PDS to verify PDS effects on phase separation. The samples were incubated for 15 min, then loaded onto microscopy plates, pretreated for rapid condensate fixation as previously reported [67, 68], and stored for additional 15–30 min to allow for gradual condensate settling by gravity.

Pretreatment of the plates included methoxy-PEG (mPEG) coating similar to that described previously [67, 68]. The coating was performed in three steps. Chemically polished Pyrex glass slides (Corning, USA) were first coated with 3-aminopropyltriethoxysilane (Sigma–Aldrich, USA), then further functionalized with *N,N*′-disuccinimidyl carbonate (Sigma–Aldrich, USA), and eventually treated with methyl- and amine-terminated polyethylene glycol MA(PEG)24 (Thermo Fisher Scientific, USA). All procedures were performed following previously published protocols [69, 70] with a single modification at the final step (MA(PEG)24 was used instead of the protein). The mPEGylated glass slides were passivated with BSA as described previously [67].

Confocal microscopy imaging of the (FAM)–dsG4/(RED)–BRD4 mixtures was performed using Olympus FV3000 microscope (Olympus Scientific Solutions, USA) in FAM and RED channels (laser wavelengths: 488 and 640 nm, respectively; cut-off filters: 500 and 650 nm, respectively). In fluorescence recovery after photobleaching (FRAP) assays, three condensate/gel segments in each sample type (with or without PDS) were bleached, and time lapse series were recorded with a 10 s time span. The experimental fluorescence recovery curves were averaged and fitted to a mono-exponential equation.

#### Atomic force microscopy of BRD4 mixtures with DNA and histones

The BRD4-nucleosome mixtures used for fluorescence microscopy imaging, the control samples (DNA-free nucleosomes and nucleosome-free BRD4), and the BRD4-dsG4/dsCntr mixtures with/without PDS in the same buffer (diluted NAB with 20% PEG-400) were diluted 100-fold and applied onto the freshly cleaved graphite surface rendered hydrophilic by pretreatment with an amphiphilic reagent (CH_2_)*_n_*(NCH_2_CO)*_m_*-NH_2_. In each experiment, a 5 μl of sample drop was kept on the modified graphite surface for 5–15 s, and then removed with a nitrogen stream. Atomic force microscopy (AFM) images were obtained in air using home-made super-sharp cantilevers [71] and a multimode AFM instrument with an NTEGRA Prima controller (NT-MDT, Russia) in the tapping mode with a typical scan rate of 1 Hz and a typical free amplitude of several nm. The AFM data were filtered and analyzed using the FemtoScan Online software (ATC, Russia), and then flattened using standard algorithms (subtraction of the quadric surface and averaging by lines). The experiments were performed in duplicate for the G4/Cntr-Widom series and at least in triplicate for the dsG4/dsCntr series. Analysis of the apparent heights/radii of BRD4 and ac-HO granules, as well as the calculation of dsG4 granules inside/outside condensate precursors, were performed using Gwyddion software, version 2.58.

### Analysis of gene regulation

#### Cell cultures and toxicity assays

Human embryonic kidney (HEK-293) immortalized cells were cultured in a humidified 5% CO_2_ incubator at 37°C, tested for mycoplasma contamination, and grown in 96-well culture plates in Dulbecco's Modified Eagle Medium (DMEM) (Paneco, Russia) supplemented with a 1% penicillin/streptomycin mixture (Paneco, Russia), 2 mM glutamine (Paneco, Russia), and 10% fetal bovine serum (HyClone GE Healthcare, USA). Human myeloid leukemia (K-562) cells were grown in RPMI-1640 medium (Paneco, Russia) supplemented with 10% fetal bovine serum (HyClone GE Healthcare, USA). In toxicity assays, cell viability was tested after 48 h of incubation with increasing concentrations of PDS, SOP1812, or 5FU using a PrestoBlue reagent (Thermo Fisher Scientific, USA). The color change was detected by measuring absorbance at 570 nm using plate reader Infinite 200 PRO (Tecan, Switzerland).

#### RNA isolation and reverse transcription

For gene regulation assays, PDS and 5FU were added to HEK-293 or K-562 cells (4 × 10^5^ cells in 10 ml of culture medium) to final concentrations equal to IC30 values (10 μM PDS, 1.25 μM SOP1812, or 50 μM 5FU for HEK-293 and 20 μM PDS, 10 μM SOP1812, or 100 μM 5FU for K-562). After 48 h incubation, the cells were harvested. Total RNA was isolated using RNeasy Mini Kit (Qiagen, USA) following the manufacturer’s instructions including the DNase treatment step. The quantity of the resulting RNA was evaluated and the purity was confirmed by measuring absorption at 260 nm/280 nm using NanoQuant plates and plate reader Infinite 200 PRO (Tecan, Switzerland). Reverse transcription was performed as follows: first-strand complementary DNA (cDNA) was synthesized from 4 μg of RNA per sample using Magnus reverse transcriptase (Evrogen, Russia) using 40 pmol of random decamer primer (Evrogen, Russia) following the manufacturer’s instructions. The synthesis of cDNA for all samples was performed in parallel.

#### Selection of target genes

The major gene set selected for RT-qPCR-based verification of the SE-dependent versus promoter-dependent gene regulation included protooncogenes encoding kinases and other signaling-related proteins (*PLK3, MKNK2, RAPGEFL1, IFI6*, and *WASF2*), the nuclear receptor *NR1D1*, the histone methyltransferase *DOT1L*, and the uroporphyrinogen decarboxylase *UROD* (a chemotherapy sensitizer). All of them contained BG4 ChIP-seq peaks in promoter regions, which overlapped SEs or coincided with the hotspots of long-range contacts with SEs. The control genes, namely the syntaxin-encoding gene *SNTX12* and the proteasome subunit-encoding gene *PSMD3* also contained BG4s peaks in promoter regions but did not form long-range contacts with SEs or other regulatory sites with BRD4 clusters within TAD. The ribosomal RNA-encoding genes *RPLP0* and *RPS18*, selected as a reference, had G4-free promoters. The TAD boundaries and intra-TAD contacts were analyzed based on the available Hi-C data [60] and visualized in Encode 3D-genome browser (http://3dgenome.fsm.northwestern.edu/). The additional gene set included genes with inapparent 3D contacts (below average frequency of contacts with SEs) but well-defined BG4 peaks in promoters.

#### RT-qPCR assays and correlation analysis

RT-qPCR was performed using qPCRmix-HS SYBR (Evrogen, Russia). For each reaction, 1.5 μl of the cDNA was mixed with 5 pmol of the respective primers and 4 μl of qPCRmix-HS SYBR. Then, RNase-free water was added to a total volume of 25 μl, and PCR was carried out in 45 cycles (95°C for 3 min, then 95°C for 10 s, 60°C for 20 s, and 72°C for 30 s per cycle) using CFX96 Touch RT PCR Detection System (Bio-Rad, USA). Amplicon quality was verified by melting curves analysis. The results were analyzed by the ΔΔCq method using *RPLP0* and *RPS18* as reference genes. All experiments were performed in two biological and three technical repeats.

The ligand-induced regulation was presented as log_2_FC. Genes with *P* < 0.05 and log_2_FC > 2 and Log_2_FC < -2 were regarded as up-regulated and down-regulated, respectively. The significance of the correlations between PDS-induced regulation and promoter features (BRD4/BG4/G4-seq peaks scores) was tested for all genes with *P* < 0.05, namely the 10 genes from the major set and the 10 genes from the additional set. For the main gene set with well-defined SE–promoter contacts or the confirmed absence of such contacts, a similar correlation analysis was performed for SE features. The BRD4/BG4/G4-seq peak scores were obtained from the respective ChiP-seq/G4-seq datasets (GSM3003539, GSE107690, and GSE99178).

## Results

### G4s are frequent in active SEs that are depleted of nucleosomes but not of BRD4

The prevalence of G4s in human *cis-*regulatory sites [22], including enhancers [29, 72], has been reported for various cell types. However, the significance of G4 enrichment in SEs has only been clearly demonstrated in stem cells [73]. The abundance of G4s in SEs of human embryonic kidney (HEK-293) cells and several cancer cell lines has been partially analyzed, but with a focus on common enhancers [29]. We re-examined this question focusing on SEs in three cell lines, namely HEK-293, HEP-G2 hepatocellular carcinoma, and K-562 myeloid leukemia cells.

First, we analyzed the distribution of potential and confirmed G4 sites relative to established SEs available in SEdb 2.0 [74] for the cell lines of interest (Supplementary Fig. S1A). Potential G4 sites were G4-seq peaks [8], and confirmed sites were ChIP-seq (HEP-G2 and K-562) or CUT&Tag (HEK-293) peaks obtained using the BG4 antibody (BG4 peaks) [23, 75]. In all cell lines, overlaps of BG4 with SEs were more frequent than those of control (random) sites (*P* < 0.05 and Supplementary Fig. S1B). The enrichment of BG4 over control in HEP-G2 SEs (6 ± 5) was close to that of G4-seq in all SEs (2.5 ± 0.2). In HEK-293 and K-562 SEs, the enrichment of BG4 over control was substantial (30 ± 20), comparable to the enrichment in general promoters (TSS ± 1000 bp), but ∼10-fold lower than in core promoters (TSS ± 100 bp).

**Figure 1.**
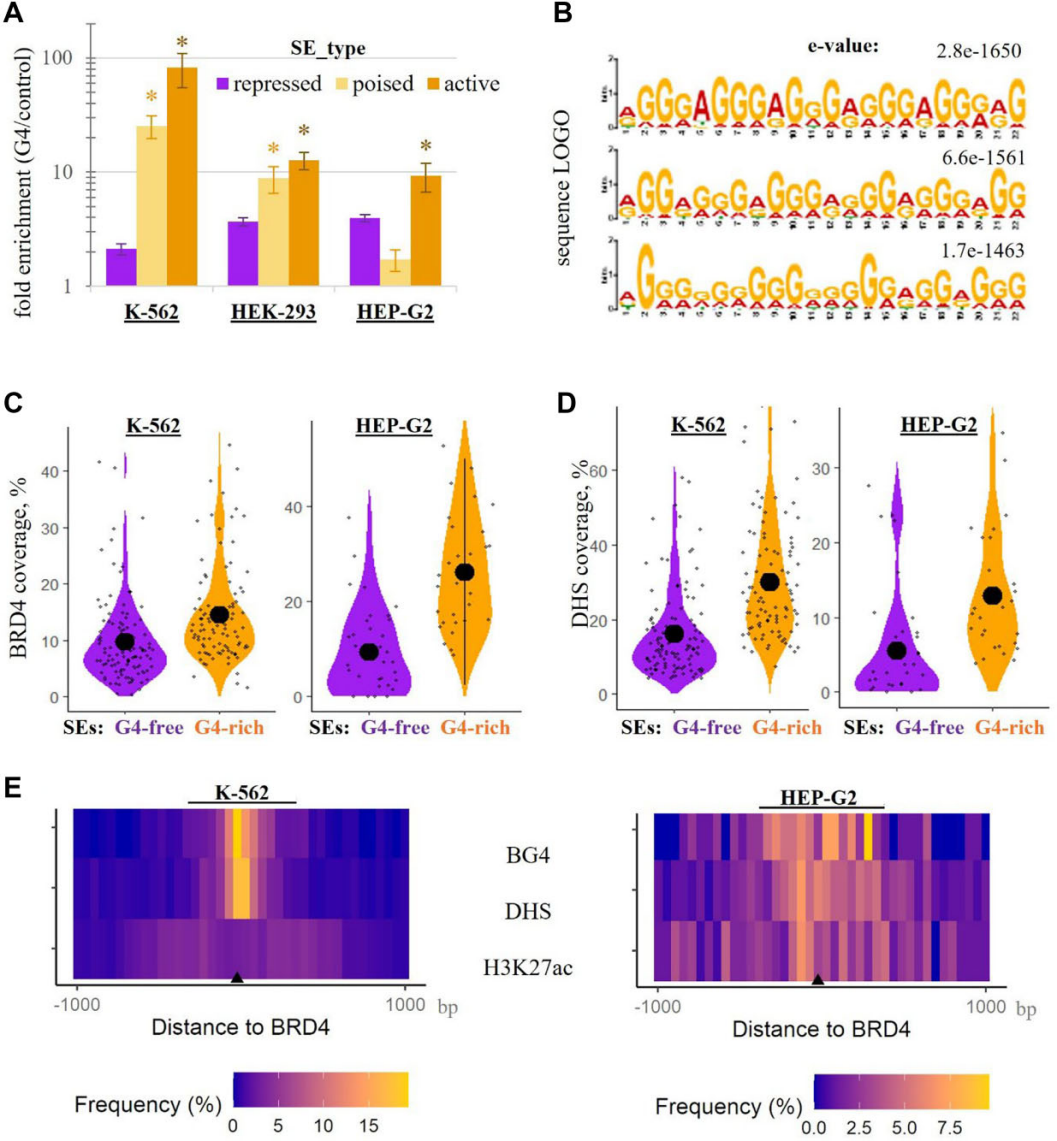
Whole genome analysis of G4s in SEs: enrichment, representative motifs, and distribution relative to BRD4-binding sites. (**A**) G4 enrichment in SEs of various status (permutation tests). The SE-G4 overlap was calculated using G4 ChIP-seq/Cut&Tag data obtained with the BG4 antibody and cell line-specific SE coordinates obtained from SEdb 2.0. For each cell line, the SE-G4 overlap was compared to that of the control set of random sites of equal size and length. Results are shown as mean ± SD fold enrichment of G4s over control sites for repressed, poised, and active SEs. Active SEs are those present in SEdb 2.0 and confirmed by CAGE. Poised SEs are those present in SEdb 2.0 but lacking CAGE peaks. Repressed SEs are those absent in the cell line of interest but present in other cell lines, according to SEdb 2.0. *Significant difference between repressed and poised/active SEs (*P* < 0.05, Student’s test). (**B**) Motifs enriched in a combined set of G4 sites from K-562 and HEK-293 G4-rich SEs (top-scoring MEME results). (**C**) Comparison of active G4-free and G4-rich SEs: normalized coverage of BRD4 ChIP-seq peaks. G4-free SEs are those lacking BG4 peaks (116 in K-562 and 36 in HEP-G2), and G4-rich ones are those showing top quartile BG4 coverage (100 in K-562 and 32 in HEP-G2). (**D**) Normalized DHS coverage in active G4-free and G4-rich SEs. In panels (C) and (D), the difference between G4-free and G4-rich SEs is statistically significant in all cases (*P* < 0.05, Mann–Whitney test). (**E**) Distribution of BG4, DHS, and H3K27ac peaks relative to BRD4 occupancy sites in active SEs.

As the observed enrichment variance between cell lines could be related to the variance in SE activity, we investigated the SE inclusion criteria in more detail. Canonical criteria include the presence of multiple TF-binding sites and H3K18ac histone marks. In SEdb 2.0, the fulfilment of these non-stringent criteria is summarized as SE rank. A more stringent criterion is the presence of enhancer RNA detected by nuclear run-on assays or cap-analysis gene expression (CAGE) [54]. For each cell line of interest, we designated CAGE-positive SEs from SEdb 2.0 as active (401 out of 686 in K-562, 310 out of 538 in HEK-293, and 120 out of 505 in HEP-G2). The remainder were designated as poised SEs. SEs that were present in one or two cell lines, but not in the cell line of interest, were designated as repressed. The analysis of G4 coverage in active/poised/repressed SEs is summarized in Supplementary Fig. S2A (absolute values) and Fig. 1A (permutation tests). The enrichment of confirmed G4s in HEK-293 and K-562 poised SEs was significantly higher (*P* < 0.05) than that in repressed SEs. For active SEs, the enrichment was significant in all cell lines of interest (Fig. 1A).

Next, we questioned whether typical SE G4s differ from G4s that are prevalent genome-wide. From the partially overlapping sets of SEs with the highest peak count and frequency of potential and confirmed G4s (Supplementary Table S1), we selected sequences with substantial (above-threshold) G4 folding propensity using G4Hunter [57] and identified the enriched motifs using the MEME algorithm. Both active and poised SEs from the K-562 and HEK-293 cell lines were included in this analysis (Supplementary Table S1). However, the top scoring motif (Fig. 1B), hereafter referred to as SE G4, was particularly abundant in active SEs, as shown by FIMO results (Supplementary Table S2). This motif reportedly adopts an all-anti G4 topology with propeller-type loops and G-tracts in parallel orientation [76]. It corresponds to one of the most abundant putative G4 sequences genome-wide, consisting of 3-nucleotide G-tracts separated by single-nucleotide A/T loops [3, 77]. We conclude that parallel-stranded G4s with A-loops are particularly abundant in SEs but are not SE-specific.

To explore the potential significance of the G4 enrichment, we compared active SEs with top quartile and zero BG4 coverage (Supplementary Fig. S2). These SE sets are hereafter referred to as G4-rich and G4-free, respectively. They had similar rank distributions in all cell lines of interest. We focused on K-562 and HEP-G2, for which comprehensive data on SE marker distribution were available (Supplementary Fig. S2). In both cell lines, BRD4 peaks, DHS, which mark nucleosome-depleted regions, and the BRD4-associated mark H3K27ac [44] on the remaining nucleosomes were significantly more abundant in G4-rich active SEs than in G4-free active SEs (*P* < 0.05, two-tailed *U*-test, Fig. 1C and D, and Supplementary Fig. S3A). An analogous analysis of the joint sets of poised and active SEs gave similar results (Supplementary Fig. S3B). We conclude that G4s may exclude nucleosomes but not the histone mark reader/writer BRD4 from SEs.

Further examination of G4-rich active SEs revealed colocalization of BRD4 with confirmed G4s and DHS peaks, while H3K27ac peaks clustered in the flanking regions (± 200 – 500 bp), at least in K-562 cells. In HEP-G2, the general trend was less apparent due to a small number of G4-rich SEs (Fig. 1E). An analogous analysis of the joint sets of poised and active G4-rich SEs gave similar results (Supplementary Fig. S3C). In G4-free SEs (Supplementary Fig. S3C), major clusters of H3K27ac remained shifted from BRD4, but their frequency within 100 bp from BRD4 was increased by 83% (K-562) or 25% (HEP-G2) compared to G4-rich SEs. In contrast, DHS frequency within 100 bp from BRD4 was decreased by 54% (K-562) or 15% (HEP-G2). We conclude that the context or consequences of BRD4 positioning on chromatin are slightly different in G4-rich SEs and G4-free SEs.

**Figure 2.**
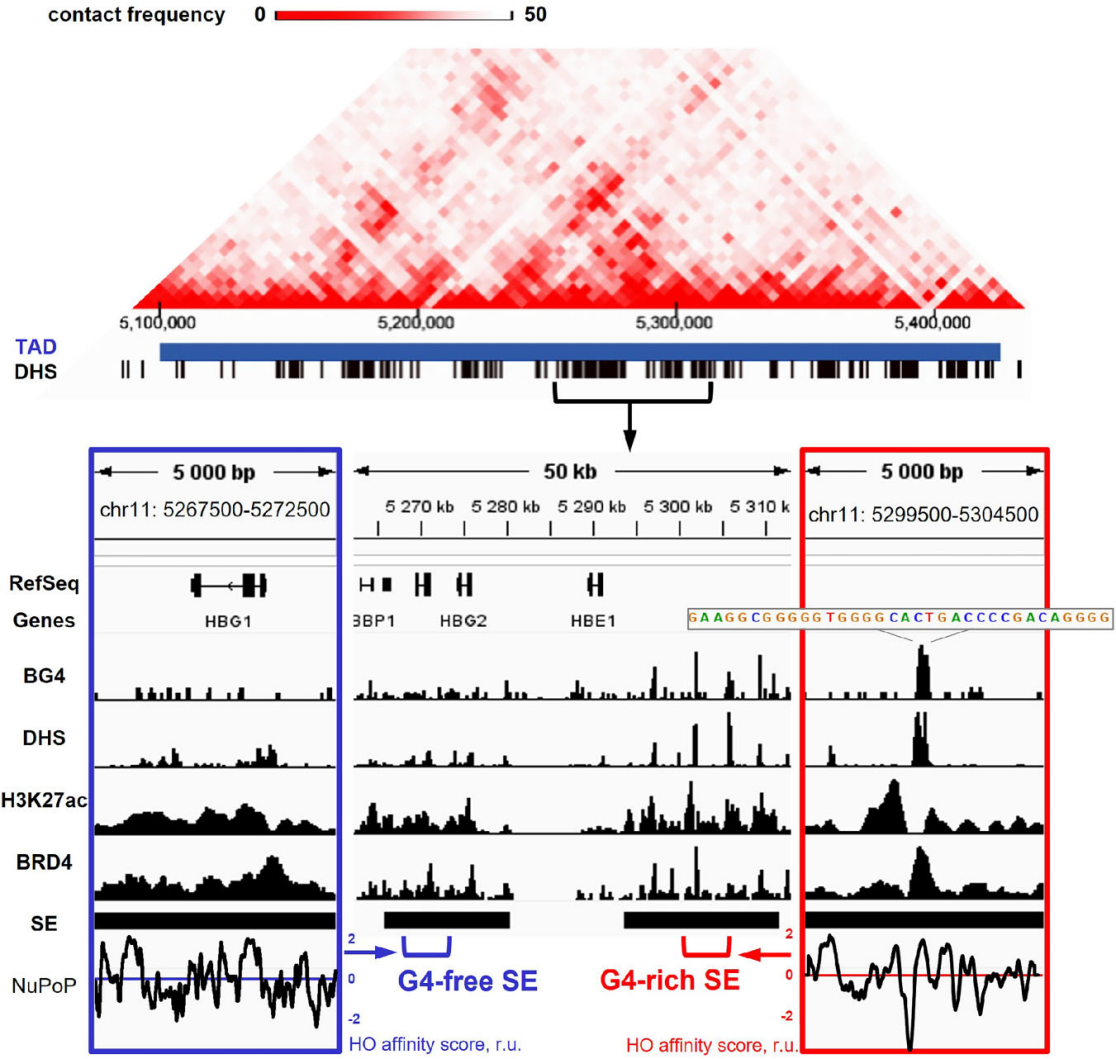
Examples of G4-free and G4-rich SEs from the human β-globin locus. 3D contacts within the locus-containing TAD (Hi-C data) and the distribution of BG4/DHS/H3K27ac/BRD4 peaks (ChIP-seq and DNase-seq data) in its central region, encompassing fetal (HBG1 and HBG2) and embryonic (HBE1) globin genes with respective SEs, are shown for the K-562 cell line. Side panels are representative fragments of the G4-free (left) and G4-rich (right) SEs. The bottom plots illustrate sequence-based predictions of DNA affinity for HOs obtained using the nucleosome positioning prediction tool (NuPoP). The gray box in the right panel illustrates the presence of a G-rich motif within the BG4 peak colocalized with BRD4 and DHS peaks.

An example of the different SE types and BRD4 positioning patterns can be found in the β-globin gene locus from the chr 11 TAD (Fig. 2). Its central part contains the embryonic (HBE1) and fetal (HBG2 and HBG1) hemoglobin subunit genes, which show stable and condition-dependent expression in K-562, respectively [78]. The embryonic gene is activated by the upstream SE, also known as the locus control region [79], which contains a DHS cluster and a G4 cluster with two high-confidence BG4 peaks in K-562. This globin SE is hereafter referred to as G4-rich. The fetal genes overlap another DHS cluster-containing SE and are activated by the HGB-4kb regulatory site therein [80]. This globin SE does not contain high-confidence BG4 peaks and is hereafter referred to as G4-free.

The H3K27ac and BRD4 peak coverage in the G4-rich globin SE (21% and 5%, respectively) are almost 2-fold higher than in the G4-free globin SE (12% and 3%). Importantly, despite the similar overall DHS coverage (∼10%), the SEs show distinct DHS distribution. In the G4-free SE it agrees reasonably well with sequence-based nucleosome positioning predictions (NuPoP), whereas in the G4-rich SE the DHS peaks are slightly shifted away from the predicted nucleosome-free sites (negative peaks in the histone affinity score profile) and tend to colocalize with G4 sites, such as the representative BG4 peak in Fig. 2. Importantly, this peak also colocalizes with the BRD4 peak, flanked by H3K27ac.

To summarize, the whole-genome analysis (Fig. 1) and the inspection of the β-globin locus in K-562 (Fig. 2) suggest that G4s are incompatible with nucleosomes, including acetylated ones, but this does not prevent BRD4 recruitment or maintenance, arguing for possible BRD4–G4 interactions. Direct binding of BRD4 or other BET proteins to nucleosome-free B-DNA has not been previously observed, whereas G4 DNA has shown moderate affinity for BRD4 homologs [66]. In this regard, we next tested the exclusion of acetylated nucleosomes by the G4 DNA and BRD4–G4 interactions in a simplified *in vitro* model.

### SE G4 destabilizes an acetylated histone octamer and binds weakly to BRD4 *in vitro*

The exclusion of unmodified nucleosomes by promoter G4s and model G4s *in vitro* has been shown previously [48, 49]. Here, we tested the impact of the consensus SE G4 (Supplementary Table S3) on the assembly of an acetylated nucleosome in a minimal chromatin model [48], namely the 226 bp Widom 601-based sequence, consisting of a 167 bp nucleosome positioning core and a 59 bp tail with the G4 sequence in the middle (G4-Widom). The G4 was introduced into the tail sequence because in the core sequence it would disrupt the ∼10 bp periodic dinucleotide pattern required for histone binding [81] and thus exclude the nucleosome in a sequence-dependent rather than structure-dependent manner. In the 226 bp control DNA (Cntr-Widom), the 15 bp G4 motif (G_3_A)_3_G_3_ was replaced by the non-G4 sequence TTCAACCAGTCTATG.

**Figure 3.**
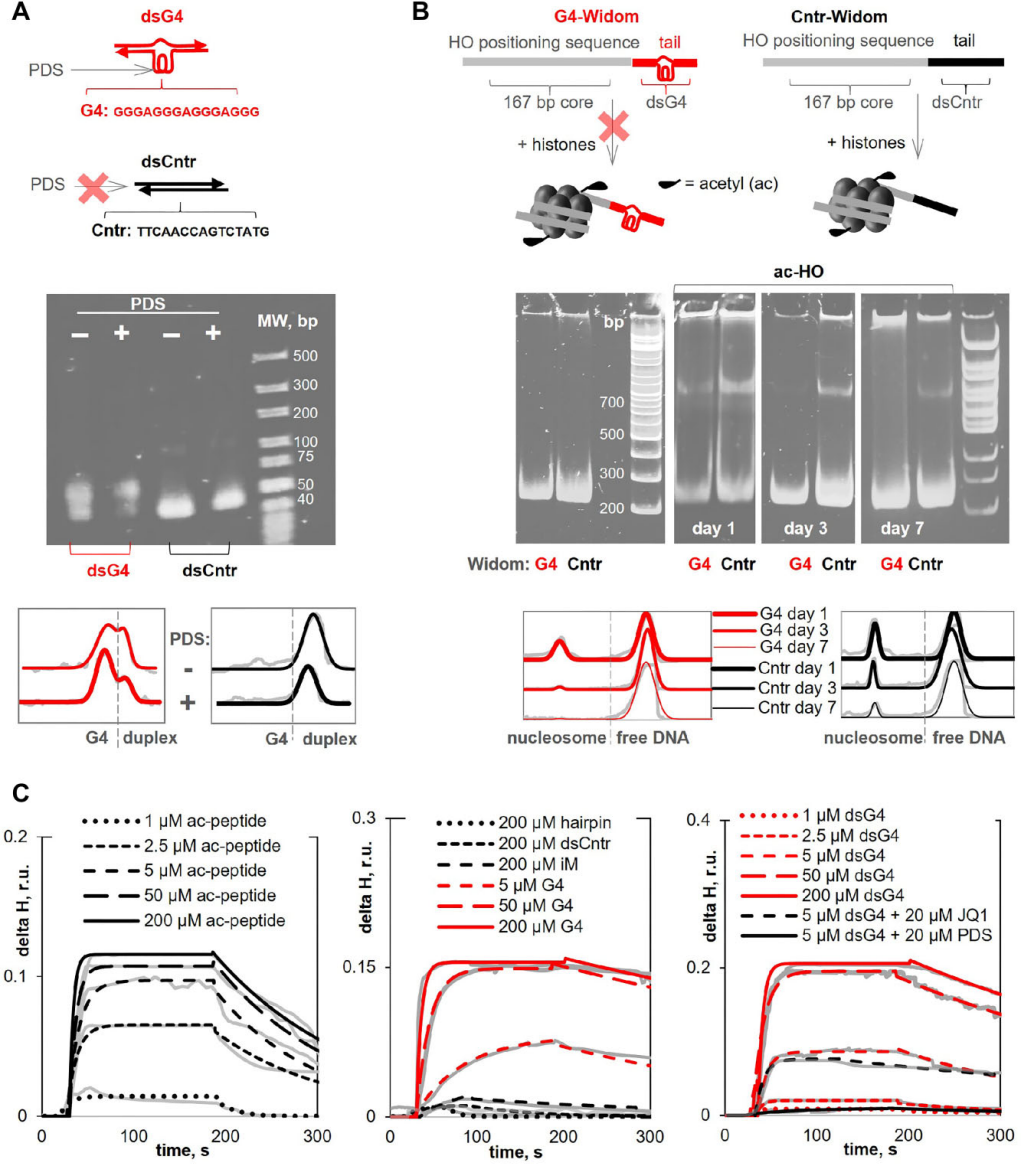
Effect of the SE G4 on nucleosome assembly and its interactions with BRD4 *in vitro*. (**A**) Schematic representation of the SE G4-containing duplex (dsG4) or the non-G4 control (dsCntr) and PAGE verification of their sensitivity to the G4 stabilizing ligand PDS. Annealing conditions: 1 μM dsG4/dsCntr and 0/10 μM PDS in 10 mM Tris–HCl buffer (pH 8.0), supplemented with 125 mM KCl and 20% PEG. PAGE conditions: 6% non-denaturing PAAG, 1× TBE with 10 mM KCl, and 10% PEG, SYBR Gold stain. The plots in the bottom illustrate the AUC-based analysis of the folded G4 fraction (experimental, gray; Gauss fitting). (**B**) Schematic representation of the Widom DNA fragments with the G4-tail (G4-Widom) or the non-G4 control (Cntr-Widom) and PAGE analysis of the assembly of H3K18ac-containing histone octamer (ac-HO) on these DNA. The plots in the bottom illustrate the AUC-based analysis of nucleosome fraction after 1–7-day incubation (experimental, gray; Gaussian fitting). (**C**) SPR-based analysis of BRD4 interactions with the acetylated peptide (ac-peptide) from H3K18ac (aa 10–28), G4, dsG4 and control ODNs (1–200 μM in the SPR running buffer). The sensograms are overlayed with monoexponential fitting.

First, we verified the folding of G4-Widom and Cntr-Widom tails using truncated (41 bp) duplexes dsG4 and dsCntr, respectively (Fig. 3A). The latter showed a circular dichroism (CD) spectrum typical of B-DNA with a major positive band at 280 nm. The dsG4 spectrum was a superposition of B-DNA and G4 DNA signatures, i.e. it contained an additional positive band at 265 nm (Supplementary Fig. S4A). The amplitude of the 265 nm band was slightly increased in the presence of the G4 stabilizing ligand PDS [82], whereas dsCntr showed no PDS-induced CD changes. Fluorescence of the G4 light up probe ThT was twice as high in the dsG4 sample as in the dsCntr sample (Supplementary Fig. S4B). The electrophoretic mobility of dsCntr was consistent with that of the 41-mer duplex, whereas dsG4 showed an additional PAGE band of reduced mobility, supposedly indicative of the correctly folded structure (Fig. 3A). The band ratio was close to 1:1, suggesting ∼50% G4 folding, in agreement with previous NMR-based estimates for an analogous construct containing the model G4 sequence (G_3_T) _3_G_3_ instead of the SE G4 [48]. Importantly, PDS increased the G4:duplex band ratio to nearly 2:1, whereas dsCntr showed no PDS-induced PAGE mobility changes. Taken together, these data suggest that the SE G4 is at least partially folded in dsG4 and probably in G4-Widom.

For nucleosome assembly assays, we selected H3K18ac as the acetylated component of the histone octamer (ac-HO), because it showed the highest affinity for BRD4 among all H3/H4 variants with single Kac residues in the N-terminus in the previously reported spot blot assays [43]. The nucleosomes assembled on both G4-Widom and Cntr-Widom DNA showed a PAGE mobility close to that of a 700 bp DNA marker (Fig. 3B), in agreement with previous data [48]. The G4-Widom nucleosome band intensity was lower than that of Cntr-Widom, with nucleosome:free DNA ratios of ∼0.4:1 and 0.5:1, respectively (day 1). After 3 days of sample incubation in the NAB, the ratios decreased to 0.03:1 (G4-Widom) and 0.15:1 (Cntr-Widom). By day 7, the nucleosome was completely disassembled in the G4-Widom sample but not in the Cnr-Widom sample (Fig. 3B). In conclusion, the SE G4 destabilized the acetylated HO at the adjacent nucleosome-positioning site in the minimal chromatin model.

Next, to verify the possibility of BRD4 maintenance at nucleosome-free G4 sites, we analyzed the affinity of BRD4 for isolated SE G4 and dsG4. Alternative non-canonical DNA structures, namely a stable intercalated motif (iM) [83] and a hairpin (ds26) [84], as well as B-DNA dsCntr were used as negative controls. Sequences of all ODNs are provided in Supplemenary Table S3. The Kac-containing fragment of H3K18ac, namely the 20-mer peptide S10-S28 (ac-peptide) was used as a positive control. The analysis was performed using surface plasmon resonance (SPR)-based and microscale thermophoresis (MST)-based methods, which are sensitive to protein/complex size and solvation shell, respectively. The presence of the large disordered C-terminus in BRD4 had a negative impact on the signal-to-noise ratio. Therefore, we used a partial recombinant protein, namely the N-terminal fragment encompassing tandem BDs (E49–E460), which account for BRD4 positioning on chromatin. This partial protein had an N-terminal 8-His tag, which enabled its labeling with RED-NTA for MST-binding assays (Supplementary Fig. S5A) or immobilization on a Ni^2+^-activated NTA-Au chip for SPR-binding assays (Fig. 3C).

The SPR assays revealed moderate affinity of the control ac-peptide for BRD4 (*K*_d_ = 3 ± 2 μM), comparable to that reported for other acetylated peptides from H3/H4 N-terminal regions [43], and a slightly lower affinity of SE G4 and dsG4 (*K*_d_ = 5 ± 3 μM). The MST assays gave slightly higher *K*_d_ values (4 ± 3 μM and 10 ± 1 μM) but agreed qualitatively with SPR. The NMR experiments independently confirmed the aforementioned *K*_d_ value for SE G4 (6 ± 1 μM) (Supplementary Fig. S5B). None of the control oligonucleotides (dsCntr, hairpin ds26, and iM) showed significant affinity for BRD4. Interestingly, the binding interactions between dsG4 and BRD4 were lost in the presence of the G4 ligand PDS (4 eq) but not in the presence of the known competitor of acetylated peptides JQ1 (Fig. 3C). We hypothesized that the G4 binding mode might be different from that of ac-peptide and investigated this *in silico*.

### SE G4 interacts with BRD4 BDs but does not enter the acetyl-K binding pocket *in silico*

**Figure 4.**
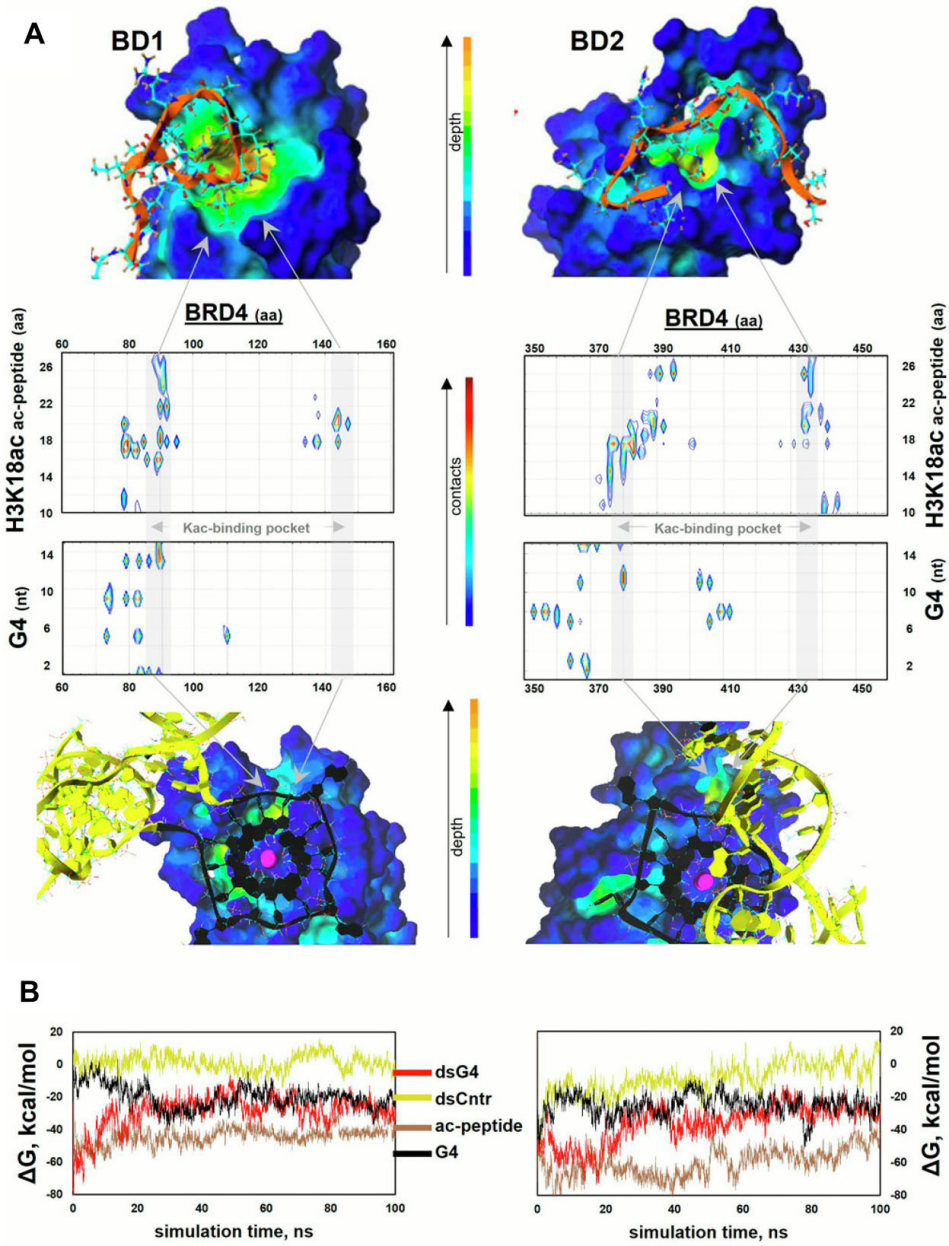
*In silico* analysis of BRD4 interactions with the acetylated histone tail and the SE G4. (**A**) Complexes of BRD4 BD1 (left) and BD2 (right) with the acetylated peptide (ac-peptide) from H3K18ac (top) and the SE G4 (bottom): MD simulation results (final snapshots) and contact maps. The complex models were obtained by docking ac-peptide or dsG4 to BD1/2, followed by MD simulations. Coloring scheme: brown, peptide backbone; black, G4; yellow, duplex flanks and the G4-opposing C-rich fragment. The contact maps were obtained based on the MD trajectories and illustrate the percentage of snapshots with contacts between the respective residues. Because the contact frequency between the C-rich DNA fragment and the duplex flanks was minor, the contact maps are shown for the G4 only, and the nucleotide residues are numbered accordingly (5′→3′). The amino acid numeration in ac-peptide and in BD1/2 agree with those in H3K18ac and full-size BRD4, respectively. (**B**) Free energies of BD1/2 binding with the ac-peptide, G4 without flanks, dsG4, and dsCntr. The energy plots were smoothed using the moving average method (span = 5).

Molecular modeling was used to elucidate the likely binding modes of the SE G4/dsG4 with BRD4 BDs and to compare them with those of ac-peptide. The G4 model was constructed in accordance with the recent studies [76], which supported its full parallel 3-quartet topology, consistent with our CD data (Supplementary Fig. S4A). Ac-peptide (H3K18ac fragment), G4, dsG4, and dsCntr were docked to BRD4 BD1 and BD2 (PDB: 2UVW and 6U6L). The resulting complexes were ranked based on the scoring function and additionally screened for consistency with the canonical binding mode in the case of the peptide. This canonical mode involves the positioning of the Kac residue within the hydrophobic pocket formed by interhelical BD loops [43]. In BD1, the pocket is framed by W81, P82, V87, L92, and I146 residues, while in BD2 it is framed by W374, P375, F376, V380, Y432, and V439 residues (Fig. 4A).

The stability of BRD4 complexes with ac-peptide, G4, dsG4, or dsCntr obtained from docking was verified by molecular dynamics (MD) simulations (Fig. 4A and Supplementary Figs S6 –S10). The evolution of the overall binding energy components is shown in Fig. 4B and Supplementary Fig. S11, respectively. The evolution of ac-peptide/dsG4/dsCntr contacts with BD1/BD2 is presented in Supplementary Fig. S12. The contributions of individual residues are detailed in Supplementary Table S4 and summarized in Fig. 4A as contact maps. The evolution of H-bonding is shown in Supplementary Fig. S12. The donor/acceptor groups are specified in Supplementary Table S5.

Ac-peptide exhibited a strong interaction with the hydrophobic pocket boundaries through K18ac and proximal residues (Fig. 4A and Supplementary Fig. S6), including R17 and L20 (Supplementary Table S4). Both K18ac and L20 contributed to pocket binding mainly through van der Waals interactions (Supplementary Table S4). Residues distant from K18ac formed only sporadic, short-lived contacts with the protein surface, except for the stable H-bonds between R26 and the BD2 surface (Supplementary Table S5). The K18ac residue formed H-bonds with intra-pocket residues N140 (BD1) or D381 (BD2) (Supplementary Table S5). The resulting complexes were stable throughout the simulation (Fig. 4B and Supplementary Fig. S11), with average MMGBSA energy values of ∼ −44 kcal/mol (BD1) and −60 kcal/mol (BD2).

The dsG4-binding sites in both BD1 and BD2 were distinct from those of ac-peptide, particularly in the case of BD2 (Fig. 4A and Supplementary Fig. S7). Most importantly, no dsG4 residues were positioned within the hydrophobic pocket. Instead, the G4 contacted the pocket-surrounding hydrophobic surface through its 5′-terminal quartet (G^1^G^5^G^9^G^13^) in BD1 or the 3′-terminal quartet (G^3^G^7^G^11^G^15^) and the central loop in BD2 (Supplementary Table S4). The potential overlap between BRD4-bound dsG4 and the BRD4 inhibitor JQ1 was minor in the case of BD1 and zero in the case of BD2 (Supplementary Fig. S8). The BRD4–dsG4 complexes were stable (Fig. 4B and Supplementary Fig. S11), with average MMGBSA energy values of ∼ −27 kcal/mol (BD1) and −32 kcal/mol (BD2). The increased stability of the complex with BD2 was mainly due to efficient van der Waals interactions (Supplementary Table S4), including pi–pi stacking between Phe365 and the G-quartet. The G4 flanks made a minor contribution, mainly through H-bonding of their backbone (Supplementary Table S5). Consistently, the isolated SE G4 showed BRD4 binding mode and energy similar to those of dsG4 (Fig. 4B and Supplementary Fig. S9). The control duplex dsCntr initially contacted a substantial surface of BRD4 through its minor and major grooves and formed multiple H-bonds but most of them were lost during the simulation (Supplementary Fig. S12). The MMGBSA energy was positive for BD1 and approximately equal to −8 kcal/mol for BD2 (Fig. 4B).

In summary, *in silico* studies suggest that dsG4 binds to BRD4 BD2 through the exposed aromatic surfaces of G residues from the terminal quartet outside the histone tail-binding pocket. This explains the strong inhibitory effect of the terminal quartet targeting ligand PDS and the minor effect of the histone competitor JQ1 on BRD4–G4 interactions in SPR assays (Fig. 3C). The *in silico* data also indicate that affinity of dsG4 is close to that of the isolated SE G4 and inferior to that of ac-peptide but vastly superior to that of dsCntr, which agrees qualitatively with SPR data (Fig. 3C).

### Similar to acetylated nucleosomes, SE G4 facilitates phase separation in BRD4 solutions

According to recent simulations, a combination of BRD4–ac-HO and BRD4–BRD4 interactions underlies the nucleation of BRD4 condensation [85]. The former interactions involve tandem BDs, whereas the latter involve IDRs, in particular CTD. We hypothesized that a minimal chromatin model with two acetylated H3K18ac histones might be sufficient to induce condensate nucleation *in vitro*, albeit at a higher critical concentration. Given the affinity of SE G4 for BRD4 demonstrated *in silico* and *in vitro*, we questioned whether this G4 could also affect phase separation in BRD4 solutions. To monitor phase separation by fluorescence microscopy, a mixture of RED-labeled (15%) and unlabeled full-length BRD4 protein with the LLPS-promoting C-terminus [86] was used. To mimic molecular crowding, the BRD4 solutions were supplemented with 20% PEG-400 prior to incubation with the G4, control DNA, H3K18ac, or the stable/unstable nucleosomes. The PEG fraction was equal to that used in most LLPS assays [87] and comparable to the intracellular concentration of macromolecules (up to 30%) [88]. An alternative option was to use a low fraction (2%–4%) of a bulky crowding agent, such as PEG-6000 [89]. However, as with an excessive concentration, the high molecular weight of the crowder may lead to aberrant protein dynamics [88].

**Figure 5.**
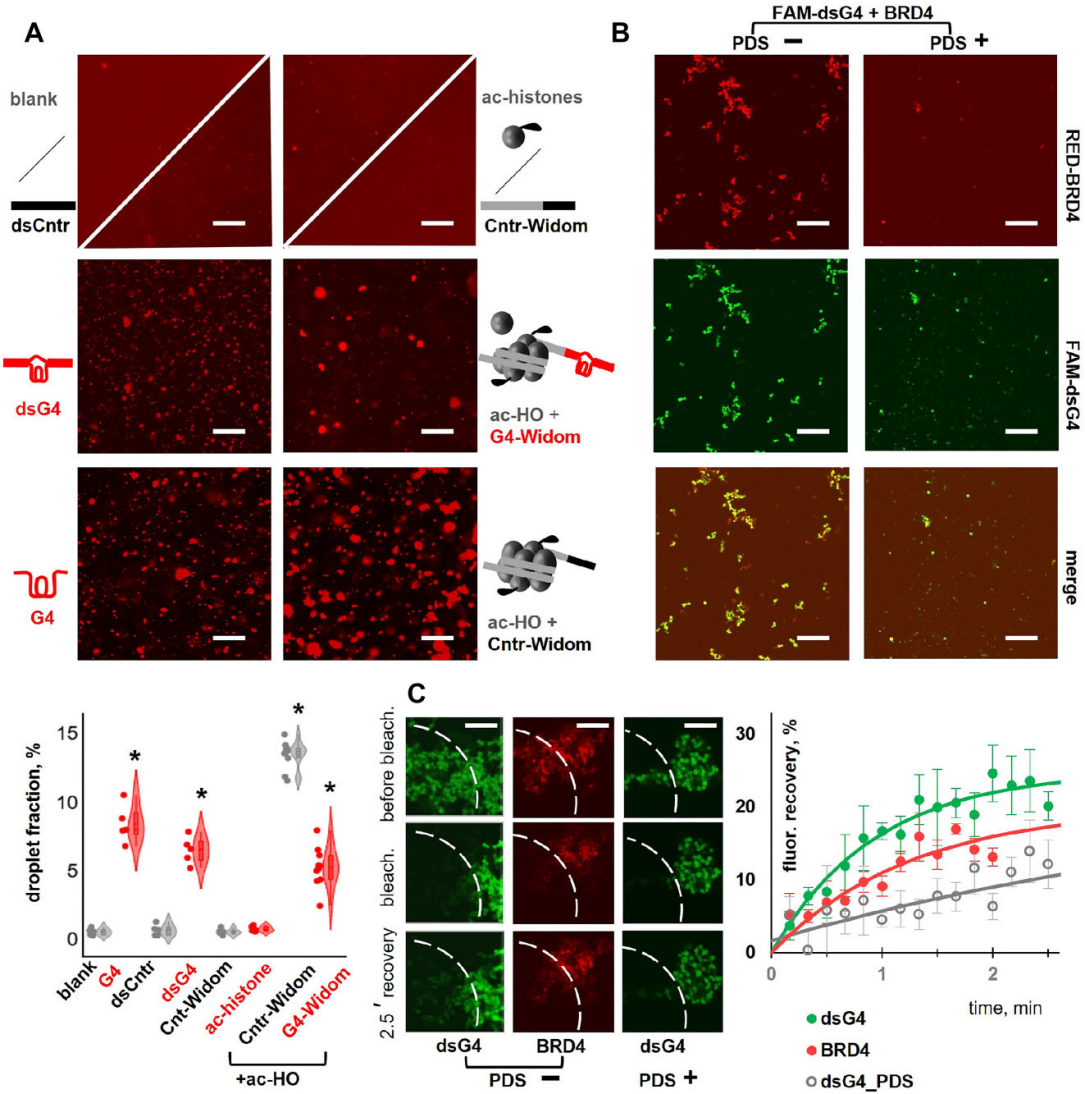
Effects of the G4 on phase separation in BRD4 solutions. (**A**) Fluorescent microscopy images of BRD4 mixtures with G4/control DNA, acetylated histones, or nucleosomes. BRD4 concentration: 4 μM (15% RED-labeled). Other components: 0/4 μM dsCntr (top, left); 4 μM dsG4 (middle, left); 4 μM G4 (bottom, left); 300 nM H3K18ac/150 nM Cntr-Widom (top, right); unstable nucleosome: 150 nM G4-Widom and 300 nM acetylated histone octamer (middle, right); stable nucleosome: 150 nM Cntr-Widom and 300 nM acetylated histone octamer (bottom, right). Buffer: 1× PBS (pH 7.4) supplemented with 20% PEG-400. Samples with G4-Widom/Cntr-Widom also contained 10 mM Tris–HCl (pH 8.0) and 125 mM NaCl from the NAB; scale bar: 50 μm. Violin plots summarize microscopy-based evaluation of droplet fraction in each sample. *Significant increase as compared to the blank sample (BRD4 only), *P* < 0.0001, one-way ANOVA with Dunnett’s post-hoc test. (**B**) Confocal microscopy images of BRD4 mixtures with dsG4 on mPEG-modified glass in the presence and absence of PDS. Conditions: 4 μM BRD4 (15% RED-labeled), 4 μM dsG4 (the G4-containing strand is FAM-labeled), and 0/20 μM PDS in 10 mM Tris–HCl buffer (pH 8.0) supplemented with 125 mM NaCl and 20% PEG-400; scale bar: 50 μm. (**C**) FRAP in BRD4-dsG4 gel-like structures: representative snapshots (bleached zones are marked by dashed lines) and kinetic curves (average of three measurements); scale bar: 10 μm.

In the absence of DNA and histones, no signs of LLPS were observed in BRD4 solutions of at micromolar concentrations (up to 4 μM), except for a few proto-condensates/aggregates of (sub)micron diameter. The addition of stable and unstable nucleosomes (ac-HO assembled on Cntr-Widom and C4-Widom) caused strong and moderate LLPS, respectively, while DNA-free acetylated histones or histone-free G4-free DNA had no significant effects. Hairpin DNA and dsCntr also had no effect, whereas G4 and dsG4 were rather efficient LLPS facilitators, inferior to the stable nucleosome but comparable to the unstable one (Fig. 5A and Supplementary Fig. S13).

Similar to the nucleosomes, G4, and dsG4 induced the formation of BRD4-rich droplets up to several micrometers in diameter. Visual screening confirmed their floating and gradual coalescence. The partitioning coefficient could not be estimated with reasonable accuracy due to rapid background bleaching. Therefore, the volume fraction of the droplets was chosen as a representative parameter. Consistent with the presumed liquid state, the droplets showed a circularity of 80 ± 10%, so we assumed them to be spheres and estimated their volume fraction based on the apparent radii, which in turn were calculated using the projection area in the microscopy images. The results are summarized as a violin plot in Fig. 5A and illustrate significant (*P* < 0.0001, one-way ANOVA with Dunnett’s post-hoc test) enrichment of the droplet fraction in G4 (8 ± 1%), dsG4 (6 ± 1%), G4-Widom (5 ± 1%), and Cntr-Widom (13 ± 1%) mixtures with BRD4 compared to free BRD4 (<1%).

The LLPS-promoting effect of the stable acetylated nucleosome was consistent with the consensus SE model [41], whereas the effect of the unstable nucleosome, which we attribute to the G4 tail, has not been previously observed. Therefore, we further examined both samples by AFM to additionally verify biocondensate formation (Supplementary Fig. S14). In the absence of BDR4, the nucleosomes assembled on Cntr-Widom were visualized as 2.2 ± 0.3 nm granules (ac-HOs) with protruding dsDNA tails and showed no aggregation (Supplementary Fig. S14A and B). Consistent with the PAGE data (Fig. 3B), ac-HOs on G4-Widom tended to be partially or completely disassembled (Supplementary Fig. S14A). The addition of BRD4, which was visualized as 2.6 ± 0.3 nm granules (Supplementary Fig. S14A and B), induced the formation of complexes and condensate precursors of sub-micron size in both samples (Supplementary Fig. S14C). Some of them had irregular shapes, arguing for possible gelation on the AFM support.

AFM was also used to compare BRD4 mixtures with dsG4 and dsCntr. The former was found to contain small condensate precursors along with free DNA. The distribution of the DNA between the condensates and the bulk solution was assessed directly from the AFM images. In the case of the nucleosomes, such an analysis was complicated by strand entanglement and overlap. The apparent fraction of dsG4 in condensates (13 ± 5%) was significantly higher (*P* < 0.05, two-tailed *t*-test) than that of dsCntr (5 ± 2%), in qualitative agreement with the fluorescence microscopy data (Supplementary Fig. S14D and Fig. 5A).

The colocalization of BRD4 with dsG4 in condensates could not be analyzed with reasonable accuracy by AFM. Therefore, we obtained FAM-labeled dsG4 and investigated its mixture with RED-labeled BRD4 by confocal fluorescence microscopy. These studies required condensate fixation on microscopy plates. We used glass plates coated with mPEG and passivated with bovine serum albumin (BSA), which ensured optimal fixation of chromatin condensates in previous studies [67, 68]. The dense phases visualized in both RED–BRD4 and FAM–dsG4 fluorescence channels were colocalized and had irregular shapes (Fig. 5B), suggesting partial solidification or gelation on the glass surface. Fluorescence recovery after photobleaching (FRAP) was used to test their liquid versus solid state (Fig. 5C). Both BRD4 (RED) and dsG4 (FAM) fluorescence signals showed only partial recovery (below 25%) with rate constant values of 0.8 ± 0.1 and 1.1 ± 0.1 min^−1^, respectively, suggesting limited but detectable BRD4/dsG4 diffusion and exchange between the gel-like condensates and the bulk solution.

To summarize this part, we showed that SE G4, but not B-DNA, facilitates BRD4 phase separation at a low micromolar concentration, close to that of the G4–BRD4 binding constant. As the BRD4–G4 binding interactions are ligand sensitive (Fig. 3C), we next tested the effects of the ligands on phase separation.

### Phase separation in BRD4–G4 solutions is sensitive to G4 ligands

The effect of the G4 and BRD4 ligands on phase separation in BRD4 mixtures with G4/dsG4 was monitored by fluorescence microscopy. The water-insoluble/poorly soluble ligands were added with 0.5% DMSO admixtures and compared to a 0.5% DMSO containing blank. DMSO is known to alter LLPS by modulating H-bonding [90], and in the case of BRD4 it reduced the droplet fraction almost 2-fold (Supplementary Figs S13 and S15A and B). The ac-histone competitor JQ1 [91], caused a 3-fold decrease in the average droplet fraction in the BRD4 mixture with the acetylated nucleosome (Supplementary Figs S12 and S15A and B). However, it had no significant effect on the BRD4 mixture with the G4 (Supplementary Fig. S15A and B), supporting the difference between the BRD4–histone and BRD4–G4 binding modes.

Phase separation in the BRD4–G4 mixture was almost completely abolished by a universal inhibitor of hydrophobicity-dependent LLPS, 1,6-hexanediole (HD) [92, 93], supporting the contribution of hydrophobic interactions to condensate formation. Significant inhibition of the separation was also observed for two G4 ligands: PDS, which is commonly used as a reference G4-targeting antiproliferative agent [82], and SOP1812, the G4-targeting antiproliferative drug that has shown remarkable potency in several *in vivo* cancer models and is currently in clinical trials [94, 95]. We attribute these inhibitory effects to interference of the ligands with G4-BRD4 binding, presumably by shielding external G-quartets from BRD4 BDs. The control antiproliferative agent, the antimetabolite 5-fluorouracil (5FU), which does not bind BRD4 or G4s, induced no changes (Supplementary Fig. S15).

Interestingly, along with reducing the droplet fraction, PDS and SOP1812 facilitated condensate transition to irregularly shaped gel-like or solid objects (Supplementary Fig. S15): the circularity decreased from 0.8 ± 0.1 to 0.6 ± 0.1. This effect might be due to the occasional formation of triple complexes (BRD4–G4–ligand) with reduced solubility or altered interaction with the glass surface. Both effects were particularly pronounced in the case of PDS, so we additionally verified them using the BRD4–dsG4 mixture.

Confocal microscopy revealed a PDS-induced reduction in the fraction of condensate/gel-like objects and the colocalization of BRD4 (RED) and dsG4 (FAM) signals in the remaining irregularly shaped objects (Fig. 5B). FRAP confirmed a reduced mobility of dsG4 in the presence of PDS with a recovery rate of 0.2 ± 0.1 min^−1^ (5 times slower than in the absence of PDS) (Fig. 5C), while BRD4 mobility could not be analyzed with reasonable accuracy. AFM revealed a negligible fraction of condensate precursors in the presence of PDS (Supplementary Fig. S14D and E), consistent with the fluorescence microscopy data.

In conclusion, phase separation in BRD4–G4 solutions was insensitive to the non-G4-targeting control drug 5FU and the histone competitor JQ1 but sensitive to the G4 ligands PDS and SOP1812. Both these ligands inhibited LLPS and induced occasional gelation. The latter effect may be related to sorption artifacts, which are a common limitation of *in vitro* LLPS assays [67]. Another limitation of our assays is the lack of additional components that co-separate with BRD4, including the Mediator complex, enhancer RNA, etc. In this regard, our findings may not be directly indicative of intracellular phase transitions, but rather encourage their further investigation.

In cells, both dissolution and gelation of BRD4 condensates have previously been observed upon BRD4 imbalance or treatment with JQ1, and both changes were associated with transcriptional repression [96–98]. If G4s contribute to phase separation upon BRD4 accumulation at SEs, the resulting condensates might also be responsive to G4 ligands, suggesting a possible inhibition of the SE–promoter communication and the repression of the respective genes. Therefore, our next goal was to test whether the antiproliferative effects of PDS and SOP1812 are at least partially SE-related.

### Genes activated by conserved G4-rich SEs show increased sensitivity to G4 ligands

The effects of PDS and other G4 ligands on gene expression have previously been studied at the whole genome level [99–101]. The results do not allow to distinguish promoter-related effects from those related to SEs or common enhancers from the same topologically associated domain (TAD). However, the phase separation model of transcriptional control [102] suggests an increased sensitivity of SEs to any perturbation compared to other regulatory elements. Recently, a positive correlation between Pol II-mediated contacts with distal regulatory elements and PDS-induced transcriptional repression has been demonstrated in HEP-G2 cell line, supporting PDS interference with the enhancer–promoter communication [31]. Whether this effect is cell line-specific and valid for BRD4-mediated contacts awaits clarification. We aimed to partially fill these gaps. Instead of performing another integrative transcriptomic analysis of PDS-treated cells, we focused on several genes within well-defined TADs with confirmed G4-rich SEs in K-562/HEK-293 cell lines and compared them with control genes from SE-free TADs.

**Figure 6.**
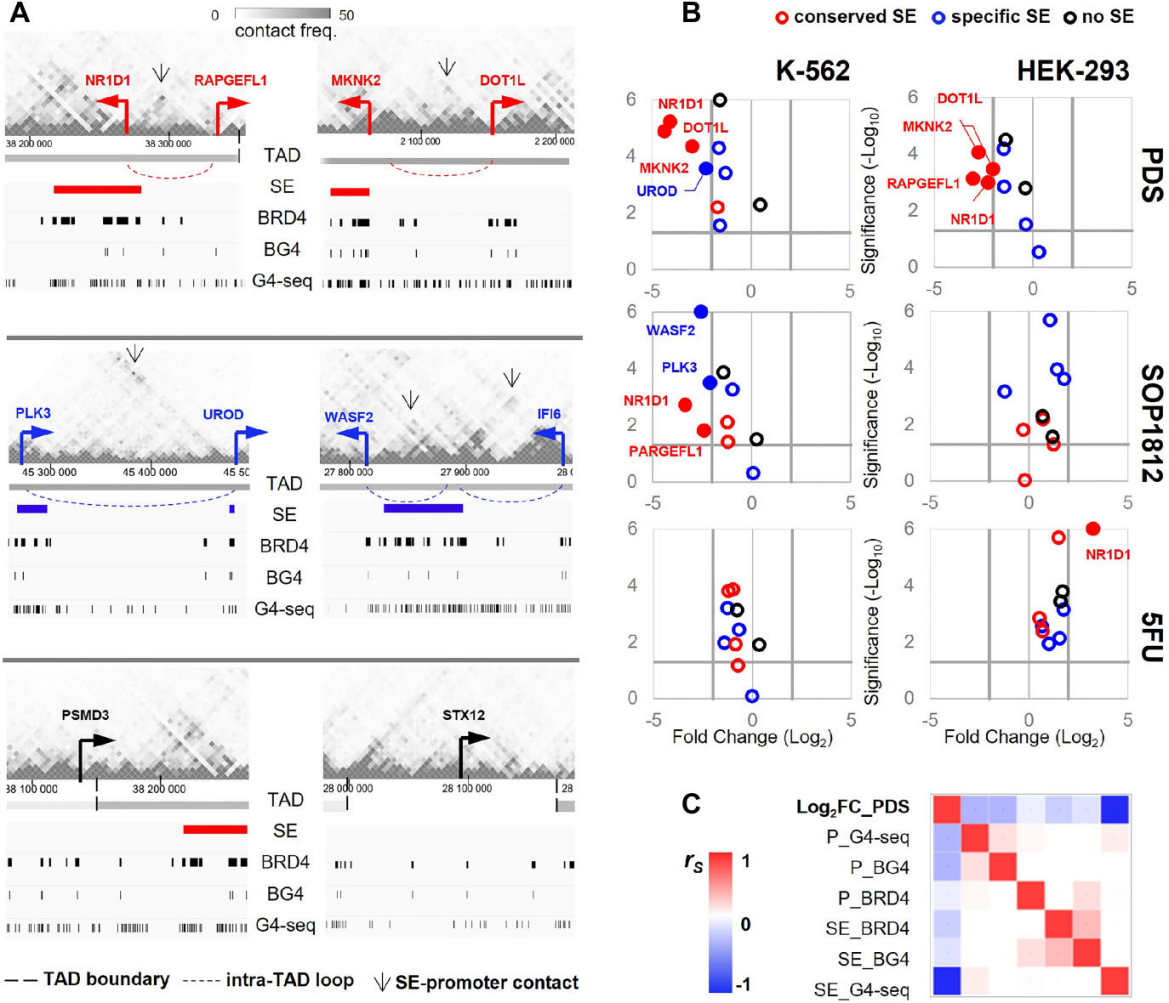
Effects of the G4 ligands on the expression of genes contacting G4-rich SEs. (**A**) Selection of genes dependent on conserved (active in both cell lines; top pannel) or specific (active/poised in K-562; middle pannel) G4-rich SEs and control SE-independent genes (bottom pannel). For each gene set, 3D context, nearest SEs, BRD4 occupancy and G4-richness (G4-seq and BG4 narrow peaks) in K-562 cells are shown. SE-dependent genes are those that overlap SEs or participate in long-range interactions with same-TAD SEs, as indicated by the Hi-C data (above-average off-diagonal contact frequency). SE-independent genes are those found in the SE-free TAD/inter-TAD region. (**B**) Regulation of the selected genes by the G4 ligands PDS/SOP1812 or the control compound 5FU in K-562 (left) and HEK-293 (right). The volcano plots illustrate the stringent analysis (Log2FC threshold = 2, *P*-value threshold = 0.05). (**C**) Spearman correlation between gene regulation by PDS (Log2FC_PDS) and the selected SE/promoter (P) features, namely the highest G4-seq/BG4/BRD4 peak score in the promoter region and the G4-seq/BG4/BRD4 coverage in the related SE.

From the set of high to medium rank G4-rich SEs (top ten SEs in Supplementary Table S1), we selected two conserved SEs (SE_01_03900094 and SE_01_03900154, active in both K-562 and HEK-293 cell lines) and two cell type-specific SEs (SE_01_03900056, active in K-562 and repressed in HEK-293, and SE_01_03900348, poised in K-562 and repressed in HEK-293). We then searched for genes regulated by these SEs, i.e. those located in the same TAD and showing an above-average frequency of promoter contacts with the SE, according to reported Hi-C-based maps of the K-562 3D genome [60]. All of these genes contained BG4 peaks in their promoters, consistent with previous studies of G4 involvement in the promoter–SE communication [72, 103]. Control genes under BG4-positive promoters that do not form any long-range contacts with regulatory sites occupied by BRD4 clusters were selected from SE-free TADs. The BG4-free housekeeping genes *RPLP0* and *RPS18* were used as a reference. The ten selected genes are listed in Supplementary Table S6, and their 3D contacts are shown in Fig. 6A.

Using RT-PCR, we compared the expression of the selected genes in K-562/HEK-293 cells treated with PDS, SOP1812, blank, or the control antiproliferative agent 5FU (Fig. 6B) at concentrations corresponding to the IC30 values (Supplementary Fig. S16). With a single exception (*NR1D1* upregulation in 5FU-treated HEK-293 cells), stringent analysis (log_2_FC threshold = 2) revealed no effects of the control ligand 5FU on the genes of interest. PDS had no significant effects on control genes and those contacting specific SEs in either cell line, with a single exception (*UROD* downregulation in K-562). However, it downregulated all genes contacting the conservative SEs, except *RAPGEFL1* in K-562. SOP1812 had no significant effects in HEK-293, whereas in K-562 it downregulated half of the genes contacting specific/conserved SEs. To summarize, both G4 ligands, but not 5FU, downregulated at least some of the SE-dependent genes, and the downregulation was more pronounced in K-562.

These results may be specific to the selected TADs and do not prove the critical role of G4-rich SEs in cell responses to the G4 ligands. However, they clearly show that promoter G4s are not the only key players. To further address this issue, we next compared BRD4/BG4/G4-seq peak scores in the promoters of all analyzed genes and found no strong correlations with the ligand-induced expression changes (the Spearman coefficient module was <0.4). Similar results were obtained for an additional set of genes that could not be reliably related to a particular SE or classified as SE-free based on the available contact maps (Supplementary Table S6). Analysis of their PDS responses in combination with the main gene set or separately also revealed no strong correlations with BG4/G4-seq peaks. The positive correlation between G4-seq and BG4 was also weak or absent in both promoters and SEs, illustrating that only few potential G4s are folded in the absence of a stabilizing ligand. However, SE G4-seq peak coverage correlated strongly with the extent of PDS-induced changes (Fig. 5C). The Spearman coefficient (*r*_s_) was −0.8 for the main gene set (−0.7 after excluding the control genes), indicating efficient downregulation of genes contacting G4-seq-rich SEs (two-tailed *P* < 0.05).

Thus, we have shown that genes dependent on G4-rich SEs could be targeted by the G4 ligands, such as PDS or SOP1812, to facilitate their downregulation. One possible mechanism of ligand activity is the interference with BRD4 accumulation at SEs, probably in combination with the interference with TF accumulation at promoters [23].

## Discussion

Our study demonstrated that G4-richness is associated with nucleosome depletion in SEs (Fig. 1C). This result appears to be inconsistent with the findings of Young’s group, who compared G4-free SEs with all G4-containing SEs and found no significant difference in chromatin accessibility [29]. However, Young *et al.* noted that the majority of SEs contained at least one G4, prompting us to consider normalized G4 counts rather than absolute values. Instead of performing an integrative SE analysis, we compared G4-free SEs to a subset of G4-containing SEs. This subset included those with the highest BG4 coverage, representing 25% of total, and showed a significant increase in the DHS coverage (*P* < 0.05). Thus, our findings do not contradict the previous report but rather complement it. Furthermore, they align with the previously reported increased DHS coverage in G4-containing common enhancers compared to G4-free enhancers [29] and in G4-containing promoters compared to G4-free promoters [49]. They are also consistent with the enhanced eviction of acetylated nucleosomes near G4s in the minimal chromatin model evidenced by PAGE (Fig. 3B). The eviction may result from distortions in duplex geometry around G4s and/or G4 interference with HO sliding; however, additional studies are required to elucidate the mechanism in greater detail.

Previous studies have demonstrated increased loading of DNA-binding architectural chromatin proteins (e.g. CTCF) and their partners (e.g. RAD21) at G4-containing SEs [29]. Our findings indicate that the histone mark reader/writer BRD4 is also slightly enriched at G4-rich SEs (Fig. 1C). Its intrinsic HAT activity may be at least partly responsible for nucleosome eviction at SEs, because in addition to lysine residues in histone tails (H3K18, H3K27, etc.), it can acetylate H3K122, positioned within the core nucleosome particle and involved in DNA binding [47]. Consequently, nucleosome depletion at G4-rich SEs may result from a combined effect of nucleosome acetylation by BRD4 and exclusion by G4s. Importantly, BRD4 is supposed to remain weakly bound to chromatin even after nucleosome eviction to maintain SE-promoter communication and gene expression. Consistently, ChiP-seq data support the colocalization of BRD4 peaks with nucleosome-free G4 sites flanked by H3K27ac peaks in K-562 G4-rich SEs. The colocalization is partly attributable to BRD4 binding to G4 DNA via BDs. Strong binding is unfavorable, as it would prevent BRD4 from co-separating with the Mediator. Indeed, the reported *K*_d_ values for BRD4 complexes with acetylated histone tails range vary from low to medium micromolar values [43], and the *K*_d_ values of BRD4-ac-peptide and BRD4-dsG4 obtained herein were in the same range (Fig. 3C and Supplementary Fig. S5A). Notably, the *in silico* data (Fig. 4) argue that the G4 interacts with BRD4 BDs through its external quartets, which can be targeted by ligands like PDS or SOP1812, outside the histone-recognizing Kac-binding pockets, targeted by histone competitors like JQ1 (Supplementary Fig. S8). Consistently, G4-BRD4 interactions were disrupted by PDS and SOP1812 but not by JQ1 in the SPR assays.

It can be concluded that G4s enhance SE multivalency and may partially compensate for nucleosome eviction with respect to BRD4 maintenance. In addition to the semi-specific interactions with BRD4 BDs, G4s may participate in non-specific interactions with the disordered BRD4 C-terminus. In contrast to B-DNA, which is only accessible for electrostatic interactions, G4s have solvent-exposed aromatic planes (external G-tetrads) available for cation–pi or pi–pi interactions [104], which are common in biomolecular condensates [105, 106]. Consistently, we showed that G4s, but not B-DNA, facilitate the formation of PDS/SOP1812-sensitive biocondensates/gels in BRD4 solutions. Previous studies have demonstrated that G4s also facilitate LLPS in linker histone (H1) solutions [107], RecQ4 helicase solutions [108], and SERBP1-containing protocell-imitating vesicles [109].

In cells, G4s are presumed to promote TRF1/2-driven LLPS at telomeres [110] and may be at play in nucleoli scaffolding [111]. Furthermore, G4s colocalize with the clusters of 5S-phosphorylated (paused) Pol II, a hallmark of activated promoters [112]. This supports their presence in transcription initiation condensates (hubs) [113]. Such condensates are distinct from those scaffolded by 2S-phosphorylated/hyperphosphorylated (released) Pol II upon transcription elongation [114, 115]. Although Pol II contributes to LLPS through its IDR in both types of condensates [116], the promoter–enhancer encompassing hubs are predominantly scaffolded by the Mediator complex and BRD4 [41, 114]. The contribution of BRD4 has recently been confirmed using the PROTAC-based approach [117] and the CRISPR–Cas9 optogenetics-based approach [118]. The BRD4 C-terminus plays a pivotal role in phase separation both *in vitro* and *in vivo* [97]. Interestingly, its deletion does not abolish BRD4 clustering but rather converts condensates to gel-like structures (irregularly shaped puncta) in mice and human cells [97]. This phenomenon resembles the herein observed shift toward gel upon the addition of the G4 stabilizer PDS to BRD4–G4 mixtures (Fig. 5B).

Modulation of BDR4-driven LLPS is a promising approach to repressing gene expression. The non-specific LLPS inhibitor HD, used as a positive control in our *in vitro* assays (Supplementary Fig. S15), reportedly disrupts BRD4/Mediator condensates in cells [41] but has no potential therapeutic applications due to high toxicity. The first-generation BET*i* JQ1 had a less profound effect, yet still caused BRD4 puncta dispersion in cells [89] and in BRD4 mixtures with the acetylated nucleosomes (Supplementary Fig. S13). In contrast, its effect on BRD4–G4 condensates was minimal, consistent with the positioning of the G4 outside the acetyl-lysine binding pocket of BRD4 BDs (Supplementary Fig. S8). Recently, JQ1 has been shown to ultimately enhance intracellular LLPS [98], presumably due to the compensatory BRD4 overexpression, which limits its applicability in both basic studies and cancer therapy. The development of next-generation BET*i* [119] and other pan-condensate modulators [98] may provide new anticancer candidates [120]. However, they are expected to disrupt all SE–promoter contacts. We argue that G4 ligands may target only a subset of SEs, suggesting the potential for local transcriptional repression. As illustrated in Fig. 6C, gene responses to the G4 ligand PDS, which inhibited BRD4–dsG4 separation *in vitro* (Fig. 5B), correlated with the G4 folding potential of gene-activating SEs in K-562. Importantly, the interference of the G4 ligands with the SE–promoter communication is likely to be multipath. For instance, in addition to inhibiting BRD4 separation, they may affect the recently proposed intermolecular (SE–promoter) G4 folding [72, 121]. Whether these roles of SE G4s and ligands are in concert requires further investigation.

In conclusion, we have shown that G4s enriched in human SEs exclude acetylated nucleosomes but bind to BRD4. This allows for the maintenance of SE multivalency and BRD4 occupancy. SE G4 DNA, but not B-DNA, facilitates phase separation in BRD4 solutions, and this effect is comparable to that of acetylated nucleosomes. In contrast to condensates assembled in BRD4–nucleosome mixtures, BRD4–G4 condensates are insensitive to the acetylated histone competitor but sensitive to the G4 ligands PDS and SOP1812. These findings suggest that the G4 ligands may affect SE function. The observed downregulation of genes activated by G4-rich SEs supports this thesis and implies that the SE-related effects might complement the promoter-related effects of G4 binders. The results of this study will hopefully encourage future studies of G4 ligands in combination with new BET*i* for selective regulation of SE-dependent genes.

## Supplementary Material

gkaf726_Supplemental_File

## Data Availability

All relevant data are available in the main manuscript and supplementary information.
