# Supplementary material for: G-quadruplexes as potential traps for superenhancer marker BRD4: ligand-sensitive binding and co-separation in vitro
Source: Nucleic Acids Res. 2025 Jul 31;53(14):gkaf726. doi: 10.1093/nar/gkaf726 (PMC12311796; doi:10.1093/nar/gkaf726)
Supplement: gkaf726_Supplemental_File [file gkaf726_supplemental_file.pdf]

## SUPPORTING INFORMATION

# G-QUADRUPLEXES AS POTENTIAL TRAPS FOR SUPERENHANCER MARKER BRD4: LIGAND-SENSITIVE BINDING AND CO-SEPARATION IN VITRO

Iuliia I. Pavlova, Olga M. Ivanova, Mikhail S. Iudin, Anastasiya V. Surdina, Nikolay A. Barinov, Margarita E. Bogomiakova, Sergey D. Oreshkov, Zakhar O, Shenkarev, Vjacheslav V. Severov[1], Dmitriy V. Klinov, Victoria O. Shender, Alexandra N. Bogomazova, Maria A. Lagarkova, Anna M. Varizhuk*, Vladimir B. Tsvetkov*

* To whom correspondence should be addressed:
Anna Varizhuk: Tel: +7 (499) 246-4409; Fax: +7 (499) 246-4409; Email: annavarizhuk@gmail.com
Vladimir Tsvetkov: Tel: +7 (499) 246-4409; Fax: +7 (499) 246-4409; Email: v.b.tsvetkov@gmail.com

Figure S1. G4 enrichment in SEs vs promoters.................... 2

Table S1. G4-rich SEs used for MEME analysis.................... 3

Table S2. SE fragments matching the top-scoring enriched G4 motif: FIMO results.................... 4

Figure S2. BG4 coverage active vs repressed SEs (a) or poised and active SEs (b).................... 5

Figure S3. G4-free and G4-rich SEs in K-562 and HEP-G2 cell lines: active and poised SEs.................... 6

Table S3. Sequences of all ODNs used in nucleosome assembly assays, BRD4-binding assays, and LLPS assays.................... 7

Figure S4. Verification of G4 folding in dsG4 and its affinity for BRD4 by CD and ThT-assay.................... 8

Figure S5. Verification of G4 folding in dsG4 and its affinity for BRD4 by MST-assay and NMR.................... 9

Text Box S1. Molecular modeling details.................... 10

Figure S6. Models of BRD4 complexes with its cognate binding partner – ac-peptide.................... 12

Figure S7. Models of BRD4 complexes with dsG4.................... 13

Figure S8. Positions of dsG4 and JQ1 on BRD4 BD1 (left) and BD2 (right).................... 14

Figure S9. Models of BRD4 complexes with the G4 (no flanks).................... 15

Figure S10. Models of BRD4 complexes with dsCntr.................... 16

Figure S11. Comparison of ac-peptide, G4, dsG4, and dsCntr binding with BRD4 BD1 (left) and BD2 (right): contributions to the binding energy.................... 17

Figure S12. Comparison of H3K18ac, SE-G4, and dsDNA binding with BRD4 BD1 (top) and BD2 (bottom): H-bonds and BD contacts.................... 18

Table S4. Detailed analysis of ac-peptide and dsG4 binding with BRD4: contributions of individual residues.................... 19

Table S5. Detailed analysis of ac-peptide and dsG4 binding with BRD4 BD1 (grey background) and BD2 (white background): H-bonding.................... 19

Figure S13. Phase separation in BRD4 mixtures with G4, control DNA and respective nucleosomes: large-field fluorescent microscopy images.................... 20

Figure S14. Phase separation in BRD4 mixtures with G4, control DNA and respective nucleosomes: atomic force microscopy (AFM) images.................... 21

Figure S15. Phase separation in BRD4-G4 mixtures: effects of BRD4/G4 ligands.................... 22

Table S6. Genes selected for the analysis of up/down regulation by PDS.................... 22

Figure S16. Cellular toxicity of the G4 ligands (PDS and SOP1812) and the control compound (5FU).................... 24

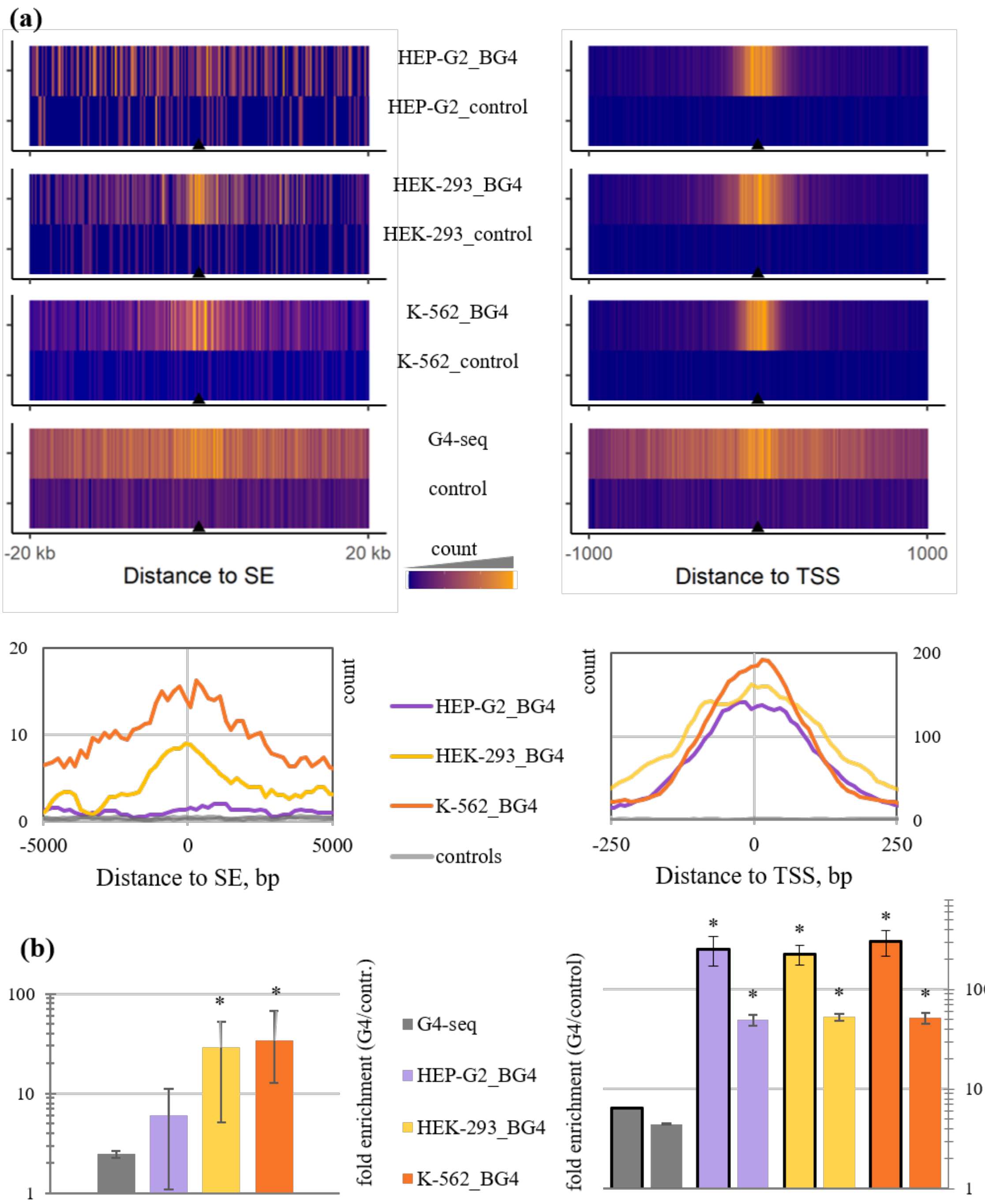


**Figure S1. G4 enrichment in SEs vs promoters.** (**a**) Distribution of potential (G4-seq) and confirmed (BG4) G4 sites relative to SEs and transcription start sites (TSS) in three cell lines. Each set of BG4 peaks (ChIP-seq/CUT&Tag data obtained using the BG4 antibody) was compared to a control set of randomly selected sites of equal size and average length, and their distribution was analyzed relative all HEP-G2/HEK-293/ K-562 SEs from SE db 2.0. In the case of G4-seq, pooled HEP-G2, HEK-293, and K-562 SE sets were used. All distribution profiles were smoothed using the moving average method (span = 5). (**b**) Summary of G4 enrichment in SEs (left) and promoters (right): permutation tests. The bar charts illustrate relative frequencies of G4s and control sites within SEs (left plot), general promoters (TSS ± 1000 bp, borderless bars, right plot) or core promoters (TSS ± 100 bp, black bar borders, right plot); data are presented as mean ± SD fold enrichment of G4s over control. * Significant difference between G4-seq and BG4 ($p < 0.05$, Mann-Whitney test).

**Table S1. G4-rich SEs used for MEME analysis.**

| SE type | SE ID in SEdb 2.0 (K-562) | chr | Start* | End* | rank | G4-seq count | G4-seq freq. | BG4 count | BG4 freq.** | present in HEK-293 |
|---|---|---|---|---|---|---|---|---|---|---|
| active | SE_01_03900005 | chr16 | 85556307 | 85656073 | 5 | 72 | 7.22 | 11 | 1.10 | + |
| | SE_01_03900048 | chr10 | 72409004 | 72517319 | 48 | 41 | 3.79 | 2 | 0.18 | - |
| | SE_01_03900056*** | chr1 | 27830278 | 27900086 | 56 | 57 | 8.17 | 3 | 0.43 | - |
| | SE_01_03900071 | chr3 | 73159466 | 73160498 | 71 | 1 | 9.69 | 1 | 9.69 | - |
| | SE_01_03900080 | chr19 | 45926050 | 45989734 | 80 | 26 | 4.08 | 10 | 1.57 | - |
| | SE_01_03900094*** | chr19 | 2034446 | 2062937 | 94 | 32 | 11.23 | 7 | 2.46 | + |
| | SE_01_03900154*** | chr17 | 38219164 | 38281051 | 154 | 51 | 8.24 | 3 | 0.48 | + |
| | SE_01_03900294 | chr19 | 18512233 | 18553632 | 294 | 53 | 12.80 | 4 | 0.97 | + |
| | SE_01_03900308 | chr1 | 154971359 | 154990749 | 308 | 36 | 18.57 | 3 | 1.55 | + |
| poised | SE_01_03900348*** | chr1 | 45476133 | 45480956 | 348 | 2 | 4.15 | 3 | 6.22 | - |
| | SE_01_03900374 | chr19 | 47728520 | 47733678 | 374 | 10 | 19.39 | 4 | 7.75 | + |
| | SE_01_03900423 | chr19 | 13206330 | 13215982 | 423 | 18 | 18.65 | 2 | 2.07 | + |
| | SE_01_03900471 | chr19 | 49630844 | 49665470 | 471 | 32 | 9.24 | 7 | 2.02 | - |
| | SE_01_03900550 | chr7 | 128466526 | 128473940 | 550 | 12 | 16.19 | 3 | 4.05 | - |
| | SE_01_03900600 | chr19 | 59065060 | 59072302 | 600 | 11 | 15.19 | 3 | 4.14 | - |
| | SE_01_03900613 | chr8 | 144988714 | 145031500 | 613 | 40 | 9.35 | 8 | 1.87 | + |
| | SE_01_03900633 | chr17 | 27050878 | 27056513 | 633 | 5 | 8.87 | 4 | 7.10 | - |
| | SE_01_03900648 | chr16 | 28833244 | 28837373 | 648 | 2 | 4.84 | 3 | 7.27 | - |
| SE type | SE ID in in SEdb 2.0 (HEK-293) | chr | Start* | End* | rank | G4-seq count | G4-seq freq. | BG4 (G4P) count | BG4 (G4P) freq.** | present in K-562 |
| active | SE_02_43600001 | chrX | 39653394 | 39875900 | 1 | 111 | 4.99 | 0 (2) | 0 (0.09) | - |
| | SE_02_43600005 | chr15 | 70381020 | 70520450 | 5 | 63 | 4.52 | 2 (5) | 0.14 (0.43) | - |
| | SE_02_43600011 | chr17 | 79358647 | 79405136 | 11 | 46 | 9.89 | 2 (13) | 0.43 (2.80) | - |
| | SE_02_43600012 | chrX | 39989857 | 40037874 | 12 | 34 | 7.08 | 1 (16) | 0.21 (3.33) | + |
| | SE_02_43600014 | chr9 | 139419483 | 139522273 | 14 | 85 | 8.27 | 1 (5) | 0.10 (0.49) | - |
| | SE_02_43600020 | chr13 | 100619662 | 100646569 | 20 | 21 | 7.80 | 3 (13) | 1.11 (4.83) | - |
| | SE_02_43600025 | chr18 | 46455559 | 46548567 | 25 | 58 | 6.24 | 0 (4) | 0 (0.43) | - |
| | SE_02_43600031 | chr15 | 96865843 | 96907297 | 31 | 37 | 8.93 | 0 (14) | 0 (3.38) | - |
| | SE_02_43600041 | chr19 | 13123274 | 13208085 | 41 | 59 | 6.96 | 0 (2) | 0 (0.24) | + |
| | SE_02_43600061 | chr17 | 77763199 | 77788320 | 61 | 30 | 11.94 | 2 (14) | 0.80 (5.57) | + |
| poised | SE_02_43600122 | chrX | 153233043 | 153240403 | 122 | 6 | 8.15 | 1 (9) | 1.36 (12.23) | + |
| | SE_02_43600172 | chr17 | 47071889 | 47077759 | 172 | 15 | 25.55 | 1 (3) | 1.70 (5.11) | + |
| | SE_02_43600231 | chr16 | 1818899 | 1824674 | 231 | 5 | 8.66 | 1 (6) | 1.73 (10.39) | - |
| | SE_02_43600298 | chr12 | 4378369 | 4385463 | 298 | 16 | 22.55 | 0 (5) | 0 (7.05) | - |
| | SE_02_43600387 | chr5 | 142781350 | 142785986 | 387 | 5 | 10.79 | 3 (6) | 6.47 (12.94) | - |
| | SE_02_43600460 | chr1 | 154971472 | 154990869 | 460 | 37 | 19.08 | 2 (7) | 1.03 (3.61) | + |
| | SE_02_43600510 | chr1 | 182358446 | 182362440 | 510 | 4 | 10.02 | 1 (4) | 2.5 (10.02) | - |
| | SE_02_43600518 | chr17 | 48942249 | 48945892 | 518 | 9 | 24.70 | 2 (2) | 5.49 (5.49) | - |

* genome assembly: hg19

** average peak count per 10 kb;

***selected for gene regulation assay (line-specific, blue; conserved, red)

**Table S2. SE fragments matching the top-scoring enriched G4 motif: FIMO results.**

| Matched sequence* | chr | strand | start** | end ** | SE ID in SEdb 2.0 (cell line)*** |
|---|---|---|---|---|---|
| **GGGAGGGAGGGAGGGAGGG** | **chr19** | **-** | **13205999** | **13206017** | **SE_02_43600041 (HEK-293)** |
| *GGGAGGGAGGGAGGGAGGG* | *chrX* | + | *153235609* | *153235627* | *SE_02_43600122 (HEK-293)* |
| **GGGAGGGAGGGAGGGAGGG** | **chr19** | **-** | **13205995** | **13206013** | **SE_02_43600041 (HEK-293)** |
| **GGGAGGGAGAGAGGGAGGG** | **chr17** | **-** | **38225329** | **38225347** | **SE_01_03900154 (K-562)** |
| **GGGAGGGAGGGAAGGAGGG** | **chr19** | **-** | **18527873** | **18527891** | **SE_01_03900294 (K-562)** |
| **GGGAGGGAGGGAGGAAGGG** | **chr19** | **-** | **13168626** | **13168644** | **SE_02_43600041 (HEK-293)** |
| **GGGAGGAAGGGAGGGAGGG** | **chr19** | **+** | **13131209** | **13131227** | **SE_02_43600041 (HEK-293)** |
| **GAGAGGGAGGGAGGGAGGG** | **chr19** | **-** | **13206003** | **13206021** | **SE_02_43600041 (HEK-293)** |
| **GGGAGGGAGGGAGGGAAGG** | **chr9** | **+** | **139502774** | **139502792** | **SE_02_43600014 (HEK-293)** |
| **GGGAGGGAAGGAGGGAGGG** | **chr19** | **-** | **18527869** | **18527887** | **SE_01_03900294 (K-562)** |
| **GGGAGGGAAGGAGGGAGGG** | **chr9** | **+** | **139502608** | **139502626** | **SE_02_43600014 (HEK-293)** |
| *GGGAGGGAGGAAGGGAGGA* | *chr19* | - | *49643382* | *49643400* | *SE_01_03900471 (K-562)* |
| **GGGAGGGAGGGAGGGAGGA** | **chr18** | **+** | **46494867** | **46494885** | **SE_02_43600025 (HEK-293)** |
| **GGGAAGGAGGGAGGGAGGA** | **chr9** | **+** | **139502566** | **139502584** | **SE_02_43600014 (HEK-293)** |
| **GGGAGGGAGGGAAGGAGGG** | **chr9** | **+** | **139502778** | **139502796** | **SE_02_43600014 (HEK293)** |
| **GGGAGGGAGGGAAGGAGGG** | **chr9** | **+** | **139502919** | **139502937** | **SE_02_43600014 (HEK-293)** |
| **GGAAGGGAGGGAAGGAGGG** | **chr9** | **+** | **139502820** | **139502838** | **SE_02_43600014 (HEK-293)** |
| **GGAAGGGAGGGAGGAAGGG** | **chr9** | **+** | **139502737** | **139502755** | **SE_02_43600014 (HEK-293)** |
| **GGGAGGGAGAGAGGGAGAG** | **chr17** | **-** | **38280572** | **38280590** | **SE_01_03900154 (K-562)** |
| **GGGAGGGAGAGAGGGAGAG** | **chrX** | **+** | **39730397** | **39730415** | **SE_02_43600012 (HEK-293)** |
| **GGGAGGGAGAGAAGGAGGG** | **chr17** | **-** | **38225416** | **38225434** | **SE_01_03900154 (K-562)** |
| **GGAAGGGAGGGAGGGAGAG** | **chr17** | **-** | **38225353** | **38225371** | **SE_01_03900154 (K-562)** |
| **GGGAGGGAGAGAGGAAGGG** | **chr1** | **-** | **27855748** | **27855766** | **SE_01_03900056 (K-562)** |
| **GGGAAGGAGGGAGGGAGGA** | **chr9** | **+** | **139502612** | **139502630** | **SE_02_43600014 (HEK-293)** |
| **GGAAGGAAGGGAGGGAGGG** | **chr16** | **-** | **85629271** | **85629289** | **SE_01_03900005 (K-562)** |

* top-30 matches for motif GGGAGGGAGGGAGGG; the mismatching nucleotides are highlighted

**genome assembly: hg19.

***active, bold; poised, Italics.

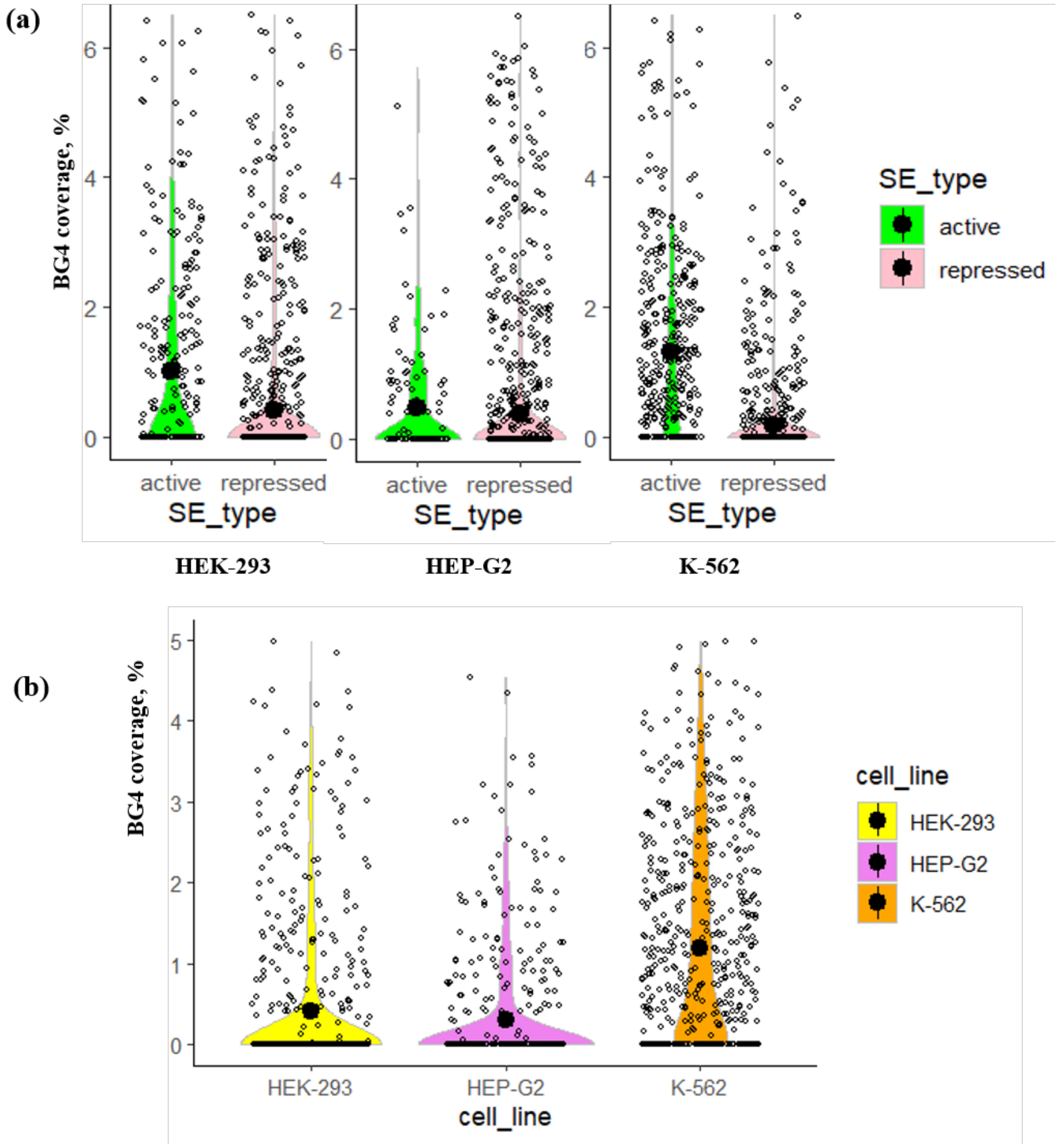


**Figure S2. BG4 coverage active vs repressed SEs (a) or poised and active SEs (b).** Active SEs are those present in SEdb 2.0 and confirmed by CAGE. Poised SEs are those present in SEdb 2.0 but lacking CAGE peaks. Repressed SEs are those absent in the cell line of interest but present in other cell lines, according to SEdb 2.0.

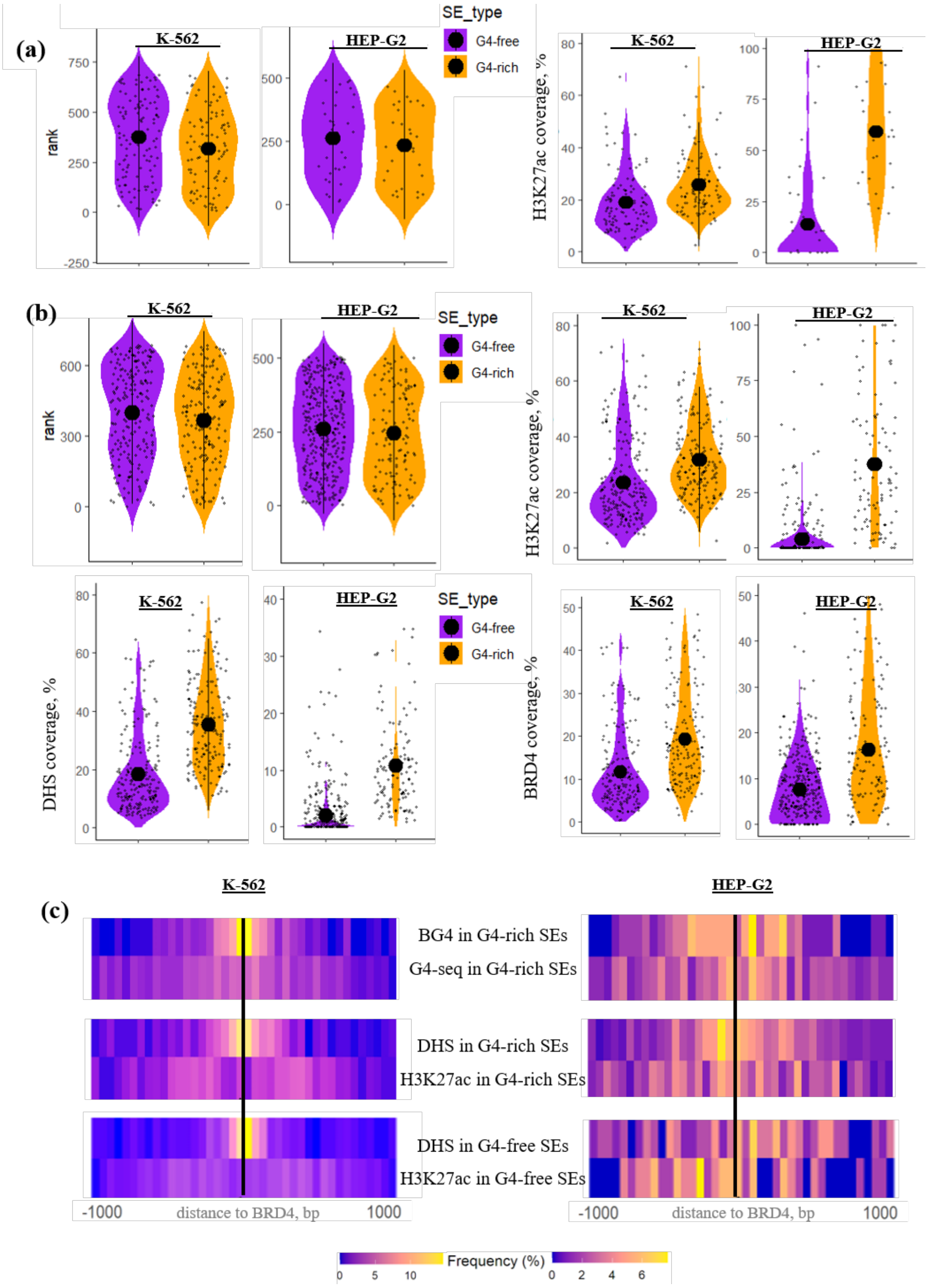


**Figure S3. G4-free and G4-rich SEs in K-562 and HEP-G2 cell lines: active and poised SEs.** (**a**) Ranks and normalized H3K27ac peak coverage in active G4-free SEs (116 in K-562 and 36 in HEP-G2) *versus* G4-rich ones (100 in K-562 and 32 in HEP-G2). (**b**) Ranks and normalized H3K27ac/DHS/BRD4 peak coverage in all (poised + active) G4-free SEs *versus* G4-rich ones. G4-free SEs are those lacking BG4 peaks (205 in K-562 and 349 in HEP-G2), and G4-rich ones are those showing top quartile BG4 coverage (172 in K-562 and 126 in HEP-G2). The difference between G4-free and G4-rich SEs is statistically significant ($p < 0.05$, Mann-Whitney test) in all cases, except for the ranks. (**c**) Distribution of BG4, G4-seq, DHS, and H3K27ac peaks relative to BRD4 occupancy sites in all (poised + active) SEs.

**Table S3. Sequences of all ODNs used in nucleosome assembly assays, BRD4-binding assays, and LLPS assays.**

| **code** | sequence (5′→3′)* |
|---|---|
| G4 | **GGGAGGGAGGGAGGG** |
| Cntr | TTCAACCAGTCTATG |
| ds26 | CAATCGGATCGAATTCGATCCGATTG |
| iM | CCCCCTCCCCCTCCCCCTCCCCC |
| dsG4 | Strand 1: atcacacttggca**GGGAGGGAGGGAGGG**atgcagctgatca<br>Strand 2: tgatcagctgcatccctccctccctccctgccaagtgtgat |
| dsCnr | Strand 1: atcacacttggctttcaaccagtctatgctgcagctgatca<br>Strand 2: tgatcagctgcagcatagactggttgaaagccaagtgtgat |
| G4-Widom | Strand 1:<br>ccgagcagctgatcacacttggca**GGGAGGGAGGGAGGG**atgcagctgatcacattggagtacatgcacag<br>gatgtatatatctgacacgtgcctggagactagggagtaatccccttggcggttaaaacgcggggggacagcgcgtacg<br>tgcgtttaagcggtgctagagctgtctacgaccaattgagcggcctcggcaccgggattctccagggcggccccccga<br>Strand 2:<br>tcggggggccgccctggagaatcccggtgccgaggccgctcaattggtcgtagacagctctagcaccgcttaaacgca<br>cgtacgcgctgtccccccgcgttttaaccgccaaggggattactccctagtctccaggcacgtgtcagatatatacatcc<br>tgtgcatgtactccaatgtgatcagctgcatccctccctccctccctgccaagtgtgatcagctgctcgg |
| Cntr-Widom | Strand 1: ccgagcagctgatcacacttggctttcaaccagtctatgctgcagctgatcacattggagtacatgcacag<br>gatgtatatatctgacacgtgcctggagactagggagtaatccccttgcccacccacccacccgggacagcgcgtacg<br>tgcgtttaagcggtgctagagctgtctacgaccaattgagcggcctcggcaccgggattctccagggcggccccccga<br>Strand 2:<br>tcggggggccgccctggagaatcccggtgccgaggccgctcaattggtcgtagacagctctagcaccgcttaaacgca<br>cgtacgcgctgtccccccgcgttttaaccgccaaggggattactccctagtctccaggcacgtgtcagatatatacatcc<br>tgtgcatgtactccaatgtgatcagctgcagcatagactggttgaaagccaagtgtgatcagctgctcgg |

*SE-G4, bold; nucleosome positioning sequence, underlined

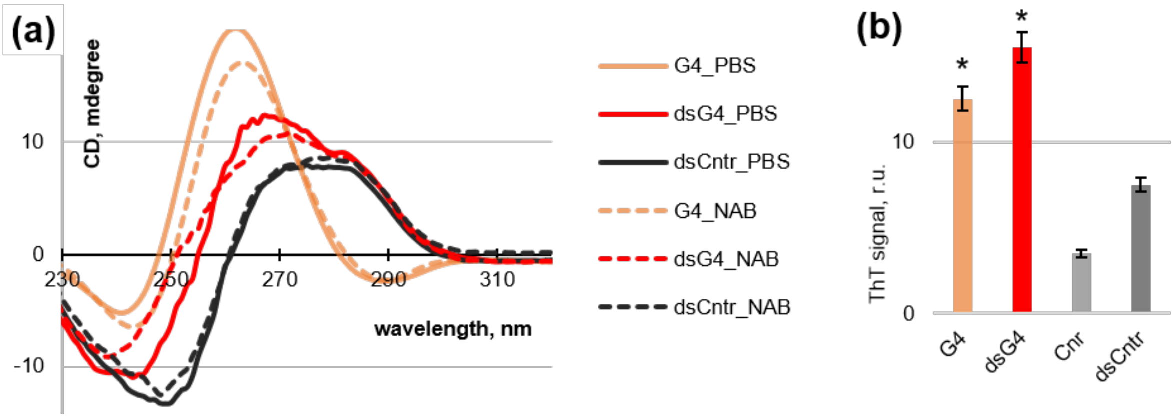


**Figure S4. Verification of G4 folding in dsG4 and its affinity for BRD4 by CD and ThT-assay.** (**a**) Circular dichroism spectra of 2 μM G4, dsG4 and dsCntr solutions in the phosphate-based working buffer (PBS) or the nucleosome assembly buffer (NAB). PBS: 20 mM sodium-phosphate buffer, pH 7.4, supplemented with 140 mM KCl; NAB: 20 mM Tris-HCl buffer, pH 8.0, supplemented with 250 mM NaCl. (**b**) ThT light-up in G4/Cntr and dsG4/Cntr samples (2 μM in PBS). ThT was added to a final concentration of 5 μM ThT, and fluorescence was measured at 490 nm upon excitation at 450 nm.

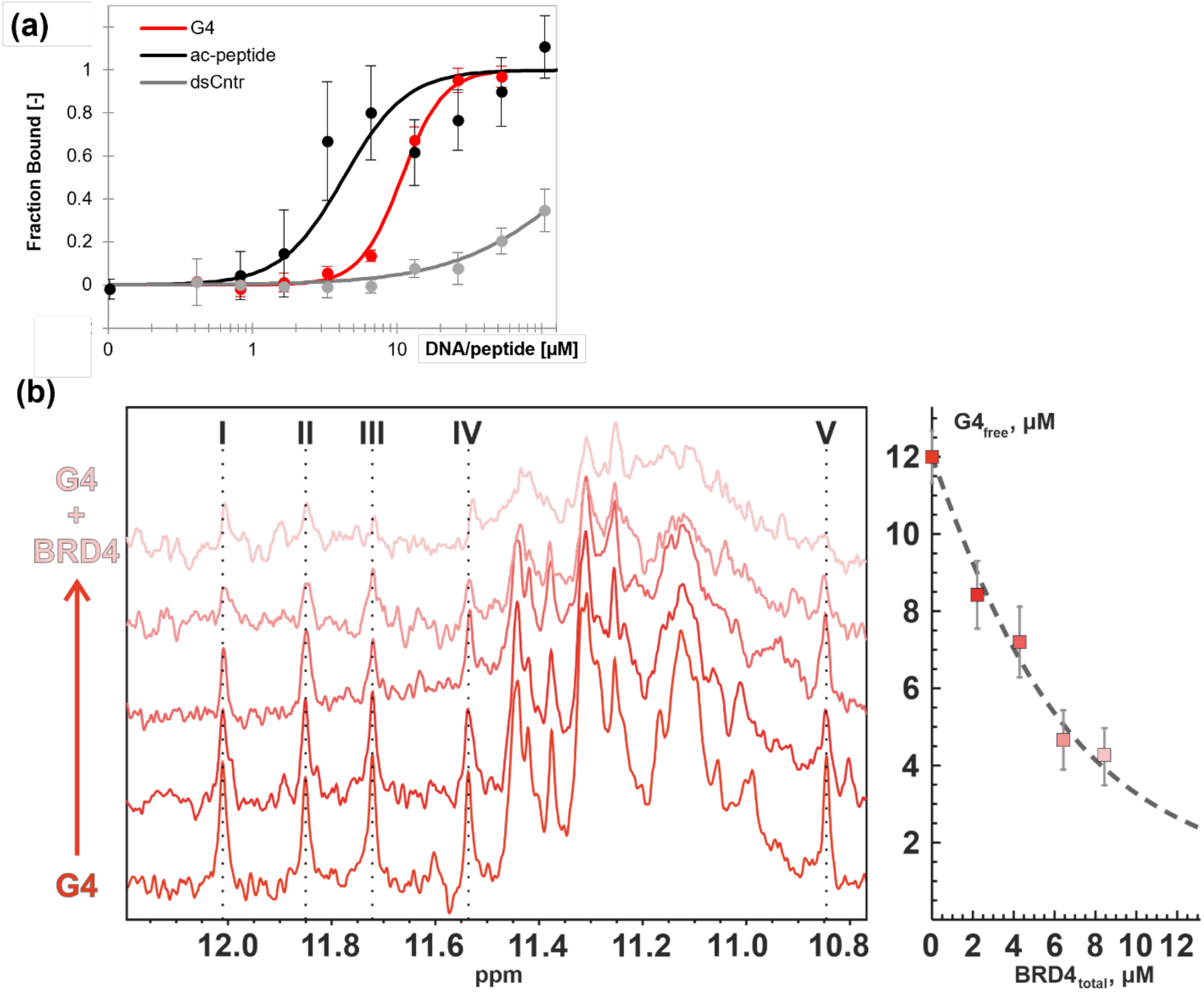


**Figure S5. Verification of G4 folding in dsG4 and its affinity for BRD4 by MST-assay and NMR. (a)** Microscale thermophoresis (MST)-based analysis of BRD4 interactions with the G4, control DNA, and the H3K18ac fragment (ac-peptide). Conditions: 50 nM labeled BRD4 in PBS with 0.05% Tween-20**. (b, left)** NMR spectra (700 MHz, 25°C) of 24 µM SE G4 solution in the phosphate-based working buffer (20 mM sodium-phosphate, pH 7.5, 80 mM KCl, and 5 % (v/v) $D_2O$) with increasing concentrations of BRD4 (0-8.4 µM) and minor admixtures of the protein storage buffer. The 1D $^1$H spectra were recorded using excitation sculpting water suppression scheme (pulse program *zgesgp* in Bruker library) with following parameters: spectral width of 17.5 kHz; repetition delay of 1.5 s; 32768 complex points; 3200 scans per FID; measurement time per one spectrum of 132 min. The spectra intensities were normalized to adjust for the minor buffer dependence and dilution. Final-point buffer conditions: 6.1 mM HEPES, 17.6 mM sodium-phosphate, pH 7.5, 24 mM NaCl, 70 mM KCl, 1.2% (v/v) Glycerol, 4.4 % (v/v) $D_2O$ and 0.12 mM DTT. **(b, right)** Dependence of free unimolecular SE G4 concentration on the total BRD4 concentration in the sample, adjusted for dilution. Error bars were calculated from spectral signal-to-noise ratios. Data is fitted with the simple binding model with $K_d$ = 6 ± 1 µM. The data are consistent only with a 1:2 stoichiometry of the BRD4/G4 complex.

## Text Box S1. Molecular modeling details.

Prior to docking, the G4 and dsG4 structures were optimized in SYBYL-X (Certara, USA) by Powell method with the following settings: partial charges and parameters for interatomic interactions were taken from Amber7ff02 force field; a non-bonded cut-off distance was equal to 8 Å; a distance-dependent dielectric function was used; the simplex method was employed for the initial optimization; the number of iterations was equal to 500; and the energy gradient convergence criterion was equal to 0.05 kcal/mol/Å.

Then, G4, dsG4, dsCntr, and the H3K18ac peptide structures were converted into an ICM objects, according to the ICM method, i.e., the molecular models were described using internal coordinates as variables. The parameters needed for interatomic energy calculation and the partial charges for atoms of the target were taken from mmff and ECEPP/3 (1) from forces field for DNA atoms and protein atoms, respectively. The biased probability Monte Carlo minimization procedure (2) was used for global energy optimization. The conformational stack obtained from docking was sorted using the energy scoring function calculated as follows:

$$E_{bind} = E_{intl} + VW_{Int} + 0.6*N_{flex} + \alpha_1 * Sol_{El} + \alpha_2 * H_{bond} + \alpha_3 * H_{phob} + \alpha_4 * D_{solv} \quad (1),$$

where $E_{intl}$ is the differences between internal energies in the bound and unbound states, $VW_{Int}$ is van der Waals interaction energy, $N_{flex}$ is the number of rotatable torsions, $0.6*N_{flex}$ is the conformational entropy loss upon binding, $Sol_{El}$ is the electrostatic contribution to the solvation energy change upon binding, $H_{bond}$ is Hydrogen Bond energy, $H_{phob}$ is the hydrophobic energy arising from surface exposure to water, $D_{solv}$ is the desolvation of exposed h-bond donors and acceptors, and $\alpha_i$ are regression constants.

In the cases of G4, dsG4 and dsCntr, a two-step docking procedure was used. Step 1 included 'rigid' docking using Hex 8.0.0. software (3) and post-processing MM minimization using the OPLS force field. The rigid docking was performed with the following settings: FFT Mode: 3D, receptor range:180°, ligand range: 180°, twist range: 360°, distance range: 40°, angular increments: 7.5° for DNA and BRD4 rotational angles, and 5.5 for the twist angles. At step 2, the complexes selected based on the scoring function during post-processing at step 1 were minimized using SYBYL software (Certara, USA) and Powell method. Parameters for interatomic interactions and partial charges on the atoms were taken from Amber7ff02 force field. After step 2, the final models of the G4-BRD4 complexes were selected based on the sum of the electrostatic and van der Waals contributions to binding energies. At this step, the dielectric constant value of 4 was used to account for the media contribution.

To investigate the possible competition between the Kac pocket-binding ligand JQ1 and dsG4 for BRD4, additional docking experiments were performed using ICM. First, dsG4 was docked to the BRD4-JQ1 complex (PDB: 4QZS), in which JQ1 occupies the Kac pocket of BD1 (4). The dsG4 position in the best-scoring conformation of the resulting triple complex was similar to that obtained in the absence of JQ1. Second, JQ1 was docked to the BRD4-dsG4 complex. In the resulting triple complex, JQ1 occupied the Kac pocket of BD2 without interfering with dsG4, and its position was similar to that described previously (PDB: 4QZS) (5). For these experiments, ligand electron density distribution was calculated using DFT/B3LYP/6-311+g(2d,2p) with implicit consideration of the solvent effect with application of the conductor-like polarizable continuum model (6). Then, Merz-Singh-Kollman scheme (7) was applied to the obtained electron density distribution for calculation of the grid for the electrostatic potential fitting with the following parameters: (6/41=10) - the number of surfaces around atoms and (6/42=17) - the density of test points on these surfaces. The Restrained ElectroStatic Potential method (8) was applied to fitting of the obtained grid for calculation of partial atomic charges. All quantum mechanics simulations were carried out using Gaussian 16 program (9).

Stability of the models of the G4/dsG4/dsCntr complexes with BRD4 were verified by molecular dynamics (MD) simulations using Amber 22 software. The influence of the solvent was simulated using the OPC3 water molecule model (10). The simulation was performed with periodical boundary conditions and a rectangular box. The buffer between the BRD4-DNA/H3K18ac complex and the periodic box wall was at least 15 Å. Potassium ions were added to neutralize the negative charge of the DNA backbone and stabilize the G4 structure. The parameters needed for interatomic energy calculation were taken from the force fields OL15 (11, 12) for DNA and ff19SBonlysc (13) for the H3K18ac and BRD4.

The system was initially minimized in two steps. First, the location of the solvent molecules was optimized using 1000 steps (500 steps of steepest descent followed by 500 steps of conjugate gradient). At that stage, the mobility of all solute atoms s was restrained with a force constant of 500 kcal*mol$^{-1}$*Å$^{-2}$. The second step of the optimization was realized without any restriction using 2500 steps (1000 steps of steepest decent and 1500 steps of conjugate gradient). Then, gradual heating to 300 K during 20 ps was performed. At that stage, to avoid wild fluctuations of the investigated system, weak harmonic restrains were used with a force constant of 10 kcal*mol$^{-1}$*Å$^{-2}$ for all atoms except for the solvent atoms. SHAKE algorithm was applied to constrain bonds to hydrogen atoms, and that allowed to use 2 fs step. Scaling of nonbonded 1–4 van der Waals and electrostatic interactions was performed using the standard Amber values. The cutoff distance for non-bonded interactions was equal to 10 Å. The long-range electrostatics were calculated using the particle mesh Ewald method. The MD simulations in production phase were carried out using constant

temperature (T = 300 K) and constant pressure (p = 1 atm) over 100 ns. To control the temperature, Langevin thermostat was used with the collision frequency of 1 $ps^{-1}$.

The Coulomb and van der Waals contributions from energy of interaction of the ligands with the targets were estimated using the lie command from the CPPTRAJ module, with the dielectric constant for the electrostatic contribution equal to 4. Solvation energy: its polar and hydrophobic components were estimated by using the GBSA approach. The polar contribution (EGB) was computed using the Generalized Born (GB) method and the algorithm developed by Onufriev et al. for calculating the effective Born radii (14). The non-polar contribution to the solvation energy (Esurf), which includes solute-solvent van der Waals interactions and the free energy of cavity formation in solvent, was estimated from a solvent-accessible surface area (SASA).


1. Arnautova,Y.A., Jagielska,A. and Scheraga,H.A. (2006) A New Force Field (ECEPP-05) for Peptides, Proteins, and Organic Molecules. J Phys Chem B, 110, 5025–5044.
2. Abagyan,R. and Totrov,M. (1994) Biased Probability Monte Carlo Conformational Searches and Electrostatic Calculations for Peptides and Proteins. J Mol Biol, 235, 983–1002.
3. Macindoe,G., Mavridis,L., Venkatraman,V., Devignes,M.-D. and Ritchie,D.W. (2010) HexServer: an FFT-based protein docking server powered by graphics processors. Nucleic Acids Res, 38, W445–W449.
4. Mishra, N.K., Urick, A.K., Ember, S.W., Schonbrunn, E., Pomerantz, W.C. (2014) Fluorinated aromatic amino acids are sensitive (19)f NMR probes for bromodomain-ligand interactions.. ACS Chem Biol, 9, 2755-2760
5. Filippakopoulos, P., Qi, J., Picaud, S., Shen, Y., Smith, W.B., Fedorov, O., Morse, E.M., Keates, T., Hickman, T.T., Felletar, I., Philpott, M., Munro, S., McKeown, M.R., Wang, Y., Christie, A.L., West, N., Cameron, M.J., Schwartz, B., Heightman, T.D., La Thangue, N., French, C.A., Wiest, O., Kung, A.L., Knapp, S., Bradner, J.E. (2010) Selective inhibition of BET bromodomains. Nature, 468,1067-1073
6. Barone V. and Cossi M.. Quantum Calculation of Molecular Energies and Energy Gradients in Solution by a Conductor (1998) Solvent Model J. Phys. Chem. A, 102, 11, 1995–2001
7. Singh U. C. and Kollman P. A. (1984) An approach to computing electrostatic charges for molecules. J. Comp. Chem., 5, 129-45.
8. Bayly C.I., Cieplak P., Cornell W.D., Kollman P.A. (1993) A Well-Behaved Electrostatic Potential Based Method Using Charge Restraints For Determining Atom-Centered Charges: The RESP Model. J. Phys. Chem. 97, 10269-10280
9. Gaussian 16, Revision C.01, Frisch, M. J.; Trucks, G. W.; Schlegel, H. B.; Scuseria, G. E.; Robb, M. A.; Cheeseman, J. R.; Scalmani, G.; Barone, V.; Petersson, G. A.; Nakatsuji, H.; Li, X.; Caricato, M.; Marenich, A. V.; Bloino, J.; Janesko, B. G.; Gomperts, R.; Mennucci, B.; Hratchian, H. P.; Ortiz, J. V.; Izmaylov, A. F.; Sonnenberg, J. L.; Williams-Young, D.; Ding, F.; Lipparini, F.; Egidi, F.; Goings, J.; Peng, B.; Petrone, A.; Henderson, T.; Ranasinghe, D.; Zakrzewski, V. G.; Gao, J.; Rega, N.; Zheng, G.; Liang, W.; Hada, M.; Ehara, M.; Toyota, K.; Fukuda, R.; Hasegawa, J.; Ishida, M.; Nakajima, T.; Honda, Y.; Kitao, O.; Nakai, H.; Vreven, T.; Throssell, K.; Montgomery, J. A., Jr.; Peralta, J. E.; Ogliaro, F.; Bearpark, M. J.; Heyd, J. J.; Brothers, E. N.; Kudin, K. N.; Staroverov, V. N.; Keith, T. A.; Kobayashi, R.; Normand, J.; Raghavachari, K.; Rendell, A. P.; Burant, J. C.; Iyengar, S. S.; Tomasi, J.; Cossi, M.; Millam, J. M.; Klene, M.; Adamo, C.; Cammi, R.; Ochterski, J. W.; Martin, R. L.; Morokuma, K.; Farkas, O.; Foresman, J. B.; Fox, D. J. Gaussian, Inc., Wallingford CT, 2016.
10. Izadi,S. and Onufriev,A. V (2016) Accuracy limit of rigid 3-point water models. J Chem Phys, 145, 074501.
11. Zgarbová,M., Luque,F.J., Šponer,J., Cheatham,T.E., Otyepka,M. and Jurečka,P. (2013) Toward Improved Description of DNA Backbone: Revisiting Epsilon and Zeta Torsion Force Field Parameters. J Chem Theory Comput, 9, 2339–2354.
12. Zgarbová,M., Šponer,J., Otyepka,M., Cheatham,T.E., Galindo-Murillo,R. and Jurečka,P. (2015) Refinement of the Sugar-Phosphate Backbone Torsion Beta for AMBER Force Fields Improves the Description of Z- and B-DNA. J Chem Theory Comput, 11, 5723–36.
13. Tian,C., Kasavajhala,K., Belfon,K.A.A., Raguette,L., Huang,H., Migues,A.N., Bickel,J., Wang,Y., Pincay,J., Wu,Q., et al. (2020) ff19SB: Amino-Acid-Specific Protein Backbone Parameters Trained against Quantum Mechanics Energy Surfaces in Solution. J Chem Theory Comput, 16, 528–552.
14. Onufriev,A., Bashford,D. and Case,D.A. (2000) Modification of the Generalized Born Model Suitable for Macromolecules. J Phys Chem B, 104, 3712–3720.

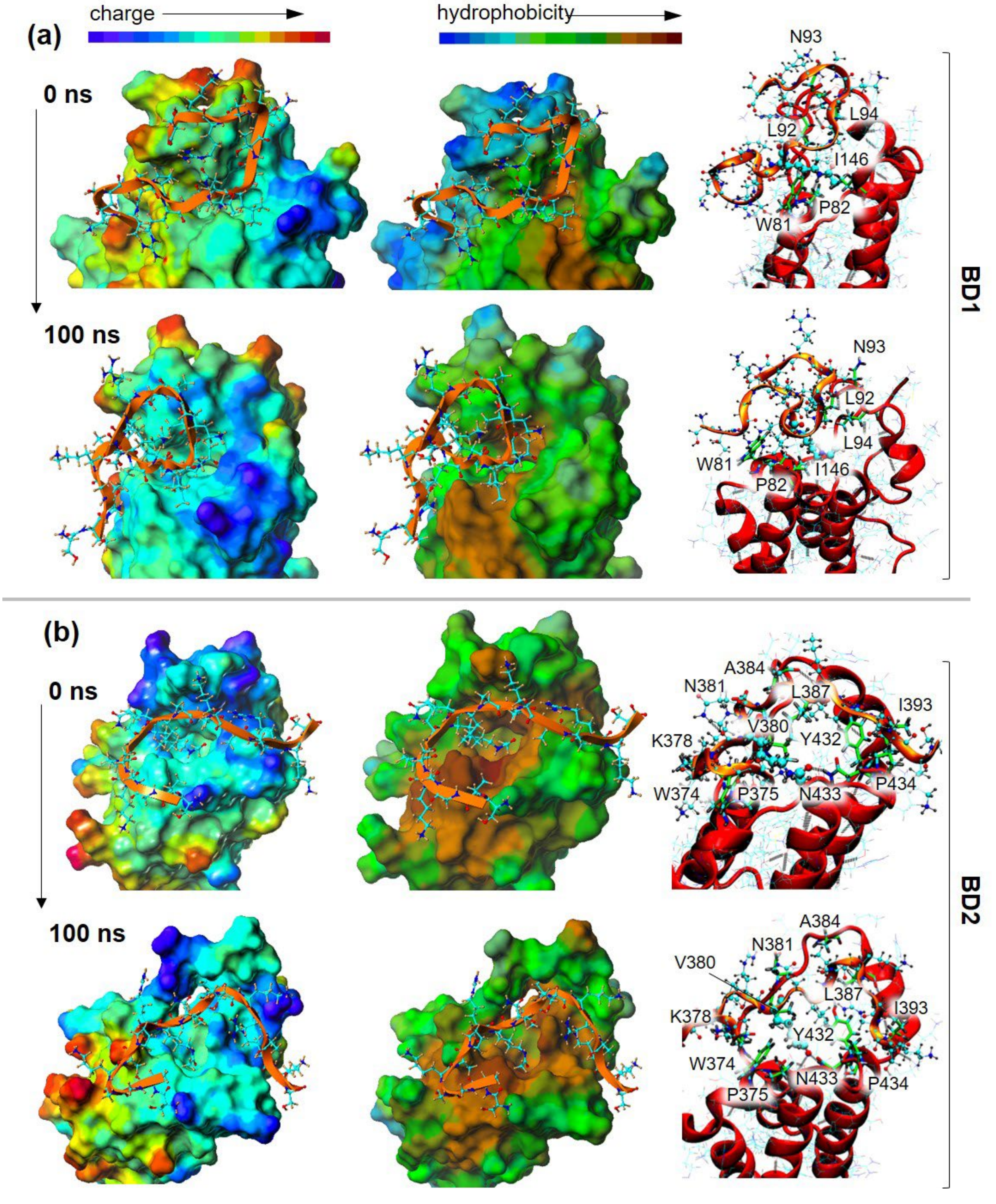


**Figure S6. Models of BRD4 complexes with its cognate binding partner – ac-peptide.** (a) Complexes of the H3K18ac fragment S10-S28 with BD1. (b) Complexes with BD2. Top panels in (a) and (b): starting conformations obtained from docking. Bottom panels: 100 ns MD simulation snapshots. Left: the protein surface is colored according to its electrostatic potential. Middle: the surface is colored according to its hydrophobic potential. Right: the surface is not shown, the backbone is shown using the ribbon rendering (red), and the key side chain residues are marked. The peptide backbone is brown in all images. H-bonds are shown as dashed lines.

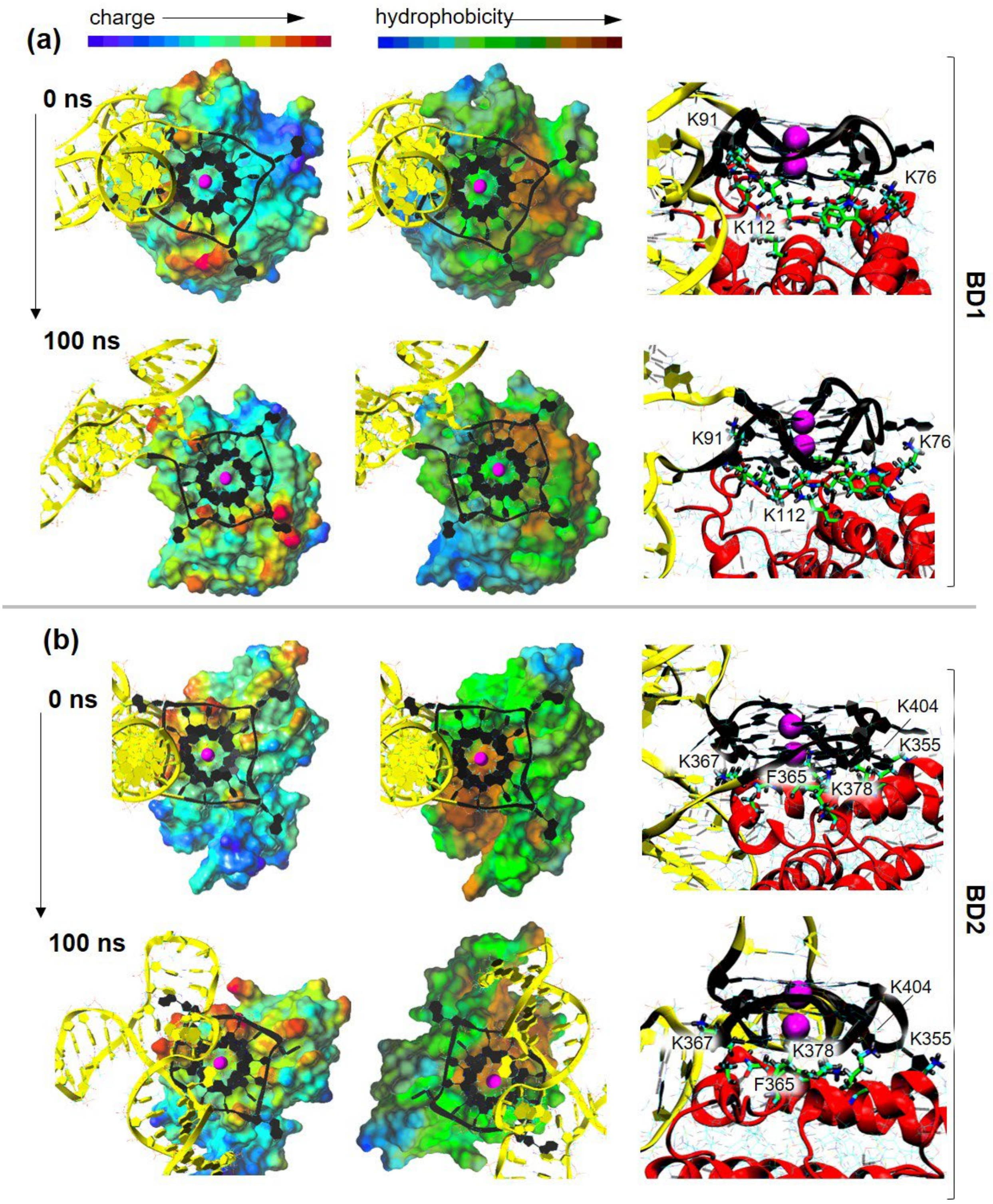


**Figure S7. Models of BRD4 complexes with dsG4.** (a) Complexes of dsG4 with BRD4 BD1. (b) Complexes with BD2. Top panels in (a) and (b): starting conformations obtained from docking. Bottom panels: 100 ns MD simulation snapshots. Left: the protein surface is colored according to its electrostatic potential. Middle: the surface is colored according to its hydrophobic potential. Right: the surface is not shown, the backbone is shown using the ribbon rendering (red), and the key side chain residues are marked. The G4 is black, while its flanks and the opposing strand are yellow. Potassium ions are purple. H-bonds are shown as dashed lines.

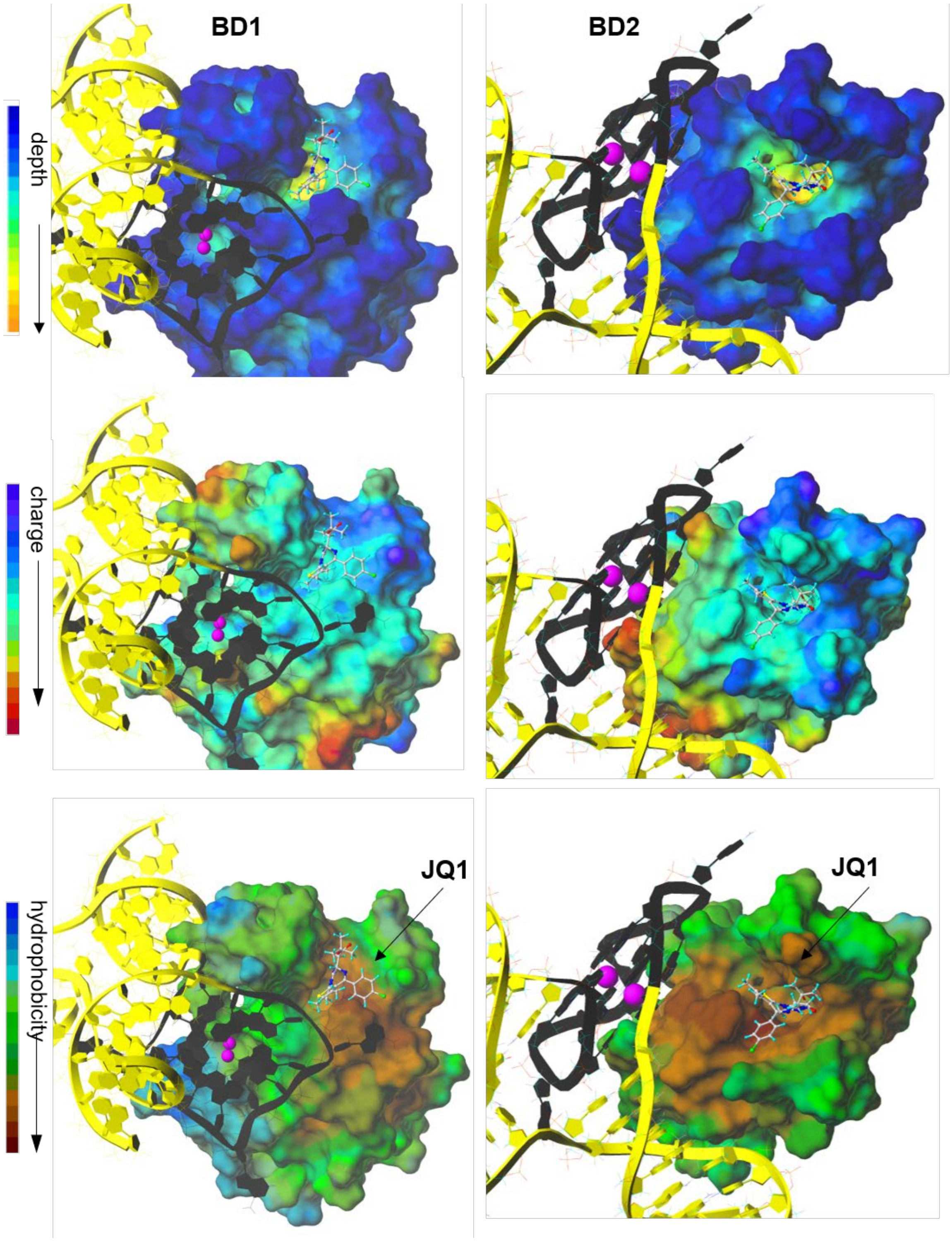


**Figure S8. Positions of dsG4 and JQ1 on BRD4 BD1 (left) and BD2 (right).** The protein surface is colored according to its depth/electrostatic potential/hydrophobicity. The G4 is black, its duplex flanks are yellow, potassium ions are purple., and JQ1 is shown in full-atom representation.

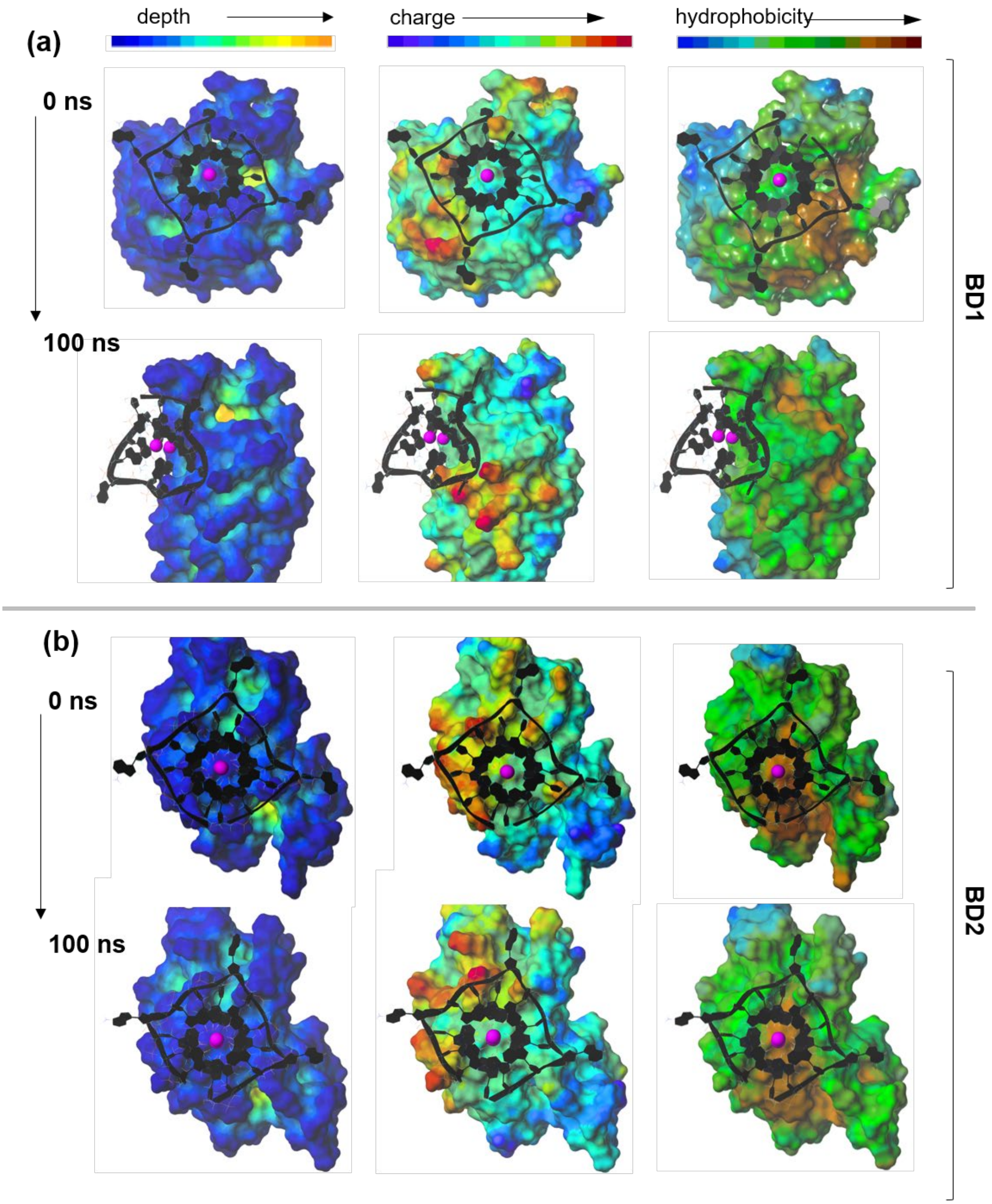


**Figure S9. Models of BRD4 complexes with the G4 (no flanks).** (a) Complexes of the SE G4 with BRD4 BD1. (b) Complexes with BD2. Top panels in (a) and (b): starting conformations obtained from docking. Bottom panels: 100 ns MD simulation snapshots. Left: the protein surface is colored according to its depth. Middle: the surface is colored according to its electrostatic potential. Right: the surface is colored according to its hydrophobic potential. The G4 is black. Potassium ions are purple.

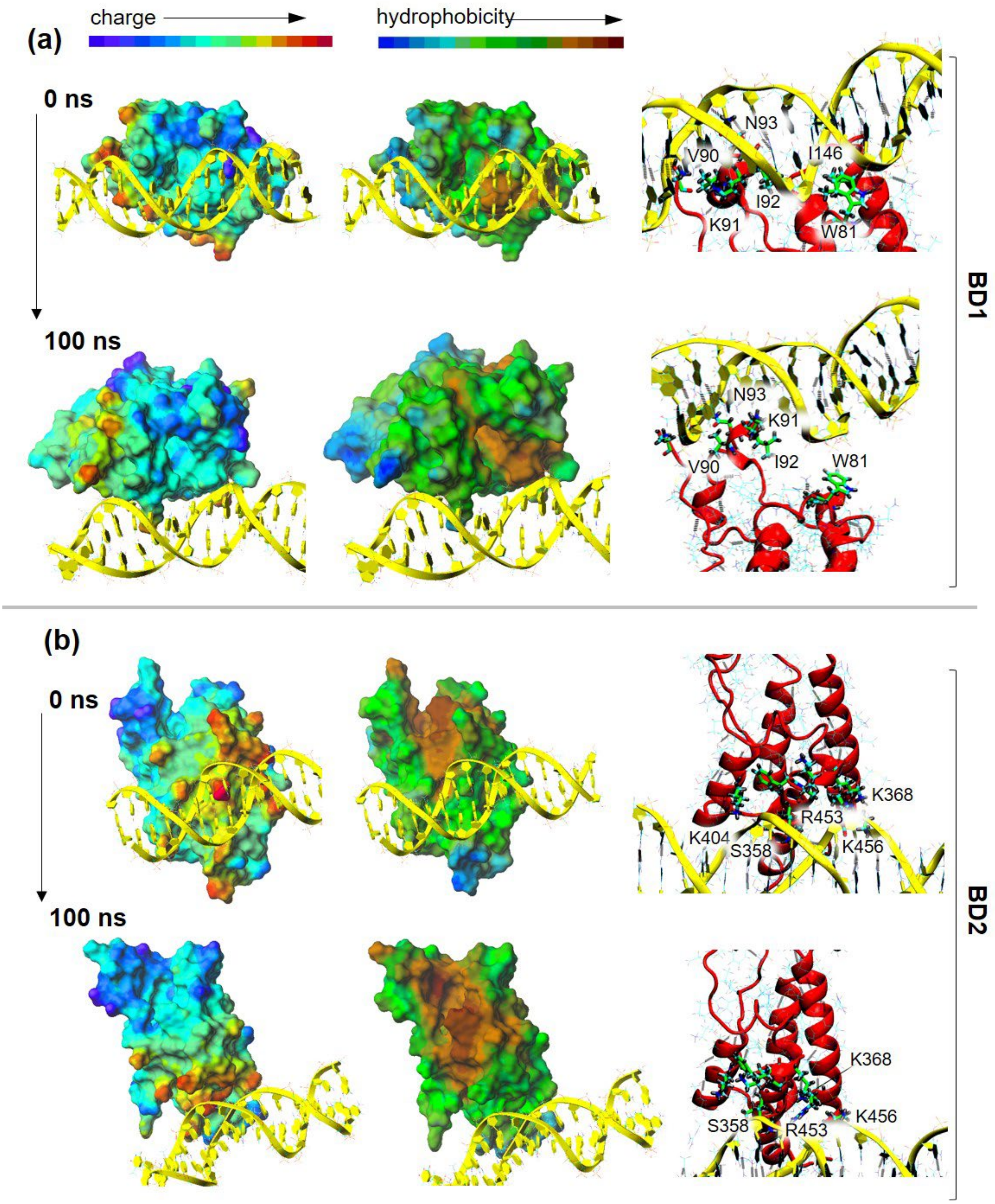


**Figure S10. Models of BRD4 complexes with dsCntr.** (a) Complexes of dsCntr with BRD4 BD1. (b) Complexes with BD2. Top panels in (a) and (b): starting conformations obtained from docking. Bottom panels: 100 ns MD simulation snapshots. Left: the protein surface is colored according to its electrostatic potential. Middle: the surface is colored according to its hydrophobic potential. Right: the surface is not shown, the backbone is shown using the ribbon rendering (red), and the key side chain residues are marked. dsCntr is yellow. H-bonds are shown as dashed lines.

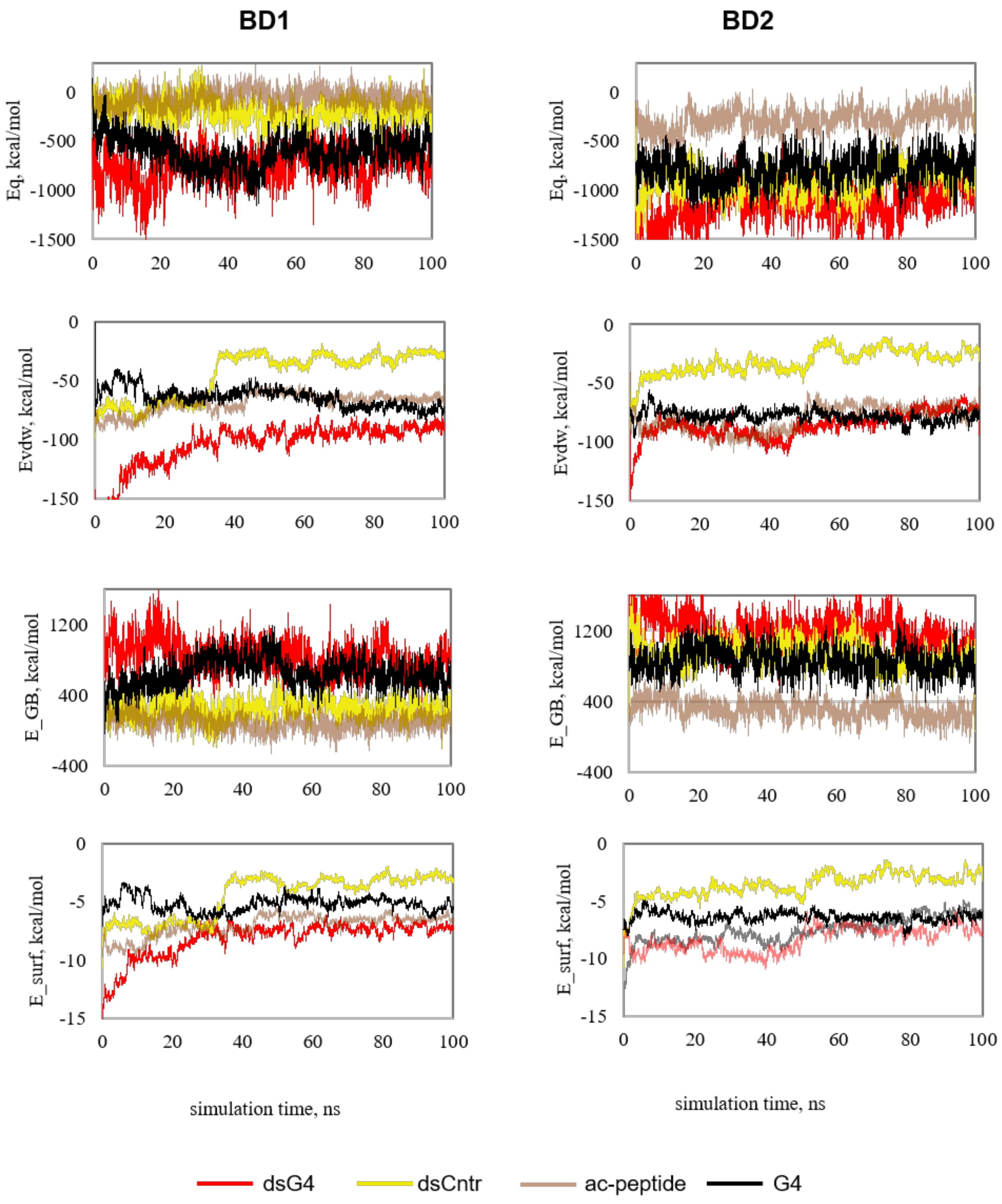


**Figure S11. Comparison of ac-peptide, G4, dsG4, and dsCntr binding with BRD4 BD1 (left) and BD2 (right): contributions to the binding energy.** $E_q$, electrostatic energy; $E_{vdw}$, van der Waals energy, $E_{GB}$ polar contribution to the solvation energy; $E_{surf}$, non-polar contribution to the solvation energy. The energy plots were smoothed using the moving average method (span = 5).

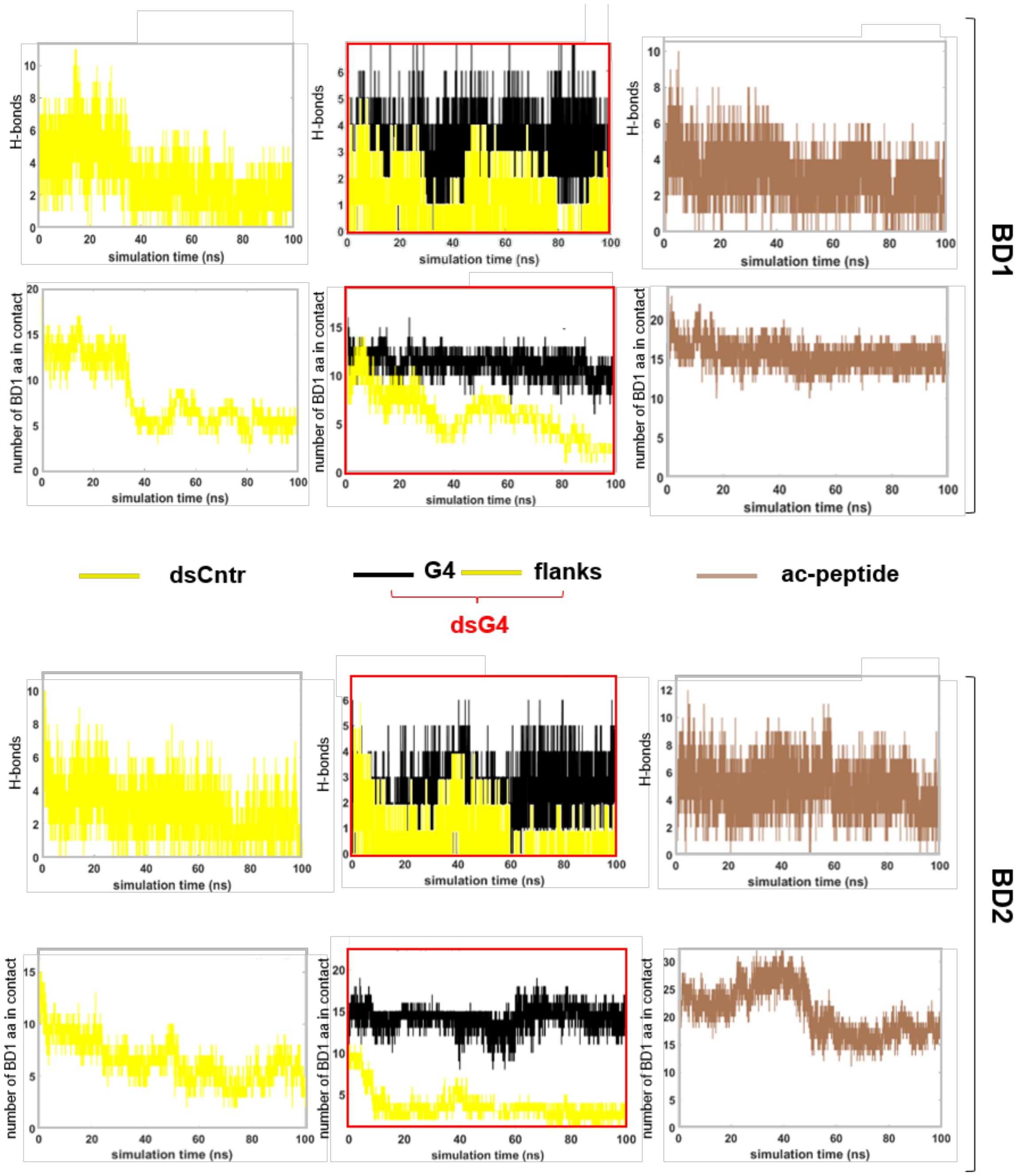


**Figure S12. Comparison of H3K18ac, SE-G4, and dsDNA binding with BRD4 BD1 (top) and BD2 (bottom): H-bonds and BD contacts.**

**Table S4. Detailed analysis of ac-peptide and dsG4 binding with BRD4: contributions of individual residues.**

**ac-peptide residues**

| Domain | Term (kcal/mol) | S10 | T11 | G12 | G13 | K14 | A15 | P16 | R17 | $K18^{ac}$ | Q19 | L20 | A21 | T22 | K23 | A24 | A25 | R26 | K27 | S28 |
|---|---|---|---|---|---|---|---|---|---|---|---|---|---|---|---|---|---|---|---|---|
| BD1 | Eq | -1.3 | 0.1 | -0.2 | 0.1 | 0.9 | -1.3 | 0.2 | -10.3 | -2.4 | 0.5 | 1.2 | 0.7 | -1.2 | -7.1 | -0.6 | -0.4 | -0.4 | -1.5 | -2.2 |
| BD1 | Evdw | -1.1 | -2.5 | -1.8 | -0.4 | -0.4 | -1.2 | -6.5 | -8.7 | -17.7 | -2.5 | -6.9 | -4.4 | -3.7 | -1.6 | -1.9 | -1.5 | -2.9 | -2.8 | -0.4 |
| BD1 | Esum | -2.3 | -2.4 | -2.0 | -0.3 | 0.6 | -2.5 | -6.3 | -19.0 | -20.1 | -2.0 | -5.8 | -3.7 | -4.8 | -8.7 | -2.5 | -1.9 | -3.3 | -4.3 | -2.6 |
| BD2 | Eq | -5.3 | -1.0 | -0.4 | 0.0 | 1.1 | -1.7 | -2.0 | -18.9 | -2.9 | -2.1 | -0.9 | -1.5 | 1.1 | -1.2 | 1.6 | -0.5 | -21.2 | -3.9 | -0.7 |
| BD2 | Evdw | -1.3 | -3.1 | -1.4 | -1.1 | -4.2 | -2.6 | -2.8 | -4.6 | -18.0 | -4.7 | -10.0 | -4.7 | -4.0 | -1.0 | -1.1 | -1.3 | -10.5 | -3.0 | -1.3 |
| BD2 | Esum | -6.6 | -4.1 | -1.7 | -1.1 | -3.1 | -4.3 | -4.8 | -23.5 | -20.9 | -6.8 | -11.0 | -6.1 | -2.9 | -2.3 | 0.5 | -1.8 | -31.7 | -6.9 | -2.1 |

**dsG4 residues**

| Domain | Term (kcal/mol) | G1 | G2 | G3 | A4 | G5 | G6 | G7 | A8 | G9 | G10 | G11 | A12 | G13 | G14 | G15 |
|---|---|---|---|---|---|---|---|---|---|---|---|---|---|---|---|---|
| BD1 | Eq | -5.6 | -1.6 | -0.4 | -1.6 | -9.1 | -3.2 | -1.1 | -6.5 | -16.8 | -7.1 | 0.4 | 0.3 | 1.0 | -2.3 | -5.4 |
| BD1 | Evdw | -15.3 | -1.2 | -0.1 | -0.1 | -9.1 | -2.1 | -0.2 | -1.5 | -19.9 | -2.2 | -0.2 | -0.3 | -16.4 | -7.1 | -2.4 |
| BD1 | Esum | -20.9 | -2.8 | -0.5 | -1.7 | -18.2 | -5.3 | -1.3 | -8.1 | -36.7 | -9.3 | 0.2 | 0.0 | -15.4 | -9.4 | -7.7 |
| BD2 | Eq | -3.4 | -16.5 | -11.4 | -4.1 | -0.7 | -2.5 | -8.3 | -15.2 | -1.4 | 3.3 | -6.6 | -8.4 | -1.9 | -4.4 | -1.7 |
| BD2 | Evdw | -0.5 | -2.3 | -8.6 | -0.3 | -0.1 | -1.0 | -9.4 | -22.5 | -0.5 | -2.4 | -14.6 | -2.5 | -0.3 | -1.6 | -13.5 |
| BD2 | Esum | -3.9 | -18.8 | -20.1 | -4.4 | -0.8 | -3.5 | -17.8 | -37.7 | -1.9 | 0.9 | -21.2 | -10.9 | -2.2 | -6.0 | -15.2 |

**Table S5. Detailed analysis of ac-peptide and dsG4 binding with BRD4 BD1 (grey background) and BD2 (white background): H-bonding.** The percentage of MD simulation snapshot with the H-bond of interest is specified for each donor-acceptor pair. BRD4 residues are blue, G4 residues are black, duplex flanks from the G4-containing strand (F) and the complimentary strand (FC) are yellow. Residue numbers are given in parentheses. The numbering scheme in dsG4 is summarized in the bottom box. Upper indices of the donor/acceptor groups: sr, side residue; b, peptide backbone; ac, acetyl; sp, sugar-phosphate backbone; nb, nucleobase.

**BRD4-ac-peptide from H3K18ac**

BD1 (grey background):

| donor | acceptor | sn.% |
|---|---|---|
| $NH_2^{sr}$ (R17) | $O^{sr}$ (Q84) | 57 |
| $NH_2^{sr}$ (R17) | $O^{sr}$ (Q85) | 53 |
| $NH_2^{sr}$ (N140) | $O^{ac}$ ($K18^{ac}$) | 53 |
| $OH^{sr}$ (T22) | $O^{b}$ (I92) | 42 |
| $NH_2^{sr}$ (N93) | $O^{b}$ (A25) | 26 |
| $NH^{b}$ (K27) | $O^{b}$ (K91) | 22 |
| $NH_2^{sr}$ (N93) | $O^{b}$ (A24) | 20 |
| $NH_2^{sr}$ (K91) | $O^{b}$ (K27) | 8 |
| $NH^{sr}$ ($K18^{ac}$) | $O^{b}$ (P82) | 7 |
| $NH_2^{sr}$ (K23) | $O^{b}$ (Y139) | 6 |
| $OH^{sr}$ (S10) | $O^{b}$ (P86) | 4 |
| $NH^{sr}$ (R17) | $O^{sr}$ (Q84) | 4 |
| $NH_2^{sr}$ (K23) | $O^{sr}$ (D96) | 2 |

BD2 (white background):

| donor | acceptor | sn.% | donor | acceptor | sn.% |
|---|---|---|---|---|---|
| | | | $NH^{sr}$ (R17) | $O^{sr}$ (D381) | 14 |
| | | | $NH_2^{sr}$ (Q19) | $O^{b}$ (I385) | 13 |
| | | | $NH_2^{sr}$ (N433) | $O^{ac}$ ($K18^{ac}$) | 12 |
| | | | $NH^{sr}$ (W374) | $O^{b}$ (G12) | 12 |
| | | | $NH^{ac}$ ($K18^{ac}$) | $O^{b}$ (K378) | 12 |
| | | | $NH_2^{sr}$ (K378) | $O^{b}$ (P16) | 12 |
| | | | $NH_2^{sr}$ (Q19) | $O^{sr}$ (D381) | 8 |
| | | | $NH_2^{sr}$ (K378) | $O^{b}$ (K14) | 8 |
| | | | $NH_2^{sr}$ (R17) | $O^{sr}$ (E383) | 7 |
| | | | $NH_2^{sr}$ (R26) | $O^{b}$ (G386) | 7 |
| | | | $NH_2^{sr}$ (K378) | $O^{b}$ (A15) | 6 |
| | | | $NH_2^{sr}$ (R17) | $O^{sr}$ (E383) | 6 |
| | | | $NH_2^{sr}$ (Q19) | $O^{sr}$ (D381) | 6 |
| | | | $OH^{sr}$ (T11) | $O^{sr}$ (E438) | 6 |
| $NH^{b}$ (D381) | $O^{ac}$ ($K18^{ac}$) | 52 | $NH^{sr}$ (H437) | $O^{b}$ (A21) | 5 |
| $NH_2^{sr}$ (R26) | $O^{sr}$ (D389) | 38 | $NH^{b}$ (K18ac) | $O^{b}$ (P379) | 5 |
| $NH^{sr}$ (R17) | $O^{sr}$ (D381) | 29 | $OH^{sr}$ (T11) | $O^{sr}$ (E438) | 4 |
| $NH^{ac}$ ($K18^{ac}$) | $O^{b}$ (P375) | 27 | $NH^{b}$ (S10) | $O^{sr}$ (E438) | 4 |
| $NH_2^{sr}$ (R17) | $O^{sr}$ (D381) | 25 | $OH^{sr}$ (Y390) | $O^{ac}$ ($K18^{ac}$) | 4 |
| $NH^{sr}$ (R26) | $O^{sr}$ (D389) | 24 | $NH^{b}$ (T11) | $O^{sr}$ (E438) | 4 |
| $NH^{sr}$ (R26) | $O^{sr}$ (D389) | 22 | $NH^{b}$ (S10) | $O^{sr}$ (E438) | 3 |
| $NH_2^{sr}$ (N433) | $O^{b}$ (I20) | 20 | $NH^{b}$ (T11) | $O^{sr}$ (E438) | 3 |
| $OH^{sr}$ (Y432) | $O^{b}$ (I20) | 17 | $NH_2^{sr}$ (K27) | $O^{sr}$ (D392) | 2 |
| $NH_2^{sr}$ (R26) | $O^{sr}$ (D389) | 17 | $NH^{sr}$ (R26) | $O^{b}$ (Y432) | 2 |
| $NH_2^{sr}$ (R17) | $O^{sr}$ (D381) | 16 | $NH^{b}$ (G12) | $S^{sr}$ (M442) | 2 |
| $NH^{b}$ ($K18^{ac}$) | $O^{sr}$ (D381) | 15 | $NH_2^{sr}$ (K27) | $O^{sr}$ (D392) | 2 |

**BRD4-dsG4**

BD1 (grey background):

| donor | acceptor | sn.% | donor | acceptor | sn.% |
|---|---|---|---|---|---|
| $NH^{sr}$ (Q85) | $O6^{sp}$ (G13) | 63 | $NH^{b}$ (S51) | $O^{sp}$ (F-3) | 2 |
| $NH_2^{sr}$ (N44) | $O^{sp}$ (G1) | 39 | $NH_2^{sr}$ (K76) | $O5'^{sp}$ (G9) | 2 |
| $NH^{sr}$ (W75) | $O^{sp}$ (G9) | 30 | | | |
| $NH^{b}$ (N44) | $O^{sp}$ (G1) | 28 | | | |
| $NH_2^{sr}$ (Q84) | $O4'^{sp}$ (G9) | 27 | | | |
| $NH_2^{sr}$ (K76) | $O^{sp}$ (G9) | 23 | | | |
| $NH_2^{sr}$ (N52) | $O^{sp}$ (F-4) | 13 | | | |
| $NH^{b}$ (N44) | $O3'^{sp}$ (F-1) | 13 | | | |
| $NH_2^{sr}$ (K102) | $O^{sp}$ (FC+7) | 13 | | | |
| $NH_2^{sr}$ (K76) | $O^{sp}$ (G10) | 10 | | | |
| $NH_2^{sr}$ (K112) | $O4'^{sp}$ (G5) | 8 | | | |
| $NH_2^{sr}$ (K76) | $O^{sp}$ (G10) | 8 | | | |
| $NH_2^{sr}$ (N54) | $O^{sp}$ (F-4) | 7 | | | |
| $NH^{b}$ (M43) | $O^{sp}$ (FC+5) | 7 | | | |
| $NH_2^{sr}$ (K99) | $O^{sp}$ (FC+6) | 7 | | | |
| $NH^{b}$ (T50) | $O^{sp}$ (F-3) | 6 | | | |
| $NH^{sr}$ (W75) | $O^{sp}$ (G9) | 5 | | | |
| $NH_2^{sr}$ (K99) | $O^{sp}$ (FC+6) | 5 | | | |
| $NH_2^{sr}$ (K91) | $N3^{nb}$ (G14) | 5 | | | |
| $NH^{b}$(M43) | $O^{sp}$ (FC+6) | 4 | | | |
| $NH_2^{sr}$ (K76) | $N3^{nb}$ (A8) | 4 | | | |
| $OH^{sr}$ (S42) | $O^{sp}$ (G1) | 4 | | | |
| $OH^{sr}$ (S42) | $O^{sp}$ (G1) | 4 | | | |
| $NH_2^{sr}$ (Q85) | $O6^{sp}$ (G9) | 3 | | | |
| $NH_2^{sr}$ (K55) | $O^{sp}$ (FC+8) | 3 | | | |
| $NH_2^{sr}$ (K102) | $O^{sp}$ (FC+6) | 2 | | | |

BD2 (white background):

| donor | acceptor | sn.% |
|---|---|---|
| $NH_2^{sr}$ (K378) | $O^{sp}$ (A12) | 60 |
| $NH_2^{sr}$ (K367) | $O^{sp}$ (G2) | 31 |
| $NH_2^{sr}$ (K367) | $O^{sp}$ (G3) | 28 |
| $NH_2^{sr}$ (K367) | $O^{sp}$ (G2) | 24 |
| $NH_2^{sr}$ (K368) | $N7^{nb}$ (FC-2) | 15 |
| $NH_2^{sr}$ (K445) | $O^{sp}$ (FC-5) | 10 |
| $NH_2^{sr}$ (R453) | $O^{sp}$ (FC-3) | 9 |
| $N6^{nb}$ (A8) | $O^{b}$ (S351) | 9 |
| $NH^{b}$ (K367) | $N7^{nb}$ (G3) | 9 |
| $NH_2^{sr}$ (R410) | $N7^{nb}$ (A8) | 8 |
| $N6^{nb}$ (A8) | $O^{sr}$ (S351) | 8 |
| $NH_2^{sr}$ (K445) | $O^{sp}$ (FC-4) | 7 |
| $NH^{b}$ (K367) | $N2^{nb}$ (G15) | 7 |
| $NH^{sr}$ (R410) | $N7^{nb}$ (A8) | 5 |
| $OH^{sr}$ (S358) | $O^{sp}$ (A8) | 5 |
| $NH_2^{sr}$ (K367) | $O4'^{sp}$ (F+2) | 4 |
| $NH_2^{sr}$ (K368) | $O4^{sp}$ (FC-1) | 4 |
| $NH_2^{sr}$ (K367) | $O4'^{sp}$ (F+1) | 2 |
| $NH_2^{sr}$ (K368) | $O^{sp}$ (FC-3) | 2 |
| $NH^{sr}$ (R444) | $O^{sp}$ (FC-5) | 2 |

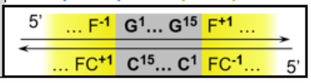

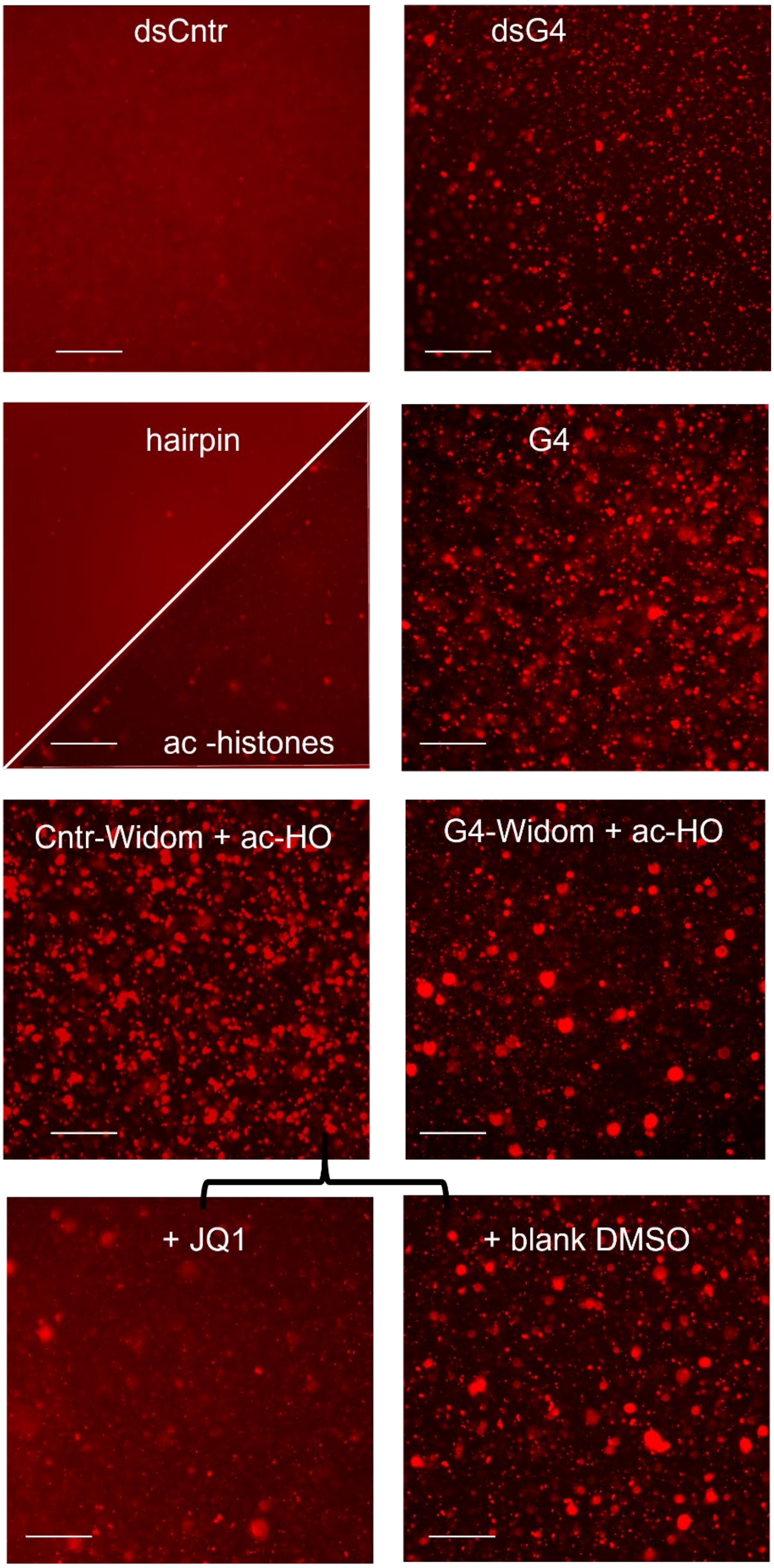


**Figure S13. Phase separation in BRD4 mixtures with G4, control DNA and respective nucleosomes: large-field fluorescent microscopy images.** Conditions: 4 μM BRD4 (15% labeled) and 4 μM dsCntr/dsG4/hairpin/G4 or 300 nM H3K18ac, or 150 nM Cntr-Widom/G4-Widom with 300 nM ac-HO in 1X PBS buffer (pH 7.4) supplemented 20% PEG-400 and 0.05% Tween-20. Samples with Cntr-Widom/G4-Widom also contained 2x-diluted nucleosome assembly buffer, i.e., 10 mM Tris-HCl (pH 8) and 125 mM NaCl. The bottom images were obtained for sample 'Cntr-Widom + ac-HO' after its incubation with 5 μM JQ1 or blank (0.5% DMSO) solutions. Scale bar: 50 μm.

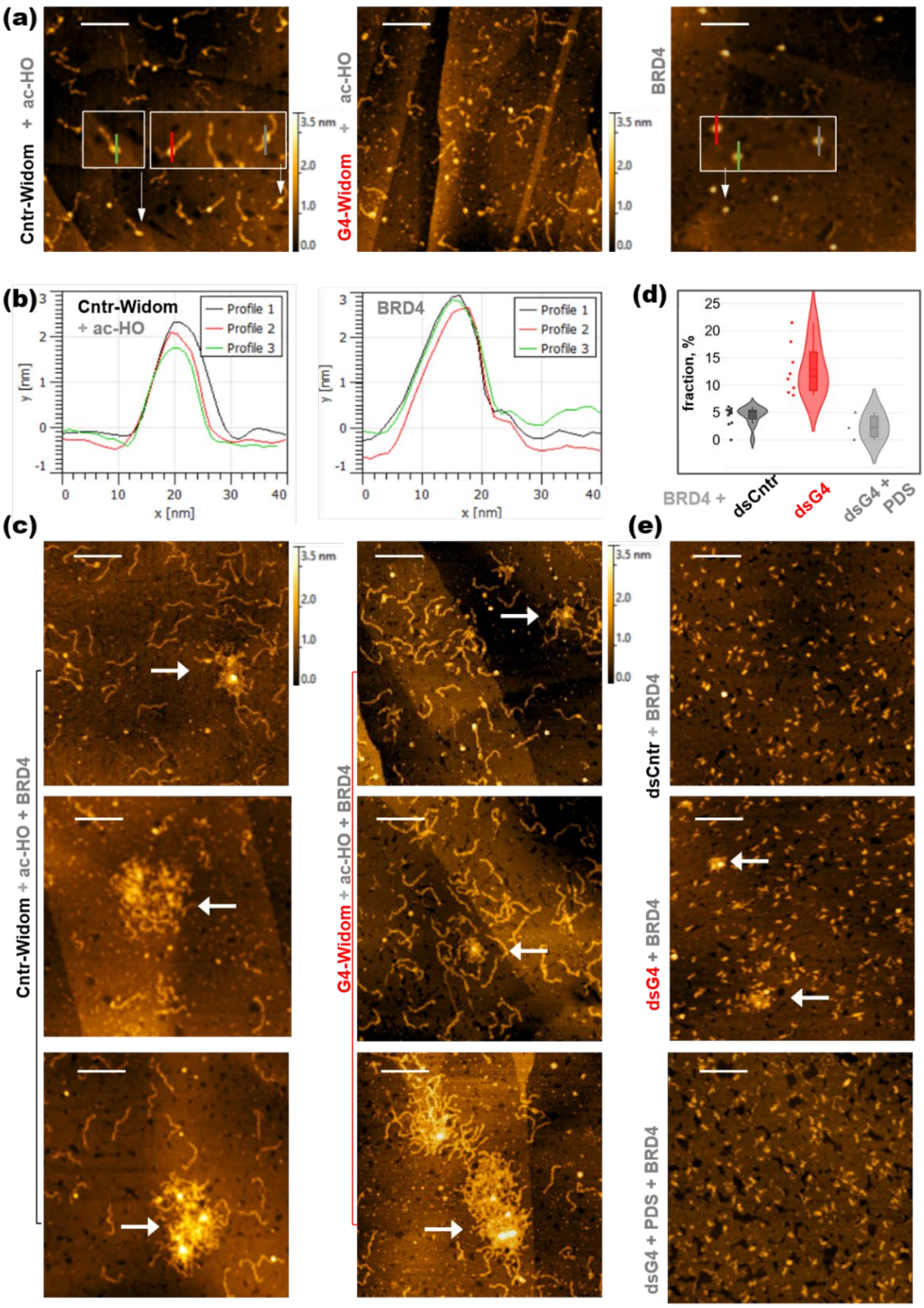


**Figure S14. Phase separation in BRD4 mixtures with G4, control DNA and respective nucleosomes: atomic force microscopy (AFM) images.** (**a**) AFM scans of stable/unstable nucleosomes and free BRD4. Left: 150 nM Cntr-Widom with 300 nM H3K18ac-containing histone octamer (ac-HO). Middle: 150 nM G4-Widom with 300 nM ac-HO. Right: 4 μM free BRD4. All samples were prepared in 10 mM Tris-HCl buffer (pH 8) containing 125 mM NaCl (diluted nucleosome assembly buffer, NAB) and supplemented with 20% PEG-400. Vertical grey/red/green marks are lines along which surface profiles were measured. (**b**) Surface profiles of Cntr-Widom/ac-HO and BRD4 samples. (**c**) AFM scans of BRD4 mixtures with stable (Cntr-Widom + ac-HO) or unstable (G4-Widom + ac-HO) nucleosomes in diluted NAB with 20% PEG-400. BRD4 concentration: 4 μM; other components: same as in (a). (**d**) Summary of the AFM-based analysis of BRD4 mixtures with dsCntr and dsG4 samples in the presence/absence of PDS. Conditions: 4 μM BRD4, 4 μM dsCntr/dsG4, and 0/20 μM PDS in diluted NAB supplemented with 20% PEG-400. The violin plots summarize apparent partitioning, i.e., the fraction of DNA molecules in aggregates. (**e**) Representative AFM scans of the samples described in (d). The aggregates are marked with arrows. Scale bar: 100 nm.

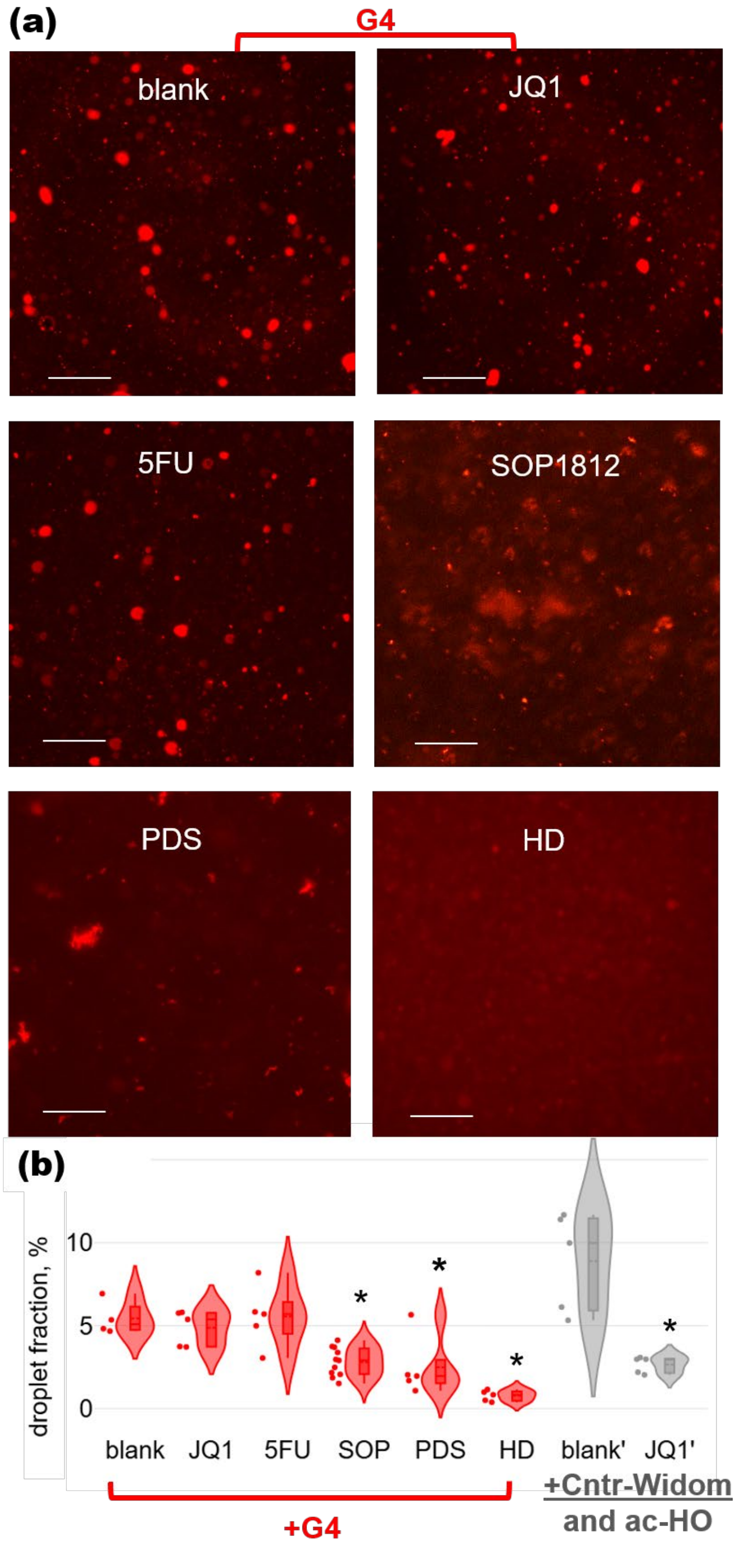


**Figure S15. Phase separation in BRD4-G4 mixtures: effects of BRD4/G4 ligands.** (**a**) Large-field fluorescent microscopy images. Conditions: 4 μM BRD4 (15% labeled) and 4 μM G4 in 1X PBS buffer (pH 7.4) supplemented with 20% PEG-400. Ligands were added in DMSO-containing solutions (0.5% in the final sample) to the following concentrations: 5 μM JQ1, 100 μM 5FU, 10% HD, 20 μM PDS, and 5 μM SOP1812. Scale bar: 50 μm. (**b**) Summary of ligands' effects on droplet fraction in BRD4-G4 mixtures (red) or BRD4 mixtures with Cntr-Widom and ac-HO (grey). * Significant difference from the blank (DMSO only) sample ($p < 0.05$, t-test for Cntr-Widom + ac-HO and one-way ANOVA with Dunnett's post-hoc test for G4)

**Table S6. Genes selected for the analysis of up/down regulation by PDS.**

| gene | Primers: forward (F); reverse (R) | $log_2$FC in K-562 (HEK-293)* | p-value, K-562 (HEK-293) |
|---|---|---|---|
| **main gene set** | | | |
| *PLK3* | F: tgtcggccaacagaaagact; R: ccgtagaagttcacctggaca | -1.30 (-0.35)<br>-2.10 (1.78) | 3.80E-04 (3.04E-02)<br>3.23E-04 (2.41E-04) |
| *IFI6* | F: tggggtggaggcaggtaag; R: gatcgcagaccagctcatca | -1.58 (0.30)<br>-0.97 (-1.23) | 2.71E-02 (2.83E-01)<br>5.34E-04 (6.62E-04) |
| *WASF2* | F: gctggggacttctgggtatc; R: ttctgctggtggaggaggc | -1.62 (-1.46)<br>-2.55 (1.06) | 5.00E-05 (1.38E-03)<br>1.00E-06 (2.00E-06) |
| *UROD* | F: ccgttacttaccagagtttaggga; R: cgttgtcgggtaagggtgat | -2.27 (-1.49)<br>0.07 (1.41) | 2.67E-04 (6.40E-05)<br>4.85E-01 (1.13E-04) |
| *RAPGEFL1* | F: tttgagaacctgacggaccc; R: aggcacgaagggaatcacag | -1.68 (-3.04)<br>-2.42 (-0.29) | 6.12E-03 (7.31E-04)<br>1.53E-02 (1.49E-02) |
| *MKNK2* | F: gcagaagaaaccagccgaac; R: tgcaggccaaagtcagagtc | -2.99 (-2.03)<br>-1.2 (-0.21) | 4.30E-05 (3.32E-04)<br>3.88E-02 (9.00E-01) |
| *NR1D1* | F: aacaacaacacaggtggcgtc; R: gggtcttgggtgagggagc | -4.09 (-2.26)<br>-3.37 (1.23) | 6.00E-06 (9.96E-04)<br>1.89E-03 (4.89E-0.2) |
| *DOT1L* | F: gacttggggagcggtgtg; R: aatcgcctctctccaatgtgtat | -4.37 (-2.76)<br>-1.24 (0.71) | 1.30E-05 (8.80E-05)<br>7.59E-03 (6.31E-03) |
| *STX12* | F: tcgtcatgtcatacggtccc; R: ctttatctgagcagtggcttgg | -1.58 (-1.38)<br>-1.45 (0.68) | 1.00E-06 (3.10E-05)<br>1.35E-04 (5.01E-03) |
| *PSMD3* | F: cgcctcaaccactatgttctg; R: ggacggaactgtaaatcagcc | 0.48 (-0.40)<br>0.23 (1.20) | 5.12E-03 (1.57E-03)<br>3.12E-02 (2.73E-0.2) |
| **reference** | | | |
| *RPLP0* | F: gcgggaaggctgtggtg; R: atatgaggcagcagtttctcca | N/A | N/A |
| *RPS18* | F: cactgaggatgaggtggaacg; R: gaagtgacgcagccctctatg | N/A | N/A |
| **additional gene set** | | | |
| *TOE1* | F: aagcacaacatctggggagga; R: tggtcaatgcactggttcagc | -1.67 (-1.13) | 1.50E-05 (1.21E-03) |
| *PVR* | F: cctcaccgtgtactaccccc; R: gcattggtgacgttgcagat | -3.05 (-2.45) | 3.67E-03 (2.00E-05) |
| *SLC6A9* | F: gcctccaacctcaccaatg; R: gaggaagacgaccaaccagg | -3.70 (-2.12) | 3.66E-04 (2.00E-06) |
| *ERCC1* | F: caagaccagcaggctccaga; R: tgagatggcatattcggcg | -5.29 (-5.20) | 2.32E-03 (1.77E-03) |
| *ERCC2* | F: gctcctggtctacttcccgt; R: ggtgaatacacaagtttttgcg | -7.73 (-6.10) | 1.26E-04 (4.94E-04) |
| *ELL* | F: caggattctgtttcactgaggc; R: cttgtcctgtatgctgccca | -5.60 (-5.18) | 3.22E-03 (1.49E-03) |
| *CRTC1* | F: acaatcagaagcaggcggag; R: gattggaagggggtctggaa | -4.01 (-5.86) | 2.21E-03 (7.10E-05) |
| *MAST3* | F: cgccgaggacgtggtct; R: gactatccaaggggctgctg | -5.97 (-3.50) | 2.71E-04 (7.10E-01) |
| *SAE1* | F: ctgaccatgctggatcacga; R: gccaacagacccagtacgaat | -6.93 (-6.85) | 1.00E-06 (9.30E-05) |
| *BBC3* | F: tcgcccgtgcccagc; R: cctcttgtctccgccgctc | -9.26 (-4.59) | 3.67E-04 (3.89E-03) |

* PDS, black; SOP1812, blue.

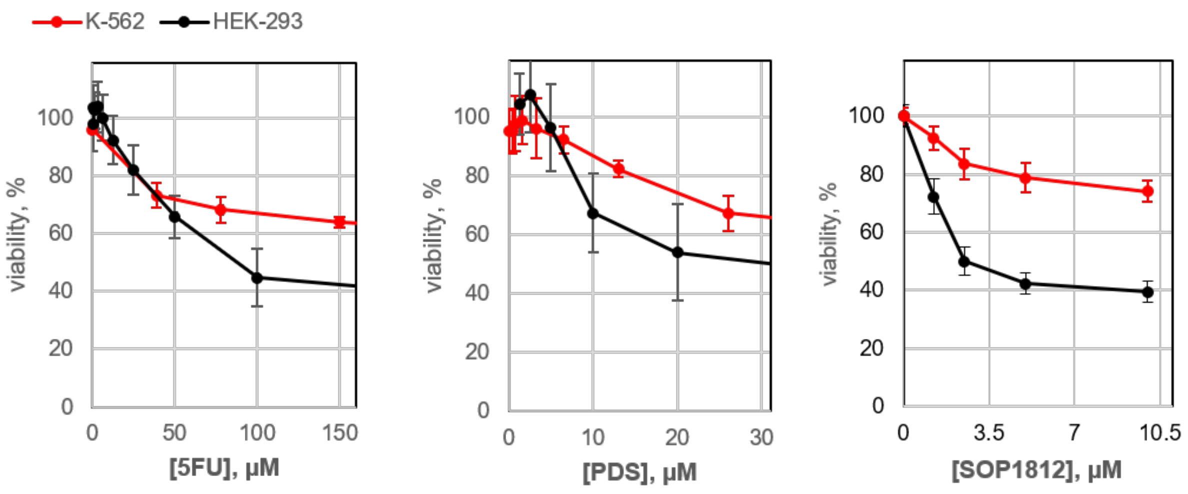


**Figure S16. Cellular toxicity of the G4 ligands (PDS and SOP1812) and the control compound (5FU).** Viability of K-562 or HEK-293 cells assessed using Presto-blue assays following 48 h incubation with 5FU, PDS, SOP1812, or blank solutions.
